# TM4SF1-AS1 inhibits apoptosis by promoting stress granule formation in cancer cells

**DOI:** 10.1038/s41419-023-05953-3

**Published:** 2023-07-13

**Authors:** Hiroshi Kitajima, Reo Maruyama, Takeshi Niinuma, Eiichiro Yamamoto, Akira Takasawa, Kumi Takasawa, Kazuya Ishiguro, Akihiro Tsuyada, Ryo Suzuki, Gota Sudo, Toshiyuki Kubo, Kei Mitsuhashi, Masashi Idogawa, Shoichiro Tange, Mutsumi Toyota, Ayano Yoshido, Kohei Kumegawa, Masahiro Kai, Kazuyoshi Yanagihara, Takashi Tokino, Makoto Osanai, Hiroshi Nakase, Hiromu Suzuki

**Affiliations:** 1 Department of Molecular Biology, Sapporo Medical University School of Medicine, Sapporo, Japan; 2 Project for Cancer Epigenomics, Cancer Institute, Japanese Foundation for Cancer Research, Tokyo, Japan; 3 Cancer Cell Diversity Project, NEXT-Ganken Program, Japanese Foundation for Cancer Research, Tokyo, Japan; 4 Department of Pathology, Sapporo Medical University School of Medicine, Sapporo, Japan; 5 Department of Gastroenterology and Hepatology, Sapporo Medical University School of Medicine, Sapporo, Japan; 6 Department of Medical Genome Sciences, Research Institute for Frontier Medicine, Sapporo Medical University School of Medicine, Sapporo, Japan; 7 Division of Rare Cancer Research, National Cancer Center Research Institute, Tokyo, Japan

**Keywords:** Gastric cancer, Oncogenes, Long non-coding RNAs, Apoptosis

## Abstract

Long noncoding RNAs (lncRNAs) play pivotal roles in tumor development. To identify dysregulated lncRNAs in gastric cancer (GC), we analyzed genome-wide trimethylation of histone H3 lysine 4 (H3K4me3) to screen for transcriptionally active lncRNA genes in the non-tumorous gastric mucosa of patients with GC and healthy individuals. We found that H3K4me3 at TM4SF1-AS1 was specifically upregulated in GC patients and that the expression of TM4SF1-AS1 was significantly elevated in primary and cultured GC cells. TM4SF1-AS1 contributes to GC cell growth in vitro and in vivo, and its oncogenic function is mediated, at least in part, through interactions with purine-rich element-binding protein α (Pur-α) and Y-box binding protein 1 (YB-1). TM4SF1-AS1 also activates interferon signaling in GC cells, which is dependent on Pur-α and RIG-I. Chromatin isolation by RNA purification (ChIRP)-mass spectrometry demonstrated that TM4SF1-AS1 was associated with several stress granule (SG)-related proteins, including G3BP2, RACK1, and DDX3. Notably, TM4SF1-AS1 promoted SG formation and inhibited apoptosis in GC cells by sequestering RACK1, an activator of the stress-responsive MAPK pathway, within SGs. TM4SF1-AS1-induced SG formation and apoptosis inhibition are dependent on Pur-α and YB-1. These findings suggested that TM4SF1-AS1 contributes to tumorigenesis by enhancing SG-mediated stress adaptation.

## Introduction

Long noncoding RNAs (lncRNAs) are involved in a wide range of biological processes, including the development, differentiation, and maintenance of homeostasis, as well as in various disorders, including cancer [1, 2]. A growing number of studies have reported the dysregulated expression of lncRNAs in cancer, although their involvement in the early stages of tumorigenesis and in precancerous lesions is not well documented. We thus hypothesized that screening for aberrantly expressed lncRNAs within lesions at a high risk of cancer development would be an effective way to identify lncRNAs causally related to tumorigenesis.

Chronic inflammation is strongly associated with a risk of carcinogenesis in various organs [3]. We and others have shown that chronic inflammation induces aberrant DNA methylation in the stomach and that DNA methylation can be a predictive biomarker of gastric cancer (GC) risk [4, 5]. Epigenetic alterations, including aberrant DNA methylation and histone modifications, are deeply involved in human malignancies [6]. Histone modifications are strongly associated with gene regulation under both physiological and pathological conditions. For instance, trimethylation of histone H3 lysine 4 (H3K4me3) is a marker of active transcription start sites (TSSs), whereas H3K9me2/3 and H3K27me3 are associated with a closed chromatin structure and transcriptional silencing [6].

Recent studies have also shown that phase separation triggered by the self-assembly of proteins and RNA molecules leads to the formation of ribonucleoprotein (RNP) granules, membraneless cellular compartments that include nucleoli, Cajal bodies, paraspeckles, processing bodies, and stress granules (SGs) [7, 8]. SGs are cytoplasmic foci composed of untranslated messenger RNAs (mRNAs), stalled translation pre-initiation complexes, and specific RNA-binding proteins (RBPs), which are assembled when untranslated messenger ribonucleoproteins (mRNPs) accumulate in cells subjected to stress [9, 10]. SGs are thought to be part of a cellular defense mechanism against various stressors, including heat shock, hypoxia, oxidative stress, and viral infections [9, 10]. Although the biological functions of SGs are not fully understood, they are reportedly associated with neurodegeneration, viral infections, inflammatory diseases, and cancer [10]. Emerging evidence suggests that reinforcement of stress-adaptive mechanisms is a strategy used by cancer cells to survive in hostile cancer microenvironment [11–13].

In the present study, we aimed to identify the lncRNAs that contribute to tumorigenesis in chronic inflammation-associated precancerous lesions. To this end, we performed epigenome analysis to screen for transcriptionally active lncRNAs within the gastric mucosa of patients with GC and identified a series of lncRNAs, including TM4SF1-AS1. Our findings suggest that TM4SF1-AS1 contributes to oncogenesis by inducing SG formation and inhibiting apoptosis.

## Materials and methods

### Tissue samples and cell lines

Biopsy specimens of non-tumorous gastric mucosa were obtained from the gastric antrum of four Japanese GC patients and three healthy Japanese individuals who underwent upper gastrointestinal endoscopy at Sapporo Medical University Hospital. This study was approved by the institutional review board of Sapporo Medical University (No. 29-18). Informed consent was obtained from all participants prior to specimen collection. For the chromatin immunoprecipitation-sequencing (ChIP-seq) experiments, crypt isolation was performed to obtain an epithelial cell-enriched fraction [14]. For targeted sequencing, biopsy specimens of paired tumor and non-tumorous gastric mucosa were obtained from eight Japanese patients with GC. Gastric cancer cell lines (HSC-45, MKN45, SNU1, and SNU638) were cultured as previously described [15, 16]. Cell lines were authenticated using short tandem repeat analysis (JCRB, Tokyo, Japan; BEX, Tokyo, Japan). Mycoplasma infection was negative in the above cell lines, as confirmed using the EZ-PCR Mycoplasma Detection Kit (Biological Industries, Beit HaEmek, Israel).

### RNA extraction, fractionation, and quantitative reverse transcription-PCR

Total RNA was prepared using an RNeasy Mini kit (Qiagen, Hilden, Germany) or TRI reagent (Molecular Research Center, Inc., Cincinnati, OH, USA), according to the manufacturer’s instructions. Nuclear and cytoplasmic RNAs were isolated using a Cytoplasmic and Nuclear RNA Purification kit (Norgen Biotek, Thorold, Canada). Single-stranded cDNA synthesis and quantitative RT-PCR were performed as described previously [17]. ACTB (β-actin) was used as an endogenous control for total and cytoplasmic RNA and U6 snRNA was used as an endogenous control for nuclear RNA. The primer sequences used are listed in Supplementary Table S1.

### Chromatin immunoprecipitation sequencing

Genome-wide H3K4me3 was analyzed using ChIP-seq, as described previously [18]. Sequencing data were mapped to the human genome (hg19) using Bowtie2 and peaks were defined using MACS2. An annotation dataset for human lncRNA genes was obtained from the Human Body Map of lincRNAs at the Broad Institute [19]. Genes were defined as transcriptionally active when H3K4me3 peaks overlapped regions encompassing putative transcription start sites (TSSs; −0.5 kb to +0.5 kb relative to TSSs).

### Plasmid vectors

pCMV6 encoding GFP-fused TM4SF1 was purchased from OriGene Technologies (Rockville, MD, USA). pIDTSMART-AMP encoding TM4SF1-AS1 (ENST496491.1) was purchased from Codex DNA Inc. (San Diego, CA, USA). Fragments of TM4SF1-AS1 and the neomycin resistance cassette were cloned into pBI-CMV2 (Takara Bio Inc., Kusatsu, Japan). Full-length cDNAs encoding Pur-α and YB-1 were amplified with PCR using cDNA derived from HSC-45 cells as a template and then cloned into pCMV6-Entry with a C-terminal Myc tag or FLAG tag (OriGene Technologies) or into pCMV6-AC GFP (OriGene Technologies). shRNAs targeting TM4SF1-AS1 were designed using the siDirect algorithm (http://sidirect2.rnai.jp/) and were cloned into Tet-pLKO-puro (a gift from Dr. Dimitri Wiederschain, plasmid #21915, Addgene, Watertown, MA, USA). TM4SF1-AS1 and 12×MS2 were amplified with PCR, respectively using pBI-CMV2 NeoR TM4SF1-AS1 plasmid and CFP betaGlobin 24×PP7 24×MS2 splicing reporter (a gift from Dr. Daniel Larson; Addgene plasmid #61762) as templates. The fragments were cloned into pCW57.1 (a gift from Dr. David Root, plasmid #41393, Addgene). ptagRFP-MS2coatProtein plasmid was a gift from Dr. Karsten Rippe (plasmid #103831, Addgene). Primer sequences are listed in Supplementary Table S1.

### Xenograft studies

HSC-45 cells with inducible shRNA (1.5 × 10^6^ cells) or SUN638 cells stably expressing TM4SF1-AS1 or GFP (1.0 × 10^7^ cells) were resuspended in 100 μl of phosphate-buffered saline (PBS). Cells mixed with 100 μl of Matrigel basement matrix (Corning Inc. Corning, NY, USA) were subcutaneously injected into the bilateral thighs of 6-week-old female BALB/cAJcl-nu mice (four mice per group). All mice were randomly divided into treatment groups or control groups. Mice in the inducible shRNA group were fed either a diet containing 200 ppm Dox or a regular diet. Tumor size was measured every 4 days using digital calipers, and tumor volume was calculated using the formula: length × width^2^/2. Immunohistochemistry was performed using a rabbit anti-cleaved caspase-3 mAb (1:500 dilution, #9664, Cell Signaling Technology, Danvers, MA, USA) and a rabbit anti-cleaved PARP mAb (1:200 dilution, #5625, Cell Signaling Technology) as described previously [20]. Apoptosis was also analyzed using a MEBSTAIN Apoptosis TUNEL Kit Direct (MBL Co. Ltd, Nagoya, Japan). To analyze SGs, xenograft tumors were resected, cryosectioned, and subjected to immunofluorescent staining with a rabbit anti-G3BP2 pAb (1:200 dilution, 16276-1-AP, Proteintech). All animal experiments were approved by the Institutional Animal Ethical Committee of Sapporo Medical University (No. 19-028, No. 17-089, No. 23-044). No statistical method was used to predetermine the sample size for the xenograft studies, and it was based on previous experimental observation. No data were excluded from the analysis. No blinding was done in the animal studies.

### Statistical analysis

All data were obtained from at least three independent experiments and data are presented as the mean ± SDs. The sample size (*n*) for each experiment is indicated in the figure legends. Quantitative variables were analyzed using Student’s *t* tests (two-sided) or ANOVA with post hoc Tukey’s tests when the variance was similar between the groups. Values of P < 0.05 (two-sided) were considered statistically significant. Statistical analyses were carried out using EZR version 1.32 (Saitama Medical Center, Saitama, Japan) [21].

More detailed methods are described in Supplementary Methods.

## Results

### Detection of TM4SF1-AS1 upregulation in GC patients

**Fig. 1:**
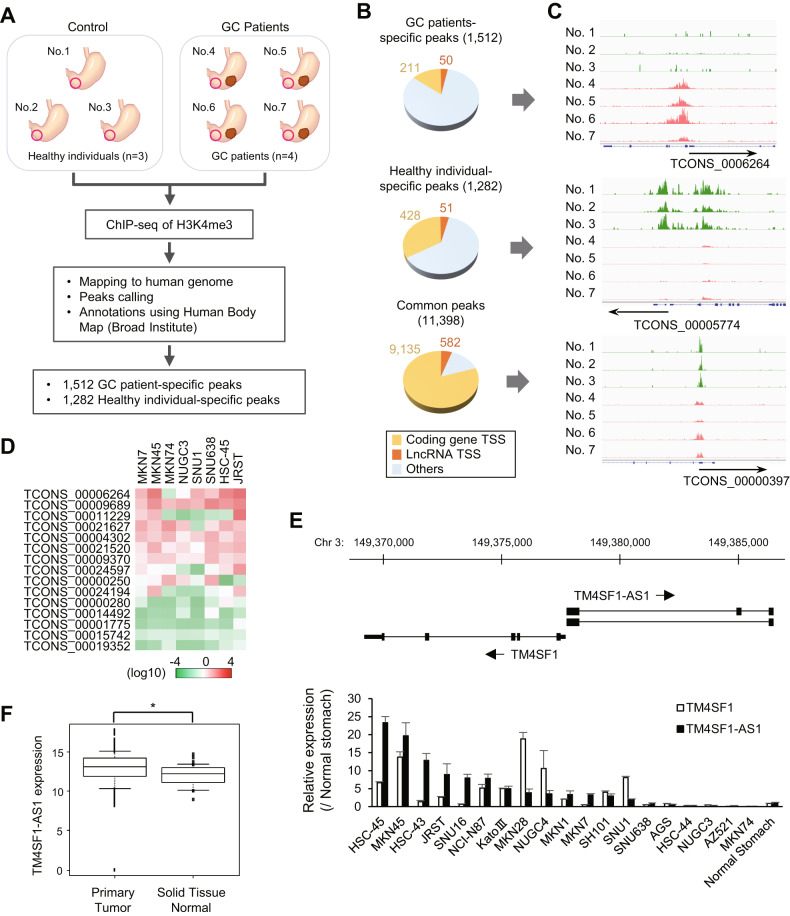
Screening for lncRNAs transcriptionally activated in the gastric mucosa of patients with gastric cancer (GC). **A** Workflow for comprehensive identification of transcriptionally active lncRNAs in the gastric mucosa of patients with GC. **B** Pie chart depicting gene loci harboring H3K4me3 peaks at transcription start sites (TSSs). These loci were categorized as protein-coding genes, lncRNA genes, or other genes. Peaks were categorized as patient-specific, healthy individual-specific, and common. **C** H3K4me3 peaks of representative genes in each category. **D** Summarized qRT-PCR results for 15 selected lncRNAs from eight GC cell lines. **E** qRT-PCR analysis of TCONS_00006264 (TM4SF1-AS1) and TM4SF1 in GC cell lines and normal stomach tissue. The locations of these two genes are shown at the top. (*n* = 3). **F** Expression of TM4SF1-AS1 in primary GCs (*n* = 374) and normal stomach tissues (*n* = 32) from The Cancer Genome Atlas (TCGA) dataset. *P < 0.05.

To identify lncRNAs associated with chronic inflammation and tumorigenesis in the stomach, we first searched for lncRNAs upregulated in the gastric mucosa of patients with GC. We collected specimens of gastric mucosa from three healthy individuals and non-tumorous gastric mucosa from four GC patients (Fig. 1A). Because lncRNAs are often characterized by multiple transcription start sites and transcription variants, we took advantage of histone modifications representing transcription states. Chromatin immunoprecipitation-sequencing (ChIP-seq) analysis of H3K4me3 enabled identification of active TSSs in lncRNA genes (Fig. 1A). We detected a series of 11,398 H3K4me3 peaks commonly observed in both healthy individuals and GC patients (Fig. 1B). In addition, 1,512 peaks were specifically detected in GC patients and 1282 were specific to healthy individuals (Fig. 1A–C). Among these, 50 and 51 peaks were associated with lncRNA genes, respectively (Fig. 1B, C and Supplementary Tables S2 and S3).

Among the 50 lncRNAs potentially associated with GC, we confirmed the expression of 15 lncRNAs in eight GC cell lines. qRT-PCR analysis revealed that most of these lncRNAs were expressed in multiple cell lines (Fig. 1D). We noted that TCONS_00006264 (also known as TM4SF1-AS1) was abundantly expressed in most of the GC cell lines tested (Fig. 1D). TM4SF1-AS1 is located on chromosome 3q25.1, and is transcribed in an antisense direction relative to a neighboring gene, transmembrane 4L six family member 1 (TM4SF1), which is overexpressed in multiple human malignancies (Fig. 1E) [22]. qRT-PCR analysis of a series of 19 GC cell lines revealed that 13 cell lines expressed TM4SF1-AS1 at higher levels than normal stomach tissue (Fig. 1E). RNA-seq data from The Cancer Genome Atlas (TCGA) revealed that TM4SF1-AS1 expression levels were higher in primary GC tissues than in normal stomach tissues, though they were not associated with clinical stage (Fig. 1F and Supplementary Fig. S1A, B). Kaplan–Meier curve analysis showed that expression levels of TM4SF1-AS1 did not correlate with the overall survival of GC patients (Supplementary Fig. S1C). We also assessed whether TM4SF1-AS1 is a target of genetic alterations in GCs. Targeted sequencing of paired samples of tumor and corresponding normal stomach tissues from eight GC patients revealed a novel variant in the intronic region of TM4SF1-AS1 in the GC tissue (Supplementary Table S4). These results suggested that TM4SF1-AS is associated with gastric tumorigenesis.

### TM4SF1-AS1 promotes GC cell growth

**Fig. 2:**
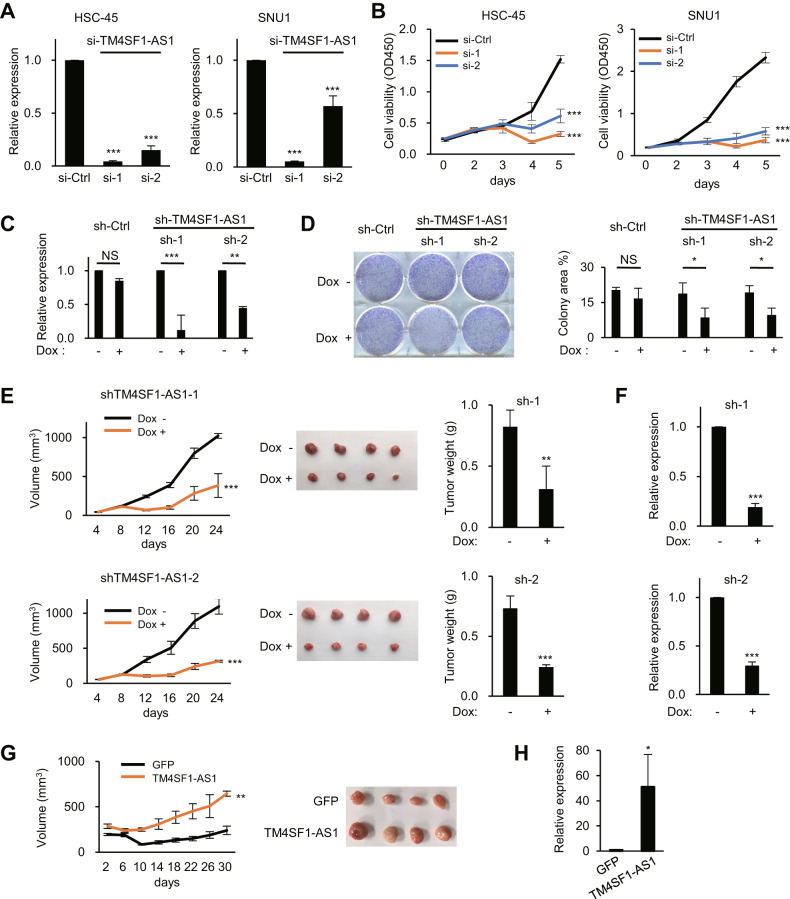
TM4SF1-AS1 promotes GC cell growth. **A** qRT-PCR analysis of TM4SF1-AS1 in the indicated GC cell lines transfected with siRNAs targeting TM4SF1-AS1 (si-1 and 2) or a control siRNA (si-Ctrl). (*n* = 3). **B** Results of cell viability assays with GC cell lines transfected with the indicated siRNAs. (*n* = 8). **C** qRT-PCR analysis of TM4SF1-AS1 in HSC-45 cells with inducible shRNAs targeting TM4SF1-AS1 (sh-1 and 2) or a control shRNA (sh-Ctrl). Cells were incubated for 8 days with or without doxycycline (Dox). (*n* = 3). **D** Colony formation assays using HSC-45 cells with inducible shRNAs. Cells were incubated for 8 days with or without Dox. Summarized results are shown on the right; error bars represent SDs. (*n* = 3). **E** Tumor growth in mice injected with HSC-45 cells inducibly expressing the indicated shRNAs. Mice were treated with or without Dox. Growth curves (left), resected tumors (middle), and tumor weights (right) are shown. **F** qRT-PCR analysis of TM4SF1-AS1 in the resected tumors in **E**. (*n* = 4). **G** Tumor growth in mice injected with SNU638 cells stably expressing GFP or TM4SF1-AS1. Growth curves are shown on the left, and resected tumors are shown on the right. (*n* = 4). **H** qRT-PCR analysis of TM4SF1-AS1 in the resected tumors in **G**. (*n* = 4). *P < 0.05, **P < 0.01, ***P < 0.001, NS not significant.

We next attempted to clarify the role of TM4SF1-AS1 in GC development. TM4SF1-AS1 knockdown significantly suppressed GC cell viability but had no effect on GC cell migration or invasion (Fig. 2A, B; and Supplementary Fig. S2A, B; data not shown). In contrast, TM4SF1 knockdown suppressed GC cell viability as well as their migration and invasion (Supplementary Fig. S2C-E). Using GC cells expressing Dox-inducible shRNAs targeting TM4SF1-AS1 (HSC-45-tet-shTM4SF1-AS1), we observed that TM4SF1-AS1 depletion suppressed colony formation (Fig. 2C-D). In addition, Dox administration to mice inoculated with HSC-45-tet-shTM4SF1-AS1 cells significantly attenuated tumor formation (Fig. 2E). The reduced levels of TM4SF1-AS1 in tumors from Dox-treated mice were confirmed using qRT-PCR (Fig. 2F). We also established GC cells stably overexpressing TM4SF1-AS1 (SNU638-TM4SF1-AS1) or GFP (SNU638-GFP) and found that TM4SF1-AS1 significantly promoted xenograft tumor formation (Fig. 2G, H). These results suggest that TM4SF1-AS1 exerts oncogenic effects on GC.

### Co-expression of TM4SF1 and TM4SF1-AS1 in GC cells

Earlier studies have shown that lncRNA genes are often oriented in an antisense direction relative to their host genes and that lncRNAs regulate their host genes through cis-regulatory functions [23]. As shown in Fig. 1E, a number of GC cell lines expressed both TM4SF1 and TM4SF1-AS1 at higher levels than a normal stomach tissue. Moreover, analysis using TCGA datasets revealed that the expression levels of these two genes were positively correlated in primary GC tissues (Supplementary Fig. S3A). Using flow cytometry, TM4SF1 protein was detected on the surface of HSC-45 cells, in which TM4SF1-AS1 was highly expressed (Supplementary Fig. 3B). In contrast, TM4SF1 was not detected on the surface of SNU638 cells where TM4SF-AS1 expression was limited (Supplementary Fig. S3B). These results suggest that the expression levels of TM4SF1-AS1 and TM4SF1 are highly concordant in GC cells and that TM4SF1-AS1 may positively regulate TM4SF1 expression. However, TM4SF1-AS1 knockdown in HSC-45 cells did not affect TM4SF1 expression (Supplementary Fig. S3C). Similarly, ectopic expression of TM4SF1-AS1 in SNU638 cells did not upregulate TM4SF1 expression (Supplementary Fig. S3D). This suggests that TM4SF1 and TM4SF1-AS1 may be transcriptionally activated through a common mechanism in GC cells, although TM4SF1-AS1 may not directly regulate TM4SF1 expression.

### TM4SF1-AS1 interacts with Pur-α and YB-1

**Fig. 3:**
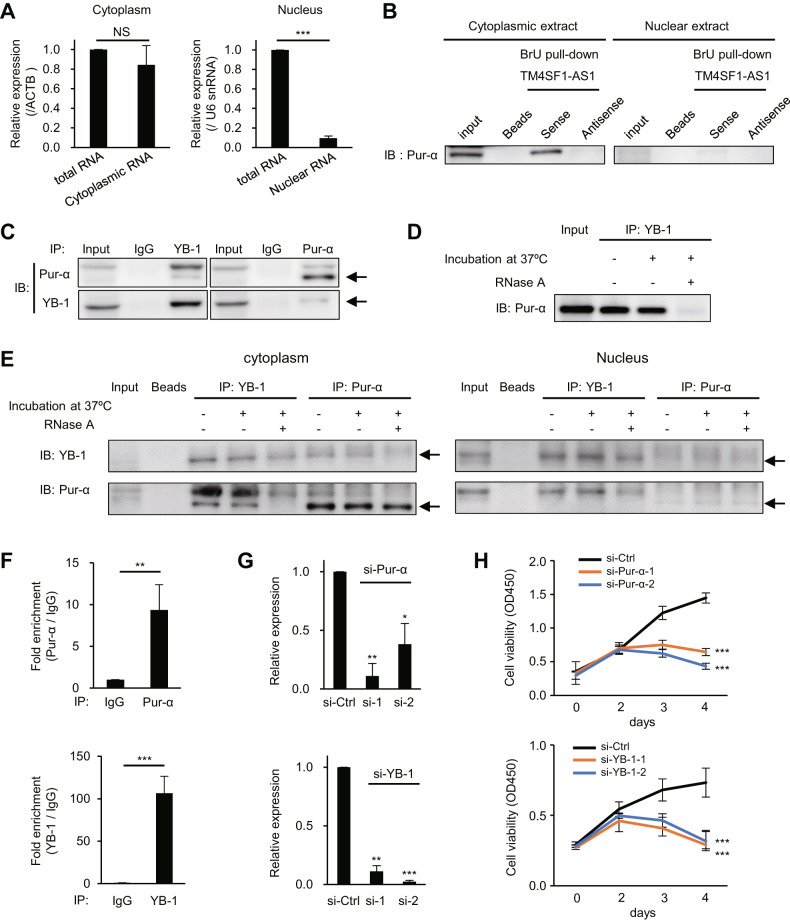
Identification of proteins that interact with TM4SF1-AS1. **A** qRT-PCR analysis of TM4SF1-AS1 in the indicated cellular fractions from HSC-45 cells. U6 snRNA and ACTB served as endogenous controls. (*n* = 3). **B** Association between TM4SF1-AS1 and Pur-α in the indicated subcellular fractions from HSC-45 cells. Pur-α was detected by western blotting in proteins pulled-down with BrU-labeled TM4SF1-AS1, antisense of TM4SF1-AS1 or beads. **C** Association between Pur-α and YB-1. Immunoprecipitated YB-1 or Pur-α from HSC-45 cell extracts were probed for co-precipitating proteins. **D** YB-1 was immunoprecipitated from HSC-45 cells with or without RNase A treatment, after which Pur-α was detected by western blotting. **E** YB-1 or Pur-α in the indicated subcellular fractions from HSC-45 cells were immunoprecipitated with or without RNase A treatment and then probed for co-precipitating proteins. **F** Results of RIP assays. Pur-α (upper) or YB-1 (lower) was immunoprecipitated from HSC-45 cells, after which co-precipitated TM4SF1-AS1 was detected with qRT-PCR. IgG served as a negative control. (*n* = 3). **G** qRT-PCR analysis of Pur-α (upper) and YB-1 (lower) in HSC-45 cells transfected with the indicated siRNAs. (*n* = 3). **H** Results of cell viability assays with HSC-45 cells transfected with the indicated siRNAs. (*n* = 3). *P < 0.05, **P < 0.01, ***P < 0.001.

To further explore the molecular function of TM4SF1-AS1, we assessed its intracellular localization. qRT-PCR analysis of the nuclear and cytoplasmic fractions from HSC-45 cells suggested that they were enriched in the cytoplasm (Fig. 3A). RNA pull-down and mass spectrometry analyses revealed a series of 16 proteins (RBMX, Pur-α, Pur-β, HNRNPH3, SPOUT1, RBMX, ILF2, BX1, HNRNPC, HNRPA1, YB-1, HNRNPF, ELAVL1, SRSF7, RBMXL1, and AHNAK) that potentially interacted with TM4SF1-AS1 (Supplementary Fig. S4A, B and Supplementary Table S5). Western blot analysis of the pull-down products confirmed an association between Pur-α and BrU-labeled or biotin-labeled TM4SF1-AS1 (Fig. 3B; Supplementary Fig. S4C). Although RBMX and Pur-β showed high scores in the mass spectrometry results, western blot analyses failed to confirm their interaction with TM4SF1-AS1 (Supplementary Fig. S4D). Mass spectrometry also suggested that YB-1, a well-characterized RNA-binding protein, potentially interacts with TM4SF1-AS1 (Supplementary Table S5). We found that Pur-α interacts with YB-1 in GC cells and that this interaction was inhibited by RNase A treatment, suggesting that it is dependent on RNA molecules in the cytoplasm (Fig. 3C–E). TM4SF1-AS1 knockdown did not significantly affect the Pur-α-YB-1 interaction, suggesting that multiple RNA molecules may be involved in the Pur-α-YB-1-RNA complex (Supplementary Fig. S4E).

RNA immunoprecipitation (RIP)-qPCR assays further confirmed the interaction between endogenous TM4SF1-AS1 and Pur-α or YB-1 (Fig. 3F). To identify the domain within TM4SF1-AS1 required for its interaction with Pur-α and YB-1, we constructed a series of BrU-labeled TM4SF1-AS1 deletion mutants and performed RNA pull-down and western blot analyses (Supplementary Fig. S4F). All the deletion mutants showed various levels of interaction with Pur-α (Supplementary Fig. S4F). Similarly, all but one mutant (451-600 nt) interacted with YB-1 (Supplementary Fig. S4F). This suggests that the interaction between TM4SF1-AS1 and these proteins is not dependent on a specific domain or region. Finally, we found that Pur-α or YB-1 knockdown significantly attenuated growth of GC cells expressing TM4SF1-AS1 (Fig. 3G, H and Supplementary Fig. S5). By contrast, depletion of Pur-α or YB-1 did not affect growth of GC cells not expressing TM4SF1-AS1 (SNU638-GFP, Supplementary Fig. S5). Thus, the oncogenicity of TM4SF1-AS1 may be mediated, at least in part, by its interaction with Pur-α and YB-1.

### TM4SF1-AS1 activates interferon signaling in GC cells

**Fig. 4:**
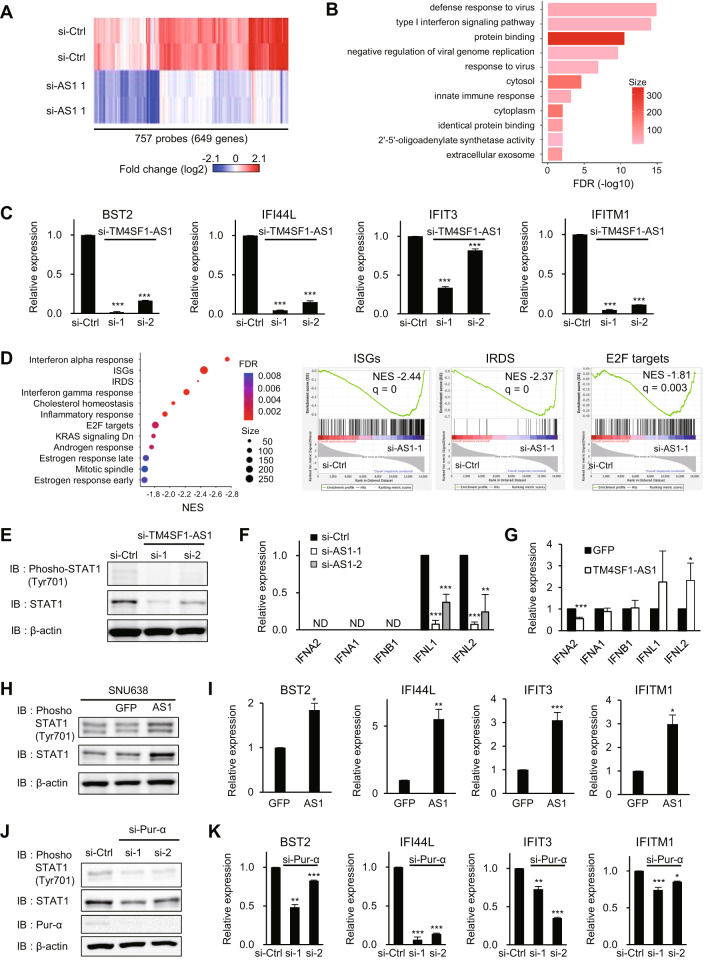
TM4SF1-AS1 activates interferon signaling in GC cells. **A** Heatmap showing expression of genes suppressed by TM4SF1-AS1 knockdown in HSC-45 cells. **B** GO analysis using the genes in **A**. **C** qRT-PCR analysis of representative IRDS genes in HSC-45 cells transfected with the indicated siRNAs. (*n* = 3). **D** GSEA analysis using the microarray data. Gene sets with an FDR < 0.01 are shown on the left. Enrichment plots of indicated gene sets are shown on the right. **E** Western blot analysis of total and phosphorylated STAT1 in HSC-45 cells transfected with the indicated siRNAs. (F, G) qRT-PCR analysis of interferon genes in HSC-45 cells transfected with the indicated siRNAs (**F**) or in SNU638 cells stably transfected with the indicated genes (**G**). (*n* = 3). **H** Western blot analysis of total and phosphorylated STAT1 in SNU638, SNU638-GFP, and SNU638-TM4SF1-AS1 cells. **I** qRT-PCR analysis of IRDS genes in SNU638-GFP and SNU638-TM4SF1-AS1 cells. (*n* = 3). **J** Western blot analysis of STAT1 and Pur-α in SNU638-TM4SF1-AS1 cells transfected with the indicated siRNAs targeting Pur-α. **K** qRT-PCR analysis of IRDS genes in SNU638-TM4SF1-AS1 cells transfected with the indicated siRNAs. (*n* = 3). *P < 0.05, **P < 0.01, ***P < 0.001.

To identify the downstream targets of TM4SF1-AS1, we performed a gene expression microarray analysis of HSC-45 cells with or without TM4SF1-AS1 knockdown. We found that 745 probes (591 unique genes) were upregulated, whereas 757 probes (649 unique genes) were downregulated (2-fold, *P* < 0.05) by TM4SF1-AS1 knockdown (Fig. 4A and Supplementary Table S6). Gene ontology (GO) analysis revealed that genes associated with interferon signaling and immune responses were significantly enriched among the downregulated genes (Fig. 4B). qRT-PCR analysis confirmed the significant suppression of representative interferon-related DNA damage resistance signature (IRDS) genes and interferon-stimulated genes (ISGs) after TM4SF1-AS1 depletion in GC cells (Fig. 4C and Supplementary Fig. S6A). Gene set enrichment analysis (GSEA) also showed that genes associated with interferon responses or IRDS were downregulated by TM4SF1-AS1 knockdown (Fig. 4D and Supplementary Fig. S6B). In addition, genes associated with cell proliferation, including E2F targets and KRAS signaling-related genes, were enriched among the downregulated genes (Fig. 4D and Supplementary Fig. S6B). Moreover, the levels of total and phosphorylated STAT1 and the expression of interferon lambda (IFNL1 and IFLN2) were also downregulated by TM4SF1-AS1 knockdown (Fig. 4E, F). In contrast, overexpression of TM4SF1-AS1 upregulated IFNL genes, total and phosphorylated STAT1 (Tyr 701), and a series of IRDS genes and ISGs in SNU638 cells (Fig. 4G–I and Supplementary Fig. S6C). Notably, Pur-α knockdown abrogated the effects of TM4SF1-AS1 overexpression on STAT1 and ISGs (Fig. 4J, K and Supplementary Fig. S6D). Similarly, knockdown of the RNA sensor RIG-I suppressed IRDS genes in GC cells overexpressing TM4SF1-AS1 (Supplementary Fig. S6E). However, RIP-qPCR assays did not reveal a significant interaction between TM4SF1-AS1 and RIG-I (Supplementary Fig. S7A). Moreover, a cap analysis of gene expression sequencing (CAGE-seq) suggested TM4SF1-AS1 has a 5′ cap structure, making it unlikely that TM4SF1-AS1 is substrate for RIG-I (Supplementary Fig. S7B). These results suggest TM4SF1-AS1 activates interferon signaling in a Pur-α- and RIG-I-dependent manner in GC cells and may also activate RIG-I through an indirect mechanism.

### TM4SF1-AS1 upregulates SGs in GC cells

**Fig. 5:**
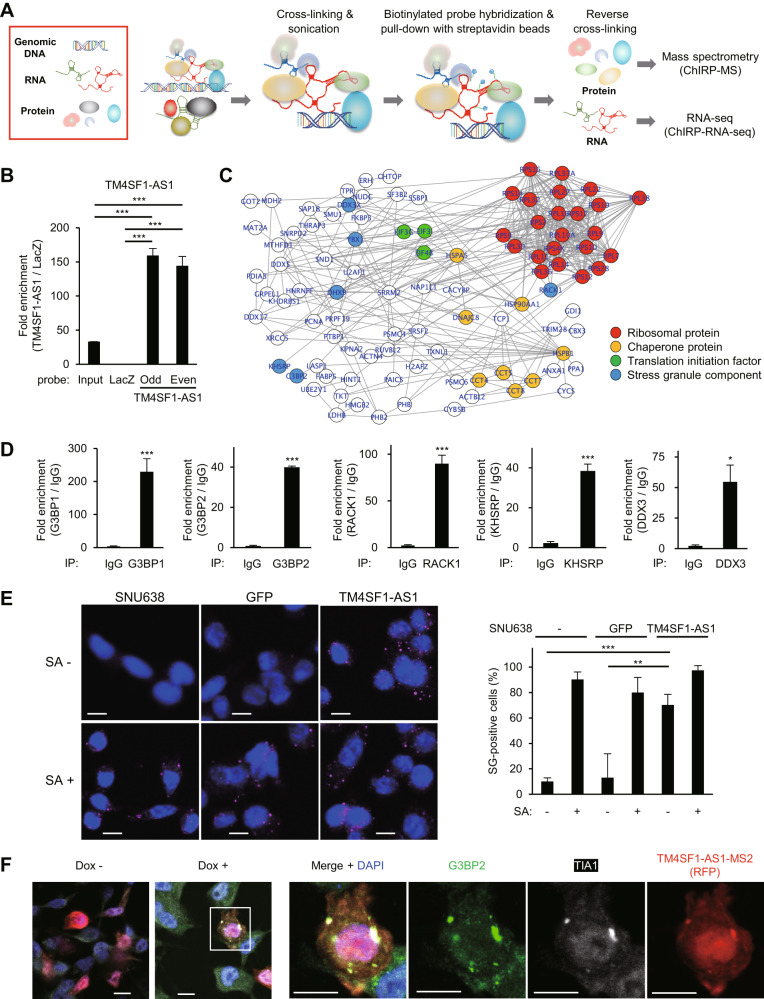
TM4SF1-AS1 promotes stress granule (SG) formation in GC cells. **A** Workflow of ChIRP coupled with mass spectrometry (ChIRP-MS) or RNA-seq (ChIRP-RNA-seq) to identify molecules associated with TM4SF1-AS1. **B** qRT-PCR confirming enrichment of TM4SF1-AS1 in ChIRP products derived from SNU638-TM4SF1-AS1 cells. (*n* = 3). **C** Protein-protein interaction (PPI) network among the proteins identified by ChIRP-MS. Functional categories of the proteins are indicated by node colors. **D** RIP-qPCR assays validating the ChIRP-MS results. The indicated proteins in HSC-45 cells were immunoprecipitated, and co-precipitated TM4SF1-AS1 was detected by qRT-PCR. IgG served as a negative control. (*n* = 3). **E** Immunofluorescence images showing staining of the SG marker G3PB2 in SNU638, SNU638-GFP, and SNU638-TM4SF1-AS1 cells treated with or without sodium arsenite (SA). Summarized results are shown on the right (*n* = 5). Scale bars = 10 μm. **F** Immunofluorescence indicating G3BP2 (green), TIA1 (white), and inducible MS2-tagged TM4SF1-AS1 (red) in SNU638 cells. Cells were transfected with a MS2 coat protein (MCP)-RFP plasmid and incubated for 8 days with or without Dox. Magnified views of the respective markers are shown on the right. Scale bars = 10 μm. *P < 0.05, **P < 0.01, ***P < 0.001.

The above results suggest that TM4SF1-AS1 is functionally associated with Pur-α, YB-1, and RIG-I, all of which are reportedly involved in SGs [24–27]. To further identify molecules associated with endogenous TM4SF1-AS1 in GC cells, we performed Chromatin Isolation by RNA Purification (ChIRP) analysis (Fig. 5A). We confirmed the enrichment of TM4SF1-AS1 among the ChIRP products obtained from SNU638-TM4SF1-AS1 cells by using tiling probes for TM4SF1-AS1 (Fig. 5B). Subsequent proteomic analysis identified 143 proteins that potentially interacted with TM4SF1-AS1 (Supplementary Table S7). GO and network analyses suggested that the proteins associated with RNA binding, translational initiation, and ribosomes were significantly enriched among the identified molecules (Fig. 5C and Supplementary Fig. S8A). In addition, the ChIRP-mass spectrometry (ChIRP-MS) analysis identified several proteins reportedly associated with SGs (Fig. 5C). RIP-qPCR assays confirmed an association between TM4SF1-AS1 and SG-related proteins including G3BP1, G3BP2, RACK1, KHSRP, and DDX3 (Fig. 5D). Immunofluorescent staining using G3BP2 as an SG marker revealed the presence of sodium arsenite (SA)-induced SGs in GC cells (Fig. 5E). We also observed similar foci in SNU638-TM4SF-AS1 cells without SA, suggesting that TM4SF1-AS1 promotes SG formation in GC cells (Fig. 5E). Upregulation of SGs in SNU638-TM4SF1-AS1 cells was further confirmed using a xenograft tumor model (Supplementary Fig. S8B). To determine the cytoplasmic localization of TM4SF1-AS1 in more detail, we established GC cells that inducibly expressed MS2-tagged TM4SF1-AS1 (SNU638-tet-TM4SF1-AS1-MS2 cells). To visualize MS2-tagged TM4SF1-AS1, cells were transfected with the ptagRFP-MS2coatProtein plasmid. We observed colocalization of TM4SF1-AS1, G3BP2, and another SG marker, TIA1, within the foci of GC cells in which TM4SF1-AS1 expression was induced (Fig. 5F). In contrast, MS2 tag alone did not induce SG formation (Supplementary Fig. S8C).

### TM4SF1-AS1 colocalizes with RACK1 in SGs and inhibits apoptosis

**Fig. 6:**
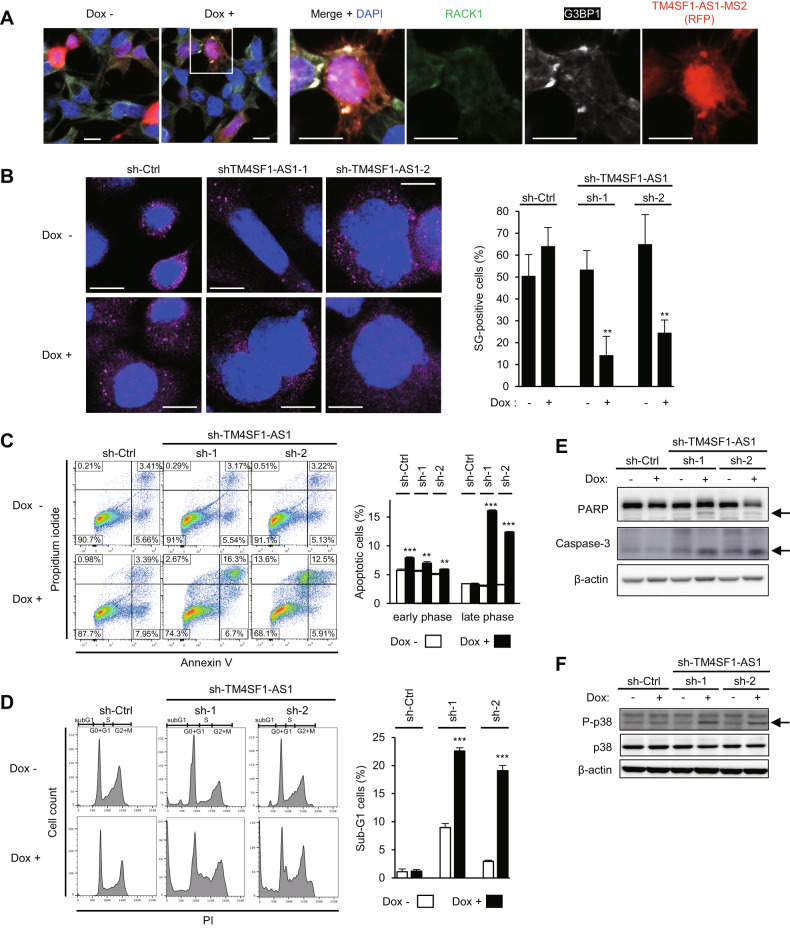
TM4SF1-AS1 inhibits apoptosis by suppressing stress-responsive MAPK signaling in GC cells. **A** Immunofluorescence indicating RACK1 (green), G3BP1 (white), and inducible MS2-tagged TM4SF1-AS1 (red) in SNU638 cells. The cells were transfected with a MS2 coat protein (MCP)-RFP plasmid and incubated for 8 days with or without Dox. Magnified views of the respective markers are shown on the right. Scale bars = 10 μm. **B** Immunofluorescence images of G3PB2 in HSC-45 cells incubated for 8 days with or without Dox and expressing the indicated inducible shRNAs. Representative images are shown on the left. Summarized results are on the right. (*n* = 5). **C**, **D** Apoptosis (**C**) and cell cycle (**D**) assays in HSC-45 cells incubated with or without Dox for 8 days and expressing the indicated inducible shRNAs. Representative results are shown on the left. Summarized results are shown on the right. (*n* = 3). **E**, **F** Western blot analysis of PARP, caspase-3 (**E**) and p38 (**F**) in HSC-45 cells incubated with or without Dox for 8 days and expressing the indicated inducible shRNAs. Cleaved PARP and cleaved caspase-3 are indicated by arrows (**E**). Phosphorylated p38 is indicated by an arrow (**F**). **P < 0.01, ***P < 0.001.

RACK1 is an activator of the stress-responsive MAPK pathway, and previous studies have shown that SG formation inhibits apoptosis by suppressing RACK1 [28]. Therefore, we investigated the involvement of TM4SF1-AS1 in apoptosis of GC cells. Immunofluorescence staining revealed the colocalization of RACK1, G3BP1, and TM4SF1-AS1 in GC cells overexpressing TM4SF1-AS1 (SNU638-tet-TM4SF1-AS1-MS2), suggesting that RACK1 is sequestered within SGs (Fig. 6A). To determine whether TM4SF1-AS1 affected apoptosis, we used GC cells expressing an inducible TM4SF1-AS1 knockdown system (HSC-45-tet-shTM4SF1-AS1). Depletion of TM4SF1-AS1 downregulated SGs (Fig. 6B). Flow cytometric analyses showed that TM4SF1-AS1 knockdown significantly induced apoptosis in GC cells (Fig. 6C), whereas cell cycle analysis revealed substantial increases in sub-G1 populations among TM4SF1-AS1 knockdown cells (Fig. 6D). The levels of cleaved PARP1 and cleaved caspase-3 were also increased in TM4SF1-AS1 knockdown cells (Fig. 6E). In addition, we confirmed induction or suppression of apoptosis in xenograft tumors with knockdown or overexpression of TM4SF1-AS1 (Supplementary Fig. S9). Induction of early apoptosis by TM4SF1-AS1 depletion was attenuated by RACK1 knockdown in GC cells (Supplementary Fig. S10). Moreover, the level of phosphorylated p38 (Thr180/Tyr182) was upregulated by TM4SF1-AS1 knockdown (Fig. 6F). These results suggested that TM4SF1-AS1 inhibits p38-induced apoptosis by sequestering RACK1 within SGs in GC cells.

### TM4SF1-AS1-induced SG upregulation and apoptosis inhibition are dependent on Pur-α and YB-1

**Fig. 7:**
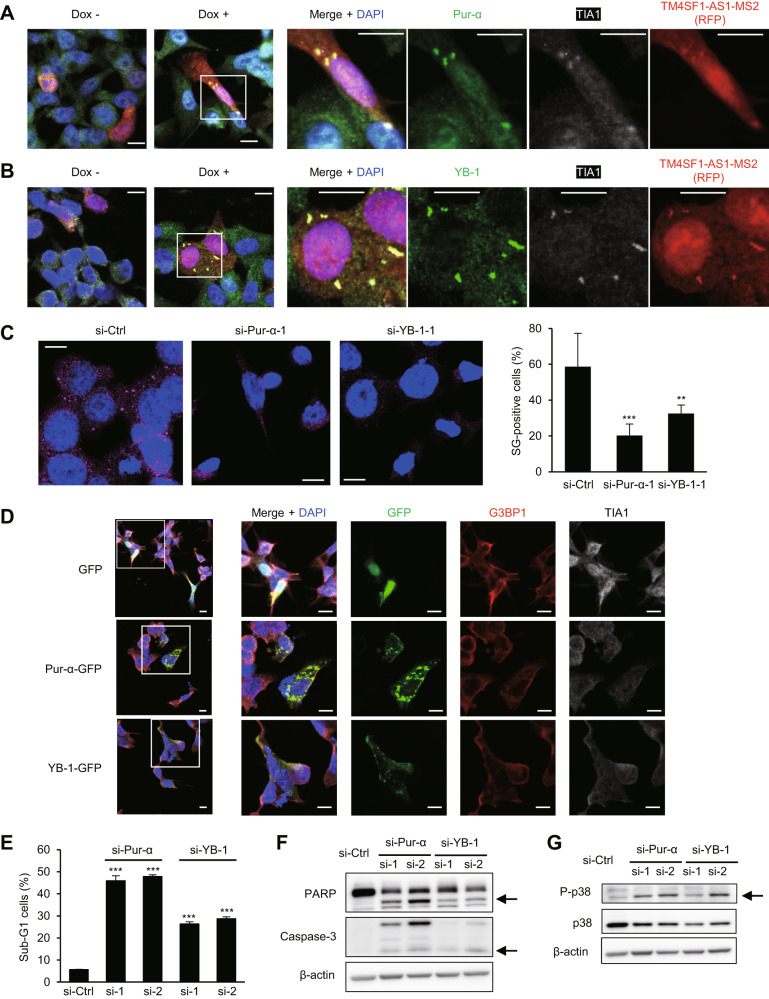
Pur-α and YB-1 contribute to SG formation and apoptosis inhibition in GC cells. **A**, **B** Immunofluorescence images showing Pur-α (A) or YB-1 (B), TIA1 and inducible MS2-tagged TM4SF1-AS1 in SNU638 cells incubated for 8 days with or without Dox. Scale bars = 10 μm. **C** Immunofluorescent staining of G3PB2 in SNU638-TM4SF1-AS1 cells transfected with the indicated siRNAs. Representative results are shown on the left. Summarized results are shown on the right. (*n* = 5). Scale bars = 10 μm. **D** Localization of GFP-tagged Pur-α or YB-1, G3BP1 and TIA1 immunofluorescence in SNU638 cells. Cells were transfected with vectors encoding GFP (upper), GFP-tagged Pur-α (middle) or GFP-tagged YB1 (bottom). Magnified views of the respective markers are shown on the right. Scale bars = 10 μm. **E** Cell cycle analysis of HSC-45 cells expressing the indicated siRNAs. (*n* = 3). **F**, **G** Western blot analysis of PARP and caspase-3 (**F**) and total and phosphorylated p38 (**G**) in HSC-45 cells transfected with the indicated siRNAs. Cleaved PARP, cleaved caspase-3 and phosphorylated p38 are indicated by arrows. **P < 0.01, ***P < 0.001.

**Fig. 8:**
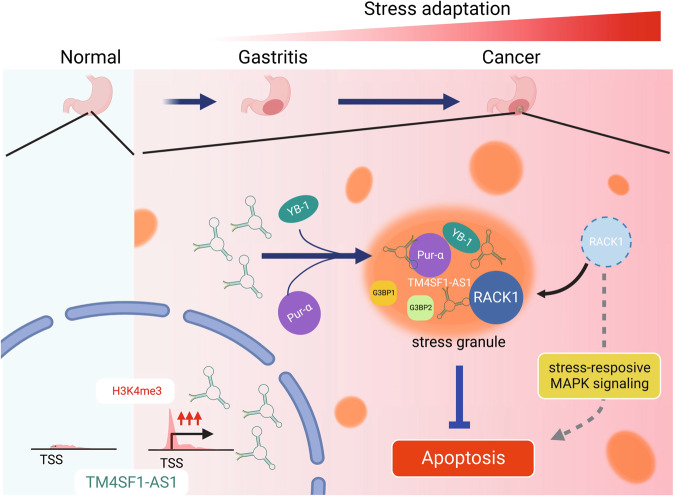
Model for the TM4SF1-AS1-induced stress adaptation. In the early steps of gastric tumorigenesis, TM4SF1-AS1 is transcriptionally activated. TM4SF1-AS1 sequesters RACK1 within SGs, which leads to suppression of stress-responsive MAPK signaling and inhibition of apoptosis (created with BioRender.com).

Next, we used SNU638 cells with inducible TM4SF1-AS1 expression to investigate whether Pur-α and YB-1 contribute to TM4SF1-AS1-induced SG upregulation. We observed that Pur-α and YB-1 colocalize with TIA1 and TM4SF1-AS1 within SGs in cells overexpressing TM4SF1-AS1 (Fig. 7A, B). In addition, Pur-α or YB-1 knockdown significantly downregulated SGs in SNU638-TM4SF1-AS1 cells (Fig. 7C). We then tested whether Pur-α or YB-1 could upregulate SGs and found that ectopically expressed GFP-tagged Pur-α or YB-1 formed foci in SNU638 cells (Fig. 7D). However, they did not colocalize with SG markers, suggesting that they do not form SGs in the absence of TM4SF1-AS1 (Fig. 7D). In contrast, Pur-α or YB-1 knockdown significantly increased the sub-G1 population among HSC-45 cells (Fig. 7E). Cleavage of PARP and caspase-3 and phosphorylation of p38 were also promoted in GC cells after Pur-α or YB-1 knockdown (Fig. 7F, G). These results suggest that although Pur-α or YB-1 alone does not facilitate SG formation, they are required for TM4SF1-AS1-induced SG upregulation and apoptosis inhibition in GC cells (Fig. 8).

Finally, we performed ChIRP-RNA-seq to investigate the RNA molecules that associate with TM4SF1-AS1 in GC cells (Fig. 5A). We identified a series of 585 RNAs, among which TM4SF1-AS1 was the most abundantly detected, confirming successful enrichment of RNA molecules by the ChIRP assay (Supplementary Fig. S11A, B and Supplementary Table S8). Among them, 192 (33%) were transcripts of protein-coding genes, and GO analysis revealed enrichment of genes associated with apoptosis (Supplementary Fig. S11C, D). We selected BCL2L11 (Bim), a well-known apoptosis activator, for further analysis [29]. RNA-seq and qRT-PCR coupled with ChIRP revealed binding of TM4SF1-AS1 to the 3′ untranslated region (3’UTR) of BCL2L11 (Supplementary Fig. S11E, F). RIP-qPCR assays confirmed associations between BLC2L11 mRNA and the TM4SF1-AS1-related proteins Pur-α, YB-1, G3BP1, and G3BP2 (Supplementary Fig. S11G). We also observed decreased expression of BCL2L11 protein in GC cells ectopically expressing TM4SF1-AS1 (Supplementary Fig. S11H). These results suggest that TM4SF1-AS1 may inhibit apoptosis by sequestering mRNAs of apoptosis-related genes in SGs.

## Discussion

Recent studies have shown the upregulation of TM4SF1-AS1 and its oncogenicity in several types of cancer, including GC, hepatocellular carcinoma, and lung cancer, but the underlying molecular mechanism is not fully understood [30–32]. In the present study, we found that TM4SF1-AS1 is transcriptionally activated in the non-tumorous gastric mucosa of GC patients, suggesting its involvement in the early onset of GC development. Our data also demonstrated a novel mechanism by which this lncRNA inhibits apoptosis through SG formation in cancer cells.

We found that TM4SF1-AS1 activated interferon signaling in GC cells. Recent studies have shown that IRDS genes, a subgroup of ISGs, are highly expressed in cancer cells and contribute to tumor growth, migration, invasion, and resistance to chemotherapy and irradiation [33, 34]. We recently reported that one lncRNA, DLEU1, exerts oncogenic effects in oral squamous cell carcinoma through activation of IFITM1, one of the IRDS genes [35]. Taken together, our results suggest that TM4SF1-AS1 may contribute to tumorigenesis by activating interferon signaling. Our finding that TM4SF1-AS1-induced activation of interferon signaling is dependent on RIG-I suggests TM4SF1-AS1 may also activate RIG-I signaling without direct interaction. RIG-I recognizes cytoplasmic 5’-triphosphorylated double-stranded RNAs as non-self RNAs [36]. TM4SF1-AS1 is a 5′-capped lncRNA, which suggests it is unlikely to be a direct target of RIG-I. Further study will be needed to clarify the mechanism underlying TM4SF1-AS1-mediated RIG-I activation.

We found that TM4SF1-AS1 interacts with Pur-α and YB-1, which are multifunctional single-stranded DNA-/RNA-binding proteins [37, 38]. Our results suggest that Pur-α may be involved in TM4SF1-AS1-induced activation of interferon signaling. In recent years, several studies have shown that noncoding RNAs interact with YB-1 to exert oncogenic or tumor-suppressive effects. Additionally, tRNA-derived fragments were shown to bind to YB-1, thereby suppressing breast cancer progression [39]. It was noteworthy to us that both Pur-α and YB-1 are reportedly associated with SGs [24–26].

Our ChIRP-MS analysis revealed that TM4SF1-AS1 is associated with a number of proteins known to be associated with SGs, including ribosomal proteins and translation initiation factors. Although the contribution of SGs to cancer pathogenesis is not fully understood, recent studies have begun to shed light on their functions within tumor cells. For instance, mutant KRAS-dependent SG upregulation confers resistance to stress stimuli [12]. In addition, the assembly of SGs in cancer cells is induced by chemotherapeutic agents, including 5-fluorouracil, bortezomib, and sorafenib, suggesting that SG are involved in chemoresistance [40–42]. Our results also suggest that TM4SF1-AS1 inhibits apoptosis by sequestering RACK1 within the SGs. RACK1 has been reported as a mediator between SG and the stress-activated p38 and JNK MAPK (SAPK) pathway [28]. Under stress caused by X-rays or genotoxic drugs, RACK1 binds to the stress-responsive MAPKKK MTK1 and activates the SAPK pathway, which leads to apoptosis [28]. In contrast, under stress caused by hypoxia or heat shock, RACK1 is sequestered to SGs and the SAPK response is inhibited [28]. Notably, TM4SF1-AS1 promotes SG assembly within cancer cells in the absence of stress, suggesting that TM4SF1-AS1 contributes to tumorigenesis by conferring resistance to stress-induced apoptosis.

Interestingly, TM4SF1-AS1-induced SG formation was dependent on Pur-α and YB-1; however, ectopic expression of Pur-α or YB-1 failed to induce SG assembly in GC cells lacking TM4SF1-AS1 expression. It is also noteworthy that depletion of Pur-α or YB-1 did not affect GC cell viability in the absence of TM4SF1-AS1. These results suggest that TM4SF1-AS1 cooperates with Pur-α and YB-1 to inhibit apoptosis via SG formation in GC cells. We screened for RNAs associating with TM4SF1-AS1 and identified a series of mRNAs of genes involved in apoptosis, including BCL2L11. We also confirmed associations between BCL2L11 mRNA and TM4SF1-AS1-associated proteins, including Pur-α and YB-1. Recently, Pur-α was shown to promote esophageal cancer progression by forming SGs and suppressing translation initiation of IGFBP3 [43]. Taken together, our findings suggest that TM4SF1-AS1 inhibits apoptosis by sequestering RACK1 and mRNAs of apoptosis-related genes in SGs.

An important limitation of this study is that the mechanism underlying TM4SF1-AS1-induced SG formation is not yet fully understood. Recent studies have demonstrated that several lncRNAs trigger liquid-liquid phase separation (LLPS) by acting as scaffolds for RNP granule formation [44]. For instance, NEAT1 recruits the RNA-binding protein NONO via its functional domains and initiates the assembly of the phase-separated paraspeckle structure [45]. A short tandem repeat (STR)-enriched RNA, PNCTR, sequesters pyrimidine tract-binding protein 1 (PTBP1) within a nuclear body called the perinucleolar compartment and inhibits apoptosis by modulating the splicing regulation function of PTBP1 [46]. NEAT1 and PNCTR are architectural RNAs (arcRNAs), which function as scaffolds or platforms for nuclear bodies and are characterized by multivalent interactions with RBPs [44]. However, it is unlikely that TM4SF1-AS1 has such multivalencies because of the lack of apparent RBP-binding elements, and TM4SF1-AS1 may not act as a scaffold for SG formation. Transcriptome analysis of purified SGs revealed that mRNA accumulation within SGs correlates with longer length and poor translatability of mRNAs [47]. In addition, emerging evidence suggests that RNA-RNA interactions can contribute to RNP granule formation, while the interaction is limited by RBPs, helicases, and ribosomes, which enables normal RNA function [7]. However, because of its comparatively short length and lack of multivalency, the RNA-RNA interaction model may not be applicable to TM4SF1-AS1-induced SG formation. Alternatively, an excess of cytoplasmic TM4SF1-AS1 may facilitate stochastic interactions with G3BP proteins and activate a core protein-RNA network that drives SG assembly [48, 49]. In addition, TM4SF1-AS1 may interact with other SG components, including TIA1, which mediates SG formation [50]. SG assembly is preceded by the inactivation of translation initiation [9]. Interactions between TM4SF1-AS1 and translation initiation factors suggest that the inhibition of translation initiation by TM4SF1-AS1 may trigger SG assembly.

In conclusion, we identified epigenetic activation of TM4SF1-AS1 during the early onset of gastric tumorigenesis. We found that TM4SF1-AS1 may be associated with innate immunity, and that it inhibits apoptosis through SG formation in cancer cells. Stress responses are defense mechanisms by which cells overcome their vulnerability to adverse environments. Cancer cells may acquire stress resistance by sequestering potentially cytotoxic or pro-apoptotic molecules within the SGs. Our results indicate that TM4SF1-AS1 may contribute to tumorigenesis by enhancing SG-mediated stress adaptation. Thus, TM4SF1-AS1-induced SG formation might be an effective target for cancer prevention and treatment.

## Supplementary information

Supplementary Methods

Supplementary Tables

Supplementary Figures

Original images of western blots

## Data Availability

The Gene Expression Omnibus accession number for the microarray data is GSE209814. Targeted sequencing data were deposited at the National Bioscience Database Center (NBDC; accession ID, JGAS000353). Proteomic data were deposited in jPOST (accession ID, JPST001842; ProteomeX change accession ID, PXD036766). ChIRP-RNA-seq data were deposited in NCBI SRA (accession ID, PRJNA943468).
