# Supplementary material for: TM4SF1-AS1 inhibits apoptosis by promoting stress granule formation in cancer cells
Source: Cell Death Dis. 2023 Jul 13;14(7):424. doi: 10.1038/s41419-023-05953-3 (PMC10345132; doi:10.1038/s41419-023-05953-3)
Supplement: Supplementary file 1 — Supplementary Methods [file 41419_2023_5953_MOESM1_ESM.docx]

**Supplementary Methods**

**Transfection of plasmids and siRNAs**

Plasmid vectors were transfected using Lipofectamine 3000 (Thermo Fisher Scientific, Waltham, MA). To establish GC cells with or without stable TM4SF1-AS1 expression, SNU638 cells were transfected with pBI-CMV2-NeoR-GFP-TM4SF1-AS1 or pBI-CMV2-NeoR-GFP and then selected in culture medium containing 500 μg/ml neomycin (G418) for 2 weeks, after which GFP-positive cells were sorted using BD FACS Aria II (BD Biosciences, Franklin Lakes, NJ, USA). siRNAs targeting TM4SF1, TM4SF1‑AS1, purine-rich element-binding protein α (Pur-α) or Y-box binding protein 1 (YB-1) were purchased from Sigma-Aldrich (St. Louis, MO, USA). Silencer Select siRNA targeting siDDX58 (s223614) was purchased from Thermo Fisher Scientific. siRNAs targeting other genes were designed using the siDirect algorithm (http://sidirect2.rnai.jp/) and manufactured by RNAi (Tokyo, Japan). The siRNAs were transfected using Lipofectamine RNAiMax (Thermo Fisher Scientific). The siRNA sequences are listed in Supplementary Table S9.

**Inducible knockdown or expression of TM4SF1-AS1**

Lentiviral particles expressing small hairpin RNA (shRNA) or MS2-tagged TM4SF1-AS1 were produced using a Lenti‑PacTM HIV Expression Packaging Kit (GeneCopoeia, Rockville, MD, USA) according to the manufacturer’s instructions. After infection, cells were selected in a culture medium containing 1 μg/mL puromycin for a week. Cells transfected with the Tet-on inducible system were cultured in RPMI1640 supplemented with 10% Tet-approved fetal bovine serum (Takara Bio Inc.). shRNA or TM4SF1-AS1 expression was induced by culturing the cells in the presence of 2.5 μg/ml doxycycline (Dox, Sigma-Aldrich). The shRNA sequences are listed in Supplementary Table S9.

**Cell viability and colony formation assay**

For cell viability assays, 2000 to 5000 cells per well in 96-well plates were transfected with siRNAs as described above. Cell viabilities were then measured using a Cell Counting kit-8 (Dojindo, Kumamoto, Japan). For colony formation assays, cells transfected with inducible shRNA vectors (10,000 cells per well in 6-well plates) were cultured with or without 2 μg/ml DOX for 8 days. Colonies were then stained with Giemsa and measured using ImageJ software (NIH, Bethesda, MD, USA).

**Cell migration and invasion assays**

Cell migration and invasion assays were performed using transwell chambers as described previously [[1](#_ENREF_1)]. Briefly, cells were transfected with siRNA as described above and incubated for 24 h, after which 5×10^4^ cells were added to the upper chamber. RPMI1640 medium supplemented with 10% fetal bovine serum was added to the lower chamber. After incubation for 22 h, migrating or invading cells were stained using a Diff quick stain kit (Sysmex, Tokyo, Japan).

**Mutation analysis**

A custom panel was designed for targeted sequencing of lncRNA- and GC-related genes using Ion AmpliSeq designer (Thermo Fisher Scientific; Supplementary Table S10). Libraries were prepared using the Ion AmpliSeq custom Panel and the Ion AmpliSeq Library Kit 2.0 (Thermo Fisher Scientific). Sequencing was carried out using an Ion Proton System (Thermo Fisher Scientific). The data were analyzed using Torrent Suite v.5.0.4 software (Thermo Fisher Scientific), and the variant call was performed using VarScan2. Targeted sequencing data are available in the National Bioscience Database Centre (NBDC) under the dataset accession ID JGAS000353.

**Flow cytometry analysis**

Cells were transfected with siRNA as described above and incubated for 96 h. HSC-45 cells with inducible shRNAs were incubated for 8 days with or without 2 μg/ml Dox. Apoptosis was analyzed using an ApoScreen Annexin V Apoptosis Kit (SouthernBiotech, Birmingham, AL, USA). Cells were stained with annexin V-FITC and propidium iodide (PI) according to the manufacturer’s manual. To analyze cell cycle, the cells were stained with PI. Cells were subjected to FACS analysis using a BD FACSCanto II (BD Biosciences) with BD FACSDiva software (BD Biosciences). Data were analyzed using FlowJo software version 10 (FlowJo LLC, Ashland, OR, USA).

**Gene expression microarray analysis**

Cells were transfected with siRNAs as described above and incubated for 72 h. Gene expression microarray analysis was performed using a SurePrint G3 Human GE 8x60K v2 microarray (Agilent Technologies, Santa Clara, CA, USA) as described previously [[1](#_ENREF_1)]. Data were analyzed using GeneSpring GX version 13 (Agilent Technologies). GO analysis was performed using DAVID (https://david.ncifcrf.gov/). GSEA was performed using gene sets that included hallmark gene sets, ISGs and IRDS [[2](#_ENREF_2), [3](#_ENREF_3)]. The Gene Expression Omnibus accession number for the microarray data is GSE209814.

**In vitro transcription**

DNA templates for TM4SF1-AS1 and its antisense for in vitro transcription were PCR amplified from a plasmid vector. RNAs labeled with 5-Bromo-UTP (BrU) or biotin were synthesized using a CUGA in vitro Transcription Kit ver. 7 (NIPPON GENETEC, Tokyo, Japan). Briefly, 1.25 pmol of template DNA were added to a reaction mixture containing T7 polymerase and BrU mix (3.75 mM BrU and 3.75 mM UTP) or biotin-UTP mix (1.5 mM biotin-UTP and 3.5 mM UTP). RNA products were heated in structure buffer (10 mM Tris-HCl [pH 7.0], 0.1 M KCl, 10 mM MgCl_2_ and 40 U RNaseOUT (Thermo Fisher Scientific)) for 2 min at 90°C, followed by incubation on ice for 2 min and at room temperature for 20 min. The primer sequences are listed in Supplementary Table S1.

**RNA pull-down and mass spectrometry**

RNA pull-down assays were performed using a RiboCluster Profiler RiboTrap Kit (MBL, Tokyo, Japan). Briefly, BrU or biotin-labeled RNAs (100 pmol) were mixed with cellular extracts, after which RNA-protein complexes were immunoprecipitated with BrdU antibody-immobilized Protein G Dynabeads (Thermo Fisher Scientific) or pulled-down with Dynabeads MyOne streptavidin T1 beads (Thermo Fisher Scientific). Precipitates were separated with SDS-PAGE and stained using GelCode Blue Stain Reagent (Thermo Fisher Scientific).

Bands specific for TM4SF1-AS1, a negative control or mock were excised and subjected to in‑gel trypsin digestion. The gel containing proteins was reduced with 10 mM dithiothreitol for 60 min at 56°C, followed by alkylation with 54 mM iodoacetamide in the dark at room temperature for 45 min. In-gel digestion was executed using trypsin overnight at 37°C, as described previously [[4](#_ENREF_4)].

Liquid chromatography and peptide spotting were performed using direct nano-LC and a MALDI fraction system DiNa-MaP (KYA Technology, Tokyo, Japan) with partially modified methods, as described previously [[5](#_ENREF_5), [6](#_ENREF_6)]. Elution solvent A was 0.1% trifluoroacetic acid (TFA), and solvent B was 0.1% TFA in 70% acetonitrile (ACN). The peptides were separated on a HiQsilC18W‑3 column (100 μm ID×100 mm; KYA Technology) with a gradient program (0-12 min, 0-5% solvent B; 12-20 min, 5-50% solvent B; 20-25 min, 50-100% solvent B; 25-30 min, 100-5% solvent B; 30-40 min, 5% solvent B) at a flow rate of 300 nL/min. Separated peptides were mixed with matrix solution (4 mg/mL α-cyano-4-hydroxycinnamic acid, 70% ACN, 0.1% TFA, 80 μg/mL diammonium hydrogen citrate) and spotted onto a 384-well AB OptiTOF MALDI Plate Insert (AB SCIEX, Framingham, MA). Mass spectrometry was performed using a 4800 Plus MALDI-TOF/TOF Analyzer (AB Sciex) with 4000 Series Explorer version 3.5 software (AB Sciex). Mass accuracy was calibrated using a 6-peptide mixture (AB Sciex). MS spectra were obtained in positive ion mode from m/z 800 to 4000 and accumulated from 1000 laser shots in a randomized raster. MS/MS spectra with S/N ≥100 were acquired using air as the collision gas with a collision energy of 1 kV and the following parameters: acquisition order/fraction, strongest precursors first; maximum precursors/fraction, 10. MS/MS data were searched against the human International Protein Index (IPI) database version 3.63 (European Bioinformatics Institute, Cambridgeshire, UK) using Protein Pilot version 2.0 (AB Sciex) equipped with the Paragon search algorithm [[7](#_ENREF_7)]. Proteins with an unused ProtScore > 1.3 (corresponding to 95% confidence) were selected.

**RNA immunoprecipitation**

RNA immunoprecipitation (RIP) was performed using modified methods, as described previously [[8](#_ENREF_8)]. HSC-45 cells (3×10^7^) were resuspended in 400 μl of ice-cold PBS and incubated for 20 min on ice, after which 400 μl of nuclear isolation buffer (1.28 M sucrose, 40 mM Tris-HCl [pH 7.4], 20 mM MgCl2, 4% Triton X-100) were added to the cells and incubated for 20 min on ice. Thereafter, 1.2 ml of cold Mili Q water with 200 U/ml RNaseOUT were added to the resuspended cells and incubated for 20 min on ice. The resuspension was centrifuged at 2,500×g for 15 min at 4°C and then resuspended in 2 ml of RIP buffer (150 mM KCl, 25 mM Tris-HCl [pH 7.4], 5 mM EDTA, 0.5 mM DTT, 50% NP40, 200 U/mL RNaseOUT, a protease inhibitor cocktail) and crushed using a dounce homogenizer. The lysate was centrifuged at 13,000 rpm for 10 min at 4°C. Ten μl of the supernatant were stocked as an input sample, and the remaining portion was subjected to immunoprecipitation. Aliquots of the lysate were immunoprecipitated using rabbit anti-Pur-α pAb (ab125200, Abcam, Cambridge, UK), rabbit anti-YB-1 pAb (RN015P, MBL), rabbit anti-RIG-I mAb (#3743, Cell Signaling Technology, Danvers, MA, USA), rabbit anti-G3BP1 pAb (13057-2-AP, Proteintech, Rosemont, IL, USA), rabbit anti-G3BP2 pAb (16276-1-AP, Proteintech), rabbit anti‑RACK1 pAb (A302-545A-1, Bethyl laboratories, Waltham, MA, USA), rabbit anti-KHSRP mAb (ab150393, Abcam), rabbit anti-DDX3X pAb (11115-1-AP, Proteintech) or anti-rabbit IgG antibody (#3900S, Cell Signaling Technology, Danvers, MA, USA). Immunoprecipitants were incubated with protein G Dynabeads for 2 h at 4°C. The beads were then washed three times in RIP buffer. RNA was extracted from the beads as described above. Co-precipitating TM4SF1-AS1 was detected using qRT‑PCR.

**Chromatin isolation by RNA purification, mass spectrometry and RNA-seq**

Chromatin Isolation by RNA Purification (ChIRP) was performed using a modified method as described previously [[9-11](#_ENREF_9)]. Probes for TM4SF1-AS1 and LacZ were designed using the ChIRP probe designer (https://www.biosearchtech.com/support/tools/design-software/chirp-probe-designer). For ChIRP-mass spectrometry, the seven synthesized probes were divided into odd-numbered (#1, #3, #5, #7) and even-numbered (#2, #4, #6) sets. For ChIRP-RNA-seq, a pool of the seven probes was used. Sequences of the probes are listed in Supplementary Table S11. SNU638-TM4SF1-AS1 cells (3×10^7^ cells for each probe) were crosslinked with 3% formaldehyde for 30 min at room temperature, then quenched with 1.25 M glycine for 5 min. Crosslinked cells were pelleted by centrifugation at 2000 g and rinsed twice with ice-cold PBS. The cells were then suspended in lysis buffer (50 mM Tris-HCl [pH 7.0], 10 mM EDTA, 1% SDS, 1 mM AEBSF, protease inhibitor cocktail (Roche Diagnostics, Mannheim, Germany), 200 U/ml RNaseOUT) and incubated for 10 min on ice. The resultant cell lysates were sonicated using a Covaris S2 system (Covaris, Inc.) at the following settings: Duty cycle 5%, Intensity 2, cycle per burst 200, time 30 min, and bath temperature 4°C. The lysates were centrifuged at 15,000 rpm for 10 min at 4°C. Ten μl of the supernatant were stocked as an input sample, and the remaining portion was subjected to ChIRP assays. The lysate was diluted three times with hybridization buffer (750 mM NaCl, 50 mM Tris-HCl [pH 7.0], 1 mM EDTA, 1% SDS, 1 mM AEBSF, protease inhibitor cocktail, 200 U/ml RNaseOUT), and each 100 pmol probe set was added to three aliquots of the diluted lysates. The lysates were then incubated with rotation for 4 h at 37°C. A 1:1 mixture of Dynabeads MyOne streptavidin beads C1 (Cat no. 65001, Thermo Fisher Scientific) and T1 (Cat no. 65601, Thermo Fisher Scientific) was added to the lysates, which were then incubated with rotation for 30 min at 37°C. The beads were washed four times with rotation in a wash buffer (2×SSC, 0.5% SDS, 1 1M AEBSF) for 5 min at 37°C. For elution by reverse crosslinking, the beads were resuspended with 8 M guanidine-HCl and incubated for 16 h at 65°C, then incubated for 3 h at 95°C.

To prepare peptides for mass spectrometry, the eluate was diluted with 0.2 M ammonium bicarbonate to reduce the concentration of guanidine-HCl buffer (final concentration of 6 M) and was concentrated using an Amicon Ultra-0.5 (Merck). Subsequent sample preparation (alkylation, trypsin digestion and desalting) were performed as described previously, with some modification [[6](#_ENREF_6)]. Briefly, samples were reduced with 10 mM dithiothreitol and alkylated with 20 mM iodoacetamide. For tryptic digestion, samples were diluted (1:4) with 50 mM ammonium bicarbonate to further reduce the concentration of guanidine-HCl buffer (final concentration of 1.5 mol/L) and digested with trypsin overnight. After digestion, the mixture was acidified with TFA (final concentration of 0.1%). To remove interfering substances before mass spectrometry, the mixture was desalted with MonoSpin C18 (GL Sciences, Tokyo, Japan) according to the manufacturer’s instructions. Proteome analysis was performed using an Orbitrap mass spectrometer (Q-Exactive Plus, Thermo Fisher Scientific) as described previously [[12](#_ENREF_12)]. Proteomic data were deposited in jPOST (accession ID, JPST001842; ProteomeXchange accession ID, PXD036766).

RNAs isolated by ChIRP were reverse-transcribed using a GenNext RamDA-seq Single Cell Kit (TOYOBO, Osaka, Japan) according to the manufacturer’s instructions. Sequencing libraries were prepared from the synthesized cDNA using a Nextera XT DNA Library Preparation Kit (Illumina, San Diego, CA, USA) and sequenced using NextSeq 550 (Illumina). RNA-seq data were analyzed using the STAR-RSEM pipeline, and the results were visualized using Integrated Genomic Viewer (IGV) software (Broad Institute, Boston, MA, USA). The NCBI SRA accession number for the RNA-seq data is PRJNA943468.

**In vitro RNA pull-down assays**

HEK293 cells were transfected with pCMV6 Pur-α-myc or pCMV6 YB-1-myc vector and incubated for 48 h, after which they were lysed in 500 μl of M-PER Mammalian Protein Extraction Reagent (Thermo Fisher Scientific) containing 1 mM AEBSF and a protease inhibitor cocktail, then incubated for 10 min at 4°C. The lysates were centrifuged at 14,000×g for 10 min at 4°C, and Myc-tagged Pur-α and YB-1 were purified from the supernantnt using c-Myc-tagged Protein Mild Purification Kit version 2 (MBL). The concentrations of purified Myc-tagged Pur-α and YB-1 proteins were determined using a Bradford microassay (Bio-Rad, Hercules, CA, USA). Two hundred ng of Myc‑tagged Pur-α or YB-1 were incubated with 5 pmol of biotinylated RNAs in 300 μl of binding buffer (50 mM Tris-HCl [pH 8.0], 100 mM KCl, 5 mM MgCl2, 0.1% NP40, 5% glycerol, β‑mercaptoethanol, 200 U/mL RNaseOUT, 1 mM AEBSF and a protease inhibitor cocktail) for 2 h at 4°C with agitation. Dynabeads MyOne streptavidin T1 beads (Thermo Fisher Scientific) were then added to samples and incubated with rotation for 1 h at 4°C, after which the beads were washed three times with NT2 buffer (50 mM Tris-HCl [pH 7.4], 150 mM NaCl, 1 mM MgCl2, 0.05% NP40). The beads were then incubated with 20 μl SDS sample buffer (122.5 mM Tris-HCl [pH 6.8], 2% SDS, 10% glycerol, 0.004% bromophenol blue, 1% 2-mercaptoethanol) for 5 min at 95°C. After removal of the beads, supernatants were subjected to western blot analysis.

**Western blot analysis**

Western blot analysis was performed as described previously [[1](#_ENREF_1)]. A rabbit anti-RBMX mAb (1:1000 dilution, #14794, Cell Signaling Technology), rabbit anti-Pur-α pAb (1 μg/mL, ab79936, Abcam), rabbit anti-Pur-β pAb (1:500 dilution, ab111112, Abcam), rabbit anti-YB-1 mAb (1:1000 dilution, #9744, Cell Signaling Technology), rabbit anti-Phospho-Stat1 (Tyr701) mAb (1:1000 dilution, #9167, Cell Signaling Technology), rabbit anti-Stat1 mAb (1:1000 dilution, #14994, Cell Signaling Technology), rabbit anti-G3BP1 pAb (1:1000 dilution, 13057-2-AP, Proteintech), rabbit anti-G3BP2 pAb (1:1000 dilution, 16276-1-AP, Proteintech), rabbit anti‑RACK1 pAb (1:2000, A302-545A-1, Bethyl laboratories, Waltham, MA, USA), Anti-Myc-tag mAb (1:1000, MBL), rabbit anti-PARP pAb (1:1000 dilution, GTX100573, GeneTex), rabbit anti-Cleaved Caspase-3 mAb (1:1000 dilution, #9664, Cell Signaling Technology), rabbit anti-p38 MAPK (phospho Thr180/Tyr182) pAb (1:1000 dilution, GTX133460, GeneTex) and rabbit anti-p38 pAb (1:1000 dilution, GTX110720, GeneTex) were used. An anti‑mouse IgG antibody (1:5000 dilution, #7076, Cell Signaling Technology) or anti-rabbit IgG antibody conjugated with horseradish peroxidase (1:5000 dilution, #7074, Cell Signaling Technology) was used as the secondary antibody. Proteins were reacted with Clarity Western ECL substrate (Bio-Rad, Hercules, CA, USA) and were detected using ImageQuant LAS4000 mini (GE Healthcare Japan, Hino, Japan).

**Co-immunoprecipitation**

HSC-45 cells were lysed in 700 μl of lysis buffer (500 mM Tris-HCl [pH 7.4], 2% NP40, 150 mM NaCl, 200 U/ml RNaseOUT and a protease inhibitor cocktail) and then incubated for 30 min on ice. After centrifugation, the supernatants were incubated with Protein G Dynabeads for 10 min at 4°C. After removing the beads, the supernatants were incubated with 10 μg of rabbit anti-Pur-α pAb (ab125200, Abcam), 7.5 μg of rabbit anti-YB-1 pAb (RN015P, MBL) or mock for 2 h at 4°C. This was followed by incubation with Protein G Dynabeads for 2 h at 4°C. Alternatively, the precipitated proteins were treated with 10 μg/ml RNaseA (NIPPON GENE, Tokyo, Japan) for 30 min in 37°C. The beads were then washed three times with ice-cold wash buffer (500 mM Tris-HCl [pH 7.4], 2% NP40, 150 mM NaCl) and incubated with 20 μl SDS sample buffer for 5 min at 95°C, after which the supernatants were subjected to western blot analysis.

**Immunofluorescent staining**

Cells were treated for 1 h with or without 500 μM sodium arsenite. Cells or cryosectioned xenograft tumors were fixed with 4% formaldehyde, permeabilized with 0.2% Triton X-100 in PBS, and then incubated in blocking buffer (0.3 M glycine, 1% BSA in PBS) for 30 min. After blocking, the cells were incubated with a rabbit anti-G3BP2 pAb (1:200 dilution, 16276-1-AP, Proteintech), mouse anti-TIA-1 mAb (1:100 dilution, sc166247, Santa Cruz Biotechnology, Santa Cruz, CA, USA), rabbit anti-G3BP1 pAb (1:200 dilution, 13057-2-AP, Proteintech), mouse anti-RACK1 mAb (1:200 dilution, #610177, BD Biosciences), rabbit anti-Pur-α pAb (5 μg/mL, ab79936, Abcam) or rabbit anti-YB-1 pAb (1:100, #4202, Cell Signaling Technology) for 30 min at room temperature. This was followed by incubation with goat anti-rabbit Alexa Fluor 488 (1:500 dilution, A11008, Thermo Fisher Scientific), goat anti-rabbit Alexa Fluor 555 (1:500 dilution, A21428, Thermo Fisher Scientific), goat anti-rabbit Alexa Fluor 647 (1:500 dilution, A21244, Thermo Fisher Scientific), goat anti-mouse Alexa Fluor 488 (1:500 dilution, A11001, Thermo Fisher Scientific) or goat anti-mouse Alexa Fluor 647 (1:500 dilution, A21235, Thermo Fisher Scientific) for 30 min at room temperature. The cells were then mounted with Prolong Glass Antifade Mountant with NucBlue (Thermo Fisher Scientific). Images were acquired using a Zeiss LSM780 confocal microscope system (Carl Zeiss AG, Oberkochen, Germany).

**Data analysis**

RNA sequencing (RNA-seq) data for primary GCs in TCGA datasets were obtained from UCSC Xena (<http://xena.ucsc.edu/>). Cap analysis of gene expression-sequencing (CAGE-seq) data were obtained from the functional annotation of the mammalian genome (FANTOM, https://fantom.gsc.riken.jp/). The protein-protein interaction (PPI) network was reconstructed using the BIND, BioGRID and HPRD databases and was visualized using Cytoscape version 3.9.1 (Institute for Systems Biology, Seattle, WA, USA) [[13-16](#_ENREF_13)].

**References**

1. Nishiyama K, Maruyama R, Niinuma T, Kai M, Kitajima H, Toyota M*, et al.* Screening for long noncoding RNAs associated with oral squamous cell carcinoma reveals the potentially oncogenic actions of DLEU1. Cell Death Dis. 2018;9:826.

2. Hubel P, Urban C, Bergant V, Schneider WM, Knauer B, Stukalov A*, et al.* A protein-interaction network of interferon-stimulated genes extends the innate immune system landscape. Nat Immunol. 2019;20:493-502.

3. Weichselbaum RR, Ishwaran H, Yoon T, Nuyten DS, Baker SW, Khodarev N*, et al.* An interferon-related gene signature for DNA damage resistance is a predictive marker for chemotherapy and radiation for breast cancer. Proc Natl Acad Sci U S A. 2008;105:18490-18495.

4. Shevchenko A, Wilm M, Vorm O, Mann M. Mass spectrometric sequencing of proteins silver-stained polyacrylamide gels. Anal Chem. 1996;68:850-858.

5. Oki G, Wada T, Iba K, Aiki H, Sasaki K, Imai SI*, et al.* Metallothionein deficiency in the injured peripheral nerves of complex regional pain syndrome as revealed by proteomics. Pain. 2012;153:532-539.

6. Aoyama T, Takasawa A, Takasawa K, Ono Y, Emori M, Murata M*, et al.* Identification of Coiled-Coil Domain-Containing Protein 180 and Leucine-Rich Repeat-Containing Protein 4 as Potential Immunohistochemical Markers for Liposarcoma Based on Proteomic Analysis Using Formalin-Fixed, Paraffin-Embedded Tissue. Am J Pathol. 2019;189:1015-1028.

7. Shilov IV, Seymour SL, Patel AA, Loboda A, Tang WH, Keating SP*, et al.* The Paragon Algorithm, a next generation search engine that uses sequence temperature values and feature probabilities to identify peptides from tandem mass spectra. Mol Cell Proteomics. 2007;6:1638-1655.

8. Rinn JL, Kertesz M, Wang JK, Squazzo SL, Xu X, Brugmann SA*, et al.* Functional demarcation of active and silent chromatin domains in human HOX loci by noncoding RNAs. Cell. 2007;129:1311-1323.

9. Chu C, Qu K, Zhong FL, Artandi SE, Chang HY. Genomic maps of long noncoding RNA occupancy reveal principles of RNA-chromatin interactions. Mol Cell. 2011;44:667-678.

10. Chu C, Quinn J, Chang HY. Chromatin isolation by RNA purification (ChIRP). J Vis Exp. 2012.

11. Chu C, Zhang QC, da Rocha ST, Flynn RA, Bharadwaj M, Calabrese JM*, et al.* Systematic discovery of Xist RNA binding proteins. Cell. 2015;161:404-416.

12. Saito Y, Takasawa A, Takasawa K, Aoyama T, Akimoto T, Ota M*, et al.* Aldolase A promotes epithelial-mesenchymal transition to increase malignant potentials of cervical adenocarcinoma. Cancer Sci. 2020;111:3071-3081.

13. Shannon P, Markiel A, Ozier O, Baliga NS, Wang JT, Ramage D*, et al.* Cytoscape: a software environment for integrated models of biomolecular interaction networks. Genome Res. 2003;13:2498-2504.

14. Bader GD, Betel D, Hogue CW. BIND: the Biomolecular Interaction Network Database. Nucleic Acids Res. 2003;31:248-250.

15. Stark C, Breitkreutz BJ, Reguly T, Boucher L, Breitkreutz A, Tyers M. BioGRID: a general repository for interaction datasets. Nucleic Acids Res. 2006;34:D535-539.

16. Peri S, Navarro JD, Amanchy R, Kristiansen TZ, Jonnalagadda CK, Surendranath V*, et al.* Development of human protein reference database as an initial platform for approaching systems biology in humans. Genome Res. 2003;13:2363-2371.
