# Supplementary material for: TM4SF1-AS1 inhibits apoptosis by promoting stress granule formation in cancer cells
Source: Cell Death Dis. 2023 Jul 13;14(7):424. doi: 10.1038/s41419-023-05953-3 (PMC10345132; doi:10.1038/s41419-023-05953-3)
Supplement: Supplementary file 2 — Supplementary Tables [file 41419_2023_5953_MOESM2_ESM.pdf]

Supplementary Table S1. Sequences of the primers used in this study

qRT-PCR		
TM4SF1-AS1	Forward	5'-AACAGAGGTGGCATCAGTCAA-3'
	Reverse	5'-TGGCATCTTCAACTCCCAACT-3'
TM4SF1	Forward	5'-ATGCCTCCGAAAACCACCTC-3'
	Reverse	5'-GTCATCCTGTTCCAGCCCAA-3'
TCONS_9689	Forward	5'-TCAGCAGAAAAGGGAGGACA-3'
	Reverse	5'-TCCAGCACAAGTTTACATCAGT-3'
TCONS_11229	Forward	5'-TGATTTTGGCGATGGTTTCAC-3'
	Reverse	5'-GGCAGGATTACTTCACAGCA-3'
TCONS_21627	Forward	5'-CCGAAGACTTGCAGGGTAAGAA-3'
	Reverse	5'-CTGTCCCCATGCCCCAAAG-3'
TCONS_4302	Forward	5'-AAACTGCCGCGATGAATGTC-3'
	Reverse	5'-TGGAGGGACAGAGTTCTCAGA-3'
TCONS_21520	Forward	5'-TTCATTTACATGGGGTGAGCA-3'
	Reverse	5'-TTGAGTGCAAGTTGTCCAGA-3'
TCONS_9370	Forward	5'-GCCGAACTGCAATCAAGAGT-3'
	Reverse	5'-ATGTCCTTGAGAATCCACTGG-3'
TCONS_24597	Forward	5'-CCGGTCCCCAGATTCTTGAC-3'
	Reverse	5'-CCCGTGTCTGATCCCTTTCC-3'
TCONS_250	Forward	5'-CCTTGTGCTCTCAGAACTCCA-3'
	Reverse	5'-TCCCTGCACTGTGGTCAAAG-3'
TCONS_24194	Forward	5'-CCGACATCAGTGAGAAGGAGG-3'
	Reverse	5'-GAGCAGGGCATGGAGAGAC-3'
TCONS_280	Forward	5'-TGAGTGAAGAAAGAGAGGGGAC-3'
	Reverse	5'-CATCTTGTCACATGCTGCCA-3'
TCONS_14492	Forward	5'-AGAGTGAGGATATCTTGGAGCC-3'
	Reverse	5'-TCTGTAGTCTGTCCGCTTGG-3'
TCONS_1775	Forward	5'-CGCAGTGGATCATGATGGAA-3'
	Reverse	5'-CCTACAGGCTCACATTCTTCA-3'
TCONS_15742	Forward	5'-TCGGGGTCCTGCATTTAGTG-3'
	Reverse	5'-ATGCCGTTCTCCTTGCAGAA-3'
TCONS_19352	Forward	5'-GCAGACATGGGATAACTTTAAGG-3'
	Reverse	5'-TTTATACCAGCCTGCCAATGT-3'
PURA	Forward	5'-ACATCCAGAACAAGCGCTTC-3'
	Reverse	5'-GGAGAGAGTAAGGCGGCTC-3'
YBX1	Forward	5'-GGGTGCAGGAGAACAAGGTA-3'
	Reverse	5'-TTAGGGTTTTCTGGGCGTCT-3'
RACK1	Forward	TCTCCTCAGATGGCCAGTTT
	Reverse	GCTCTCATCCTGGACAGTGT
BST2	Forward	5'-GCAACAAGAGCTGACCGAG-3'
	Reverse	5'-AGCTCCTCCACTTTCTTTTGTC-3'
IFI44L	Forward	5'-CTTCTCAAAGCCGGGTCATG-3'
	Reverse	5'-AACGCTCTCTCAATTGCACC-3'
IFIT3	Forward	5'-ACGGAGAAAACATCAGCTGA-3'
	Reverse	5'-TGACCTCACTCATGACTGCC-3'
IFITM1	Forward	5'-TCCACCGTGATCAACATCCA-3'
	Reverse	5'-CCAACCATCTTCCTGTCCCT-3'
HERC5	Forward	5'-ACAGTTCACATCCCACCATAG-3'
	Reverse	5'-TCATTTGTAGTCTGTCAGTTCCT-3'
IFI6	Forward	5'-TTACTCGCTGCTGTGCCCAT-3'
	Reverse	5'-CAGGTAGCACAAGAAAAGCGA-3'
IFIT1	Forward	5'-ACGGCTGCCTAATTTACAGCA-3'
	Reverse	5'-GGATAACTCCCATGTAAAGTGA-3'
SCL15A3	Forward	5'-CTTCCAGATGCAGTCCACCT-3'
	Reverse	5'-GGTGGATGTAGTGTAAGCGC-3'
OAS1	Forward	5'-CAAGAGCCTCATCCGCCTAG-3'
	Reverse	5'-TGTTTTCATGCTCCCTCGCT-3'
MX2	Forward	5'-TCTTCGGTTTCCTCCTTTACTGA-3'
	Reverse	5'-CCATTCTCTCGGAGCATAAAATACT-3'
IFNA1	Forward	5'-GTCCTCCATGAGCTGATCCA-3'
	Reverse	5'-CTGGTAGAGTTCGGTGCAGA-3'
IFNA2	Forward	5'-GAAACCATCCCTGTCCTCCA-3'
	Reverse	5'-TATCACACAGGCTTCCAGGT-3'
IFNB1	Forward	5'-CTGCAACCTTTCGAAGCCTT-3'
	Reverse	5'-AGTGGAGAAGCACAACAGGA-3'
IFNL1	Forward	5'-GGAATTGGGACCTGAGGCTT-3'
	Reverse	5'-GTGTGAAGGGGCTGGTCTAG-3'
IFNL2	Forward	5'-TAAGAGGGCCAAAGATGCCT-3'
	Reverse	5'-CTCAGCCTCCAAAGCCATG-3'
ACTB	Forward	5'-GCCAACCGCGAGAAGATGA-3'
	Reverse	5'-AGCACAGCCTGGATAGCAAC-3'
U6 snRNA	Forward	5'-GCTCGCTTCAGCAGCATA-3'
	Reverse	5'-TTTGTGTGTCATCCTTACGC-3'
Vector construction		
Full length TM4SF1-AS1	Forward	5'-GATCCGCTAGGGATCCCTTCAGACGAAATCCTTG-3'

	Reverse	5'-TATCGTCGACAAGCTTTATAGTACGACACTTTTTAATT-3'
Full length PURA	Forward	5'-AGATCTGCCGCCGCGATCGCCATGGCGGACCGAG-3'
	Reverse	5'-GCGGCCGCGTACGCGCATCTTCTTCCCCTTCTTCCTCACCCTGC-3'
Full lentgh YBX1	Forward	5'-AGATCTGCCGCCGCGATCGCCATGAGCAGCGAGGCCG-3'
	Reverse	5'-GCGGCCGCGTACGCGACTCAGCCCCGCCC-3'
MS2 tag	Forward	5'-GTCATAACCGGTTGAGTAGGCTAACAGAATTCGCATGGACGAGCTGTACAAGTCTAAG-3'
	Reverse	5'-GCACTCAACCGGTTCGAGGCTGATCAGCGAGCTCTAGCATTTAGGTG-3'
Templates for in vitro transcription		
T7 TM4SF1-AS1 full length	Forward	5'-CTAATACGACTCACTATAGGGAGACCTTCAGACGAAATCCTTGG-3'
	Reverse	5'-TATAGTACGACACTTTTTAATTCAGAAC-3'
T7 TM4SF1-AS1 antisense	Forward	5'-CTAATACGACTCACTATAGGGAGATATAGTACGACACTTTTTAAT-3'
	Reverse	5'-CCTTCAGACGAAATCCTTGG-3'
T7 TM4SF1-AS1 1-300 nt	Forward	5'-CTAATACGACTCACTATAGGGAGACCTTCAGACGAAATCCTTGG-3'
	Reverse	5'-AAATCAGCACAAAGGTAGAAATG-3'
T7 TM4SF1-AS1 451-754 nt	Forward	5'-CTAATACGACTCACTATAGGGAGAGATTTCAGTGTTCAGCGTCC-3'
	Reverse	5'-TATAGTACGACACTTTTTAATTCAGAAC-3'
T7 TM4SF1-AS1 451-600 nt	Forward	5'-CTAATACGACTCACTATAGGGAGAGATTTCAGTGTTCAGCGTCC-3'
	Reverse	5'-TCTCACTGGTCTGAACTTCA-3'
T7 TM4SF1-AS1 601-754 nt	Forward	5'-CTAATACGACTCACTATAGGGAGAGCCATCATTGGGAGTTGAA-3'
	Reverse	5'-TATAGTACGACACTTTTTAATTCAGAAC-3'
T7 TM4SF1-AS1 1-450 nt	Forward	5'-CTAATACGACTCACTATAGGGAGACCTTCAGACGAAATCCTTGG-3'
	Reverse	5'-CATCCTGCAGAGCTTGCTGT-3'
T7 TM4SF1-AS1 151-600 nt	Forward	5'-CTAATACGACTCACTATAGGGAGACATGACTTTCCAGTAACTGC-3'
	Reverse	5'-TCTCACTGGTCTGAACTTCA-3'
T7 TM4SF1-AS1 301-754 nt	Forward	5'-CTAATACGACTCACTATAGGGAGAAATTTACAACCTGGGGCAAA-3'
	Reverse	5'-TATAGTACGACACTTTTTAATTCAGAAC-3'
T7 TM4SF1-AS1 1-600 nt	Forward	5'-CTAATACGACTCACTATAGGGAGACCTTCAGACGAAATCCTTGG-3'
	Reverse	5'-TCTCACTGGTCTGAACTTCA-3'
T7 TM4SF1-AS1 151-754 nt	Forward	5'-CTAATACGACTCACTATAGGGAGACATGACTTTCCAGTAACTGC-3'
	Reverse	5'-TATAGTACGACACTTTTTAATTCAGAAC-3'
T7 TM4SF1-AS1 301-600 nt	Forward	5'-CTAATACGACTCACTATAGGGAGAAATTTACAACCTGGGGCAAA-3'
	Reverse	5'-TCTCACTGGTCTGAACTTCA-3'

Supplementary Table S2. LncRNA genes with GC patient-specific H3K4me3 peaks

Chr	Start	End	ID
Chr1	95427112	95429222	TCONS_00000250
Chr1	115641025	115642906	TCONS_00000280
Chr1	150134803	150135890	TCONS_00002160,TCONS_00002161
Chr1	198900409	198907406	TCONS_00000111
Chr1	203395100	203398859	TCONS_00001775
Chr2	36581486	36582638	TCONS_00004205,TCONS_00005129
Chr2	48133031	48135298	TCONS_00002890, TCONS_00003671,TCONS_00002891
Chr2	70369788	70370967	TCONS_00004302
Chr2	107159250	107160111	TCONS_00003811,TCONS_00002968
Chr2	158733227	158734311	TCONS_00003916
Chr3	96335577	96337503	TCONS_00006915,TCONS_00006916,TCONS_00006917,TCONS_00006918, TCONS_00006919, TCONS_00007197,TCONS_00006556,TCONS_00007198, TCONS_00007196
Chr3	148942510	148943541	TCONS_00006262, TCONS_00006262
Chr3	149090296	149097098	TCONS_00006264,TCONS_00005618
Chr4	56244631	56246023	TCONS_00008077
Chr4	177301354	177302218	TCONS_00008342
Chr5	23012420	23014631	TCONS_00009370
Chr5	68338248	68339980	TCONS_00009689
Chr6	114192942	114195312	TCONS_00011229,TCONS_00012260
Chr7	6992755	6993974	TCONS_00013687
Chr7	27400765	27405627	TCONS_00013398,TCONS_00012984
Chr8	58657462	58658945	TCONS_00014492
Chr9	109984131	109986116	TCONS_00016664
Chr9	111033974	111035359	TCONS_00016128
Chr9	120544924	120546276	TCONS_00015742
Chr10	4092962	4094242	TCONS_00017698,TCONS_00018092
Chr10	13568821	13570394	TCONS_00018885,TCONS_00018887,TCONS_00018889, TCONS_00018886,TCONS_00018888,TCONS_00018890
Chr10	54788321	54790861	TCONS_00018519,TCONS_00018936,TCONS_00018518,TCONS_00018520
Chr10	124132991	124134068	TCONS_00018622
Chr11	3532516	3535098	TCONS_00019829
Chr11	13007338	13008572	TCONS_00019581
Chr11	67651246	67655841	TCONS_00019351,TCONS_00019099,TCONS_00019352, TCONS_00019353
Chr12	70218378	70223384	TCONS_00020467
Chr12	89412960	89413856	TCONS_00020217
Chr12	90340550	90342179	TCONS_00020510
Chr12	98884043	98885461	TCONS_00020891
Chr13	30994874	30997404	TCONS_00021627
Chr13	76443989	76445794	TCONS_00021496
Chr13	80704943	80705863	TCONS_00021857
Chr13	100146085	100149723	TCONS_00022096,TCONS_00021520,TCONS_00021692,TCONS_00022097
Chr13	114567037	114568086	TCONS_00021917,TCONS_00021918, TCONS_00021919
Chr14	86400375	86402330	TCONS_00022573,TCONS_00022574,TCONS_00022575
Chr14	99752711	99753973	TCONS_00023171,TCONS_00022868
Chr15	85872548	85874187	TCONS_00023765,TCONS_00024194
Chr16	19402889	19405408	TCONS_00024975,TCONS_00024596,TCONS_00024597
Chr16	21244261	21247836	TCONS_00024600
Chr17	6839133	6840683	TCONS_00025293
Chr17	41392072	41393621	TCONS_00025394
Chr17	76982319	76983553	TCONS_00025519
ChrX	20007586	20008754	TCONS_00016924, TCONS_00017045
ChrX	46185477	46187385	TCONS_00017499, TCONS_00017498,TCONS_00017315,TCONS_00016948, TCONS_00017501,TCONS_00017500,TCONS_00017503

Supplementary Table S3. LncRNA genes with healthy individual-specific H3K4me3 peaks

Chr	Start	End	ID
Chr1	1980260	1981602	TCONS_00000453,TCONS_00001374
Chr1	12587336	12589135	TCONS_00000840,TCONS_00000154
Chr1	27560250	27560883	TCONS_00000491,TCONS_00002324
Chr1	90097700	90098529	TCONS_00000019,TCONS_00002404,TCONS_00001571,TCONS_00001572, TCONS_00001573,TCONS_00001574,TCONS_00001570
Chr1	110751973	110752732	TCONS_00000601
Chr1	224803982	224805879	TCONS_00001841,TCONS_00001843,TCONS_00001842,TCONS_00000744
Chr2	3580318	3581850	TCONS_00003159,TCONS_00003157,TCONS_00003158
Chr2	11484856	11485862	TCONS_00003574
Chr2	118560941	118562232	TCONS_00003385
Chr2	118593521	118595010	TCONS_00002986,TCONS_00003824, TCONS_00002987
Chr3	50303118	50305928	TCONS_00005774,TCONS_00006510
Chr3	52278546	52279751	TCONS_00006852
Chr3	64430087	64430944	TCONS_00005531,TCONS_00006042
Chr3	64431321	64431546	TCONS_00005531,TCONS_00006042
Chr5	65220394	65221364	TCONS_00010939
Chr6	16128406	16129117	TCONS_00012092
Chr6	168226066	168227574	TCONS_00011616,TCONS_00011145
Chr6	169845451	169848101	TCONS_00012371
Chr6	170124883	170125929	TCONS_00011423, TCONS_00011627,TCONS_00012835,TCONS_00012374
Chr7	99594506	99595878	TCONS_00013553, TCONS_00013554
Chr7	129780701	129782541	TCONS_00013078 & TCONS_00013079
Chr8	66933546	66934297	TCONS_00015445,TCONS_00015028,TCONS_00015444
Chr8	103539814	103541581	TCONS_00014814
Chr8	103817959	103818930	TCONS_00014499
Chr10	31892593	31893366	TCONS_00018154,TCONS_00017809
Chr10	110225476	110226162	TCONS_00018600
Chr11	63578994	63580794	TCONS_00019653
Chr12	2044679	2046115	TCONS_00020202
Chr12	7281616	7284190	TCONS_00021281,TCONS_00021280
Chr12	125226720	125228068	TCONS_00020613
Chr12	127630330	127631554	TCONS_00020983, TCONS_00020642,TCONS_00020643,TCONS_00021254
Chr13	30170059	30170597	TCONS_00021740
Chr13	44946598	44947682	TCONS_00021985
Chr13	44947868	44948294	TCONS_00021985
Chr14	38066771	38069856	TCONS_00022712
Chr15	41244346	41247548	TCONS_00023380
Chr15	90645776	90646707	TCONS_00024004
Chr16	56676761	56678008	TCONS_00024856,TCONS_00024857,TCONS_00024226,TCONS_00024227
Chr17	33639897	33641975	TCONS_00025355,TCONS_00025356,TCONS_00025358,TCONS_00025359, TCONS_00025360,TCONS_00025361,TCONS_00025896
Chr19	38041513	38042966	TCONS_00027356
Chr20	25227272	25228671	TCONS_00028353
Chr20	39764878	39765864	TCONS_00028390
Chr21	9539885	9540567	TCONS_00029067
Chr21	9540626	9541349	TCONS_00029067
Chr21	10596138	10602364	TCONS_00029076
Chr21	46707245	46708193	TCONS_00029056,TCONS_00028752,TCONS_00029246,TCONS_00028858, TCONS_00029057,TCONS_00029058,TCONS_00029059,TCONS_00029247, TCONS_00029248,TCONS_00029249
Chr22	45664187	45665015	TCONS_00029720
Chr22	45665175	45665876	TCONS_00029720
Chr22	46409222	46410737	TCONS_00029727
ChrX	38080096	38080654	TCONS_00016976
ChrX	124338563	124340445	TCONS_00017242

Supplementary Table S4. Results of targeted sequencing in primary GCs

Patient	Age	Gender	pT	pN	pM	Histology	Gene	Chr	Position	Mutation	Region	Normal var freq	Tumor var freq	P-value
Patient 1	77	Female	T1a	N0	M0	Intestinal	MUC6	Chr11	1013918	C>T	Exon	0.0011	0.3229	0
							TP53	Chr17	7675146	G>C	Exon	0	0.6642	2.40E-47
Patient 2	71	Male	T1a	N0	M0	Intestinal								
Patient 3	80	Male	T1a	N0	M0	Intestinal								
Patient 4	72	Male	T1a	N0	M0	Intestinal	MUC6	Chr11	1018171	T>G	Exon	0.0436	0.2483	1.69E-31
							SEMA3B	Chr3	50274058	G>T	Splice site	0	0.3911	0
Patient 5	67	Male	T1a	N0	M0	Intestinal	TCONS_00021692	Chr13	99500209	T>A	LncRNA intron	0.0015	0.2061	1.73E-95
							TCONS_00021692	Chr13	99500233	A>T	LncRNA intron	0	0.2234	3.74E-112
Patient 6	66	Male	T1a	N0	M0	Intestinal								
Patient 7	69	Female	T1a	N0	M0	Intestinal	APC	Chr5	112839942	C>T	Exon	0.0002	0.8129	0
Patient 8	78	Male	T1b	N0	M0	Diffuse	KRAS	Chr12	25245348	C>A	Exon	0	0.303	0
							TM4SF1-AS1	Chr3	149378727	G>A	LncRNA intron	0.0008	0.2547	4.04E-236

Supplementary Table S5. Proteins detected with RNA pulldown-mass spectrometry analysis

Accession	Symbol	Name	Unused ProtScore	Total ProtScore	%Cov	Peptides (95%)
P38159	RBMX	RNA binding motif protein X-linked	37.69	37.69	68.29	18
Q5U0P9	PURA	purine rich element binding protein A	10.04	10.04	19.88	5
Q96QR8	PURB	purine rich element binding protein B	9.4	9.4	18.91	5
P31942	HNRNPH3	heterogeneous nuclear ribonucleoprotein H3	8.14	8.14	24.86	5
Q6PJV9	SPOUT1	SPOUT domain containing methyltransferase 1	3.05	3.05	9.31	1
Q8N8Y7	RBMX	RNA binding motif protein X-linked	2.96	2.96	21.43	1
Q9P1N0	ILF2	interleukin enhancer binding factor 2	2.02	2.02	8.55	1
Q8NFG3	BX1	BX1 (Fragment)	2	2	34.92	1
Q86U45	HNRNPC	heterogeneous nuclear ribonucleoprotein C	2	2	15.49	1
Q6IPF2	HNRPA1	heterogeneous nuclear ribonucleoprotein A1	2	2	14.06	1
P67809	YBX1	Y-box binding protein 1	2	2	7.1	1
Q96AU2	HNRNPF	heterogeneous nuclear ribonucleoprotein F	2	2	3.86	1
Q15717	ELAVL1	ELAV like RNA binding protein 1	1.7	1.7	7.98	1
Q16629	SRSF7	serine and arginine rich splicing factor 7	1.7	1.7	7.56	1
Q96E39	RBMXL1	RNA binding motif protein, X-linked-like-1	1.16	27.15	65.13	13
Q09666	AHNAK	Neuroblast differentiation-associated protein AHNAK	0.59	0.59	3.48	0

Supplementary Table S6. Genes whose expression was altered by TM4SF1-AS1 knockdown in HSC45 cells

Probe name	Gene symbol	Fold change	P value
A_23_P60079	ANGPT2	56.542145	3.75E-04
A_33_P3392560	LINC00871	21.436792	9.85E-04
A_23_P362694	FDCSP	17.13728	2.87E-04
A_33_P3212394	FRG2C	13.555448	0.001624912
A_21_P0011905	XLOC_l2_007898	12.141504	4.26E-04
A_24_P623782	FRG2	11.88117	7.22E-04
A_23_P62741	ELTD1	9.875498	0.002062708
A_23_P50250	CKM	9.430736	0.048435226
A_21_P0014554	LINC00871	8.888038	0.003780724
A_33_P3251522	AQPEP	8.882653	0.017847462
A_33_P3371325		8.625272	1.19E-04
A_32_P108889	DCLK1	8.517241	0.003204798
A_23_P45185	FIGF	8.419332	0.002760353
A_19_P00318107	XLOC_l2_015561	8.387398	5.06E-04
A_21_P0012579		8.226129	0.003014111
A_33_P3387493	FTH1P18	7.960307	2.76E-04
A_33_P3362611	ELTD1	7.84643	0.001022865
A_21_P0007789	LINC00944	7.340587	1.11E-04
A_33_P3344831	TMEM45A	7.212898	1.12E-04
A_33_P3588134	PANX2	7.0263	0.004622587
A_33_P3375790	RFPL4AL1	6.9913063	3.80E-04
A_32_P130788	SAMD13	6.9412413	0.001570887
A_19_P00322479	LOC644838	6.9039965	0.004889302
A_32_P70927	PAGE2	6.6864843	7.46E-04
A_33_P3422968	DNAH6	6.357939	1.84E-04
A_23_P213319	ADAMTS6	6.3126636	0.00119284
A_21_P0009777	LOC102723931	6.05687	7.41E-04
A_33_P3695899	FLJ31104	6.041572	0.006527758
A_23_P133739	HUS1B	6.025479	0.004538663
A_23_P81898	UBD	5.979295	1.33E-04
A_23_P166269	FAM3B	5.8751054	2.45E-04
A_21_P0014172	HIST1H4H	5.8618183	2.20E-04
A_32_P46214	SLC9A9	5.809666	8.04E-04
A_23_P18649	FAT4	5.805691	0.003092031
A_33_P3366127	RFPL4AL1	5.770718	2.45E-04
A_21_P0007522	lnc-CLEC2D-7	5.668402	0.005393999
A_21_P0003513		5.6087065	2.80E-04
A_23_P62634	RHCE	5.5533733	0.002315334
A_24_P124624	OLR1	5.5463915	0.00555151
A_23_P163402	CYP1A1	5.50868	1.66E-04
A_33_P3299791	LOC644838	5.4737597	0.002740083
A_21_P0001200		5.426798	0.006722334
A_21_P0009499	lnc-TIMM21-3	5.421073	6.24E-04
A_32_P196263	ADAMTS9	5.3953032	0.001285658
A_21_P0013438		5.3611803	0.007838416
A_33_P3363260	PGM2L1	5.3015766	6.58E-04
A_23_P33326	ADRA1B	5.2147183	0.021646084
A_21_P0014071	lnc-HIST2H2AA3-1	5.127367	2.96E-04
A_23_P90453	KRTDAP	5.0672073	3.13E-04
A_21_P0007787	LINC00944	5.048319	4.82E-04
A_19_P00806473		5.04339	1.91E-04
A_23_P5703	LYG2	5.0229416	0.012002256
A_21_P0003300	lnc-AMOTL2-2	5.0048904	0.008820575
A_21_P0006492	lnc-MAOA-2	4.9736543	6.90E-04
A_21_P0001637	lnc-HIST3H3-1	4.9209223	0.007281872
A_33_P3302881	KLHL31	4.8151574	7.81E-04
A_33_P3420862	PAGE2B	4.8146024	4.77E-04
A_21_P0012411	LOC101928430	4.811081	0.004236311
A_33_P3299565	LEKR1	4.8058176	0.00837151
A_23_P94095	ANKRD46	4.722621	7.45E-04

A_32_P96036	MEX3A	4.7181926	6.38E-04
A_21_P0012576		4.6871915	0.001019703
A_21_P0013394	LOC102723946	4.673946	0.008215879
A_21_P0007788	lnc-DHX37-10	4.6611977	0.027739251
A_23_P91512	CLDN14	4.601097	7.79E-04
A_32_P189781	LINC00520	4.58814	0.001398248
A_33_P3270429	NR1D2	4.587277	6.54E-04
A_33_P3323486		4.586427	0.005853048
A_19_P00322435	LOC644838	4.5736156	0.001272006
A_23_P369994	DCLK1	4.5489025	2.45E-04
A_33_P3377649	PRSS16	4.529053	5.12E-04
A_21_P0013914	FAM157B	4.5111084	4.30E-04
A_24_P323941	FAM209A	4.4165554	8.29E-04
A_33_P3395028	LOC152225	4.3949103	0.017892456
A_19_P00323103	UBE2E3	4.3897862	4.43E-04
A_33_P3356361	SRP14-AS1	4.3729396	0.001279297
A_33_P3353372	LMBR1	4.3612437	6.80E-04
A_21_P0004632		4.35733	0.001286041
A_21_P0001751	lnc-FAIM3-2	4.3486676	3.57E-04
A_24_P169048	RFPL3S	4.3404655	0.045360282
A_23_P147786	RIMS2	4.2759595	0.001067743
A_21_P0002532	lnc-AC073043.2.1-1	4.2752223	0.006018415
A_33_P3365611	BTF3L4	4.274502	2.75E-04
A_21_P0007901	lnc-DHX37-10	4.2243767	4.73E-04
A_33_P3232692	IL24	4.2168	8.06E-04
A_33_P3252394	GADD45G	4.2020006	9.48E-04
A_33_P3363395		4.1610584	0.020965409
A_21_P0006017		4.14463	2.91E-04
A_33_P3419865	lnc-C5orf42-2	4.1445093	0.032764215
A_23_P436145	LOC100507431	4.142546	0.002778542
A_33_P3304182		4.134065	0.002256789
A_23_P28834	PHACTR3	4.057036	5.29E-04
A_24_P408736	GALNT5	4.056339	3.77E-04
A_23_P410717	CIART	4.055518	0.017002743
A_21_P0007481	LINC00944	4.0211706	3.58E-04
A_21_P0011408	ULK4P3	4.0043764	5.61E-04
A_21_P0007786	LINC00944	4.000118	0.001227553
A_21_P0004659	LOC101928353	3.9821267	0.001541602
A_33_P3417432	LOC100129098	3.980983	0.001043249
A_23_P145238	HIST1H2BK	3.9737463	4.42E-04
A_33_P3289371	PHACTR3	3.9375677	6.36E-04
A_21_P0003334		3.928217	0.003159988
A_33_P3286953	ADAMTS6	3.9233549	7.68E-04
A_23_P110941	GSTA4	3.9146523	5.43E-04
A_21_P0009752	lnc-UQCRFS1-9	3.902469	7.40E-04
A_33_P3274397	CHM	3.8596618	8.70E-04
A_21_P0002902	LINC01324	3.8450317	0.003605008
A_24_P497464	SOX9-AS1	3.8307214	0.00244307
A_21_P0013255	XLOC_l2_013513	3.825695	0.010063507
A_32_P147078	SLC8A1	3.7951262	0.041457858
A_24_P170234	RNF148	3.7750814	0.001213835
A_21_P0010481	lnc-C22orf26-2	3.7496314	5.09E-04
A_23_P99063	LUM	3.744094	8.88E-04
A_23_P80974	TDO2	3.7335887	0.003051642
A_33_P3222501	FAM157A	3.7164967	0.002725111
A_21_P0013374	LOC101927769	3.7107816	0.014466391
A_23_P123096	GNGT1	3.7062588	0.007463801
A_21_P0009753	LINC00662	3.7050898	5.24E-04
A_24_P62530	RHOU	3.6939669	0.002814427
A_23_P69179	P3H2	3.6793017	4.26E-04
A_19_P00321264	UBA6-AS1	3.6738358	0.001838024
A_21_P0005116	LOC101928353	3.6737769	4.01E-04
A_24_P940115	DLC1	3.6717358	4.43E-04

A_23_P75310	ARHGAP22	3.6519787	5.30E-04
A_23_P52121	PDZK1	3.6505034	0.00463139
A_23_P168761	PTPRZ1	3.6368647	7.26E-04
A_19_P00318725	LINC01204	3.6276655	7.01E-04
A_21_P0002960		3.611924	6.78E-04
A_21_P0003826	lnc-S100P-1	3.6097775	4.40E-04
A_23_P59637	DOCK4	3.6053858	0.030818194
A_19_P00320722	SRP14-AS1	3.5868049	0.005420819
A_32_P191840	LOC644662	3.5850313	0.016332755
A_23_P120694	KCNE2	3.573025	5.61E-04
A_33_P3243093	RGS5	3.5729065	0.001177943
A_33_P3221303	CCR10	3.5570066	0.001973106
A_23_P109488	PIK3IP1	3.5479622	0.00374992
A_21_P0004245	LOC100506639	3.5230038	0.026993653
A_23_P151059	FAM90A1	3.5079772	0.039489273
A_24_P157926	TNFAIP3	3.4980133	0.001494507
A_33_P3213119	HAS2-AS1	3.4860015	0.001022771
A_33_P3429576	AP3S1	3.4739711	0.001368278
A_33_P3288684		3.4723284	0.017405013
A_33_P3420224	ENTPD8	3.4638793	5.88E-04
A_19_P00322310	LINC01094	3.4562235	0.002068624
A_33_P3514487	VSTM1	3.450445	0.001918043
A_21_P0000022	BRI3	3.4432986	0.001006858
A_21_P0000918		3.4422824	0.012858324
A_33_P3345743	PFN1P2	3.4337225	5.07E-04
A_33_P3320538	NUPL1	3.4310696	0.001399566
A_33_P3371402	FAM223A	3.4262655	0.011560242
A_33_P3299025	LOC389834	3.414938	0.03870201
A_21_P0009764		3.4144735	0.008931828
A_33_P3265159	GAPVD1	3.4017212	0.016764311
A_19_P00321263	UBA6-AS1	3.3928478	0.009099657
A_23_P59388	DST	3.3915248	0.006950107
A_23_P127406	KDM4D	3.389265	0.025236534
A_24_P391991	FAM183B	3.3874083	0.005079582
A_23_P371266	DNM3	3.3799717	0.001285484
A_21_P0009754	lnc-UQCRFS1-9	3.371646	0.001615069
A_19_P00315583	LOC100130691	3.3657453	0.002179279
A_33_P3265872	LOC101927497	3.3646948	0.001994368
A_33_P3347147	SUPT3H	3.3617873	0.001455768
A_33_P3705884	LINC00662	3.3544414	5.36E-04
A_19_P00320927	LINC00942	3.348329	5.79E-04
A_33_P3337272	NRARP	3.3465743	5.30E-04
A_33_P3305536	METTL15	3.3422182	0.005276517
A_33_P3307073		3.335659	0.004710593
A_33_P3231814		3.3316834	9.61E-04
A_33_P3305203	RPS6KA5	3.326761	0.01921628
A_21_P0006803	lnc-EBF3-3	3.3198304	0.016748589
A_21_P0004293	lnc-ADAMTS19-2	3.3179488	0.016988438
A_23_P148473	IL2RG	3.313543	0.001602713
A_23_P78782	CA11	3.3024018	0.002909644
A_33_P3271455	PXDN	3.2929337	0.004739542
A_21_P0000539	LINC01138	3.289094	0.001373051
A_19_P00319822		3.2694545	7.96E-04
A_33_P3229239	HIST2H2BF	3.2689893	0.008574538
A_21_P0009125	lnc-IRX3-2	3.2664707	0.001701899
A_21_P0014889	LOC100507431	3.2635763	9.52E-04
A_23_P376591	CLYBL	3.2402134	0.001045388
A_24_P759477	ITGB8	3.2275734	0.001425042
A_21_P0000547	TNIK	3.2185404	0.001097098
A_32_P150735		3.2170532	6.01E-04
A_21_P0000127	SSC5D	3.2128315	0.013093448
A_23_P305616	LOC652276	3.1814966	0.019136237
A_19_P00812924	LOC100507165	3.1811466	0.0055029

A_33_P3309110	ALOX12-AS1	3.1803522	0.002418099
A_21_P0005526	lnc-WASL-1	3.180092	7.90E-04
A_33_P3259943	PLBD1-AS1	3.175178	7.28E-04
A_23_P56746	FAP	3.1672242	0.039068494
A_24_P303145	ANKH	3.1632257	5.95E-04
A_21_P0006886	LOC102723652	3.1616437	9.80E-04
A_24_P515319	FAM90A7P	3.1424313	0.001047655
A_24_P128442	TBX15	3.1396346	0.017622365
A_32_P379379	ATG9B	3.139344	0.013111765
A_21_P0011630	FLJ43681	3.1288145	0.032515876
A_33_P3324186	LOC642366	3.1269324	0.001448174
A_33_P3556532	DNAH17	3.122635	0.008657705
A_23_P160318	COL16A1	3.1183164	6.59E-04
A_19_P00326822	UBE2E3	3.1141846	7.60E-04
A_21_P0003574	lnc-DRD5-10	3.113276	8.64E-04
A_23_P88404	TGFB3	3.0744445	0.001605017
A_33_P3263232	LRRC3	3.0633469	0.025145821
A_19_P00319900		3.058844	0.014349395
A_21_P0005840	LINC01301	3.055715	0.006169656
A_24_P880043	PCGF5	3.0478415	6.97E-04
A_21_P0001018	LOC100996741	3.0421014	0.005639477
A_23_P143535	WDR4	3.0406473	7.31E-04
A_24_P769672	C12orf73	3.0373461	7.44E-04
A_21_P0001545	LOC149351	3.0284986	0.001244828
A_23_P48029	CLEC4A	3.0267735	8.89E-04
A_33_P3429575	LOC643454	3.0256717	9.03E-04
A_23_P64121	KIAA1549L	3.0113368	0.028813465
A_24_P166663	CDK6	3.0056684	9.93E-04
A_23_P259344	CECR6	3.0021224	0.002465607
A_24_P363100	RGMB	2.9944665	0.002715037
A_33_P3415086		2.9913728	0.001191607
A_24_P941268	CA5B	2.9872003	8.45E-04
A_23_P131846	SNAI1	2.985292	8.11E-04
A_23_P250800	ST3GAL6	2.9813533	0.001034369
A_24_P105564	PRKAB2	2.977278	7.96E-04
A_23_P148541	CTAG1A	2.9754665	7.58E-04
A_23_P417974	AQP11	2.974294	0.001747022
A_33_P3239122	PPIH	2.9731803	0.003227285
A_24_P261052	MTMR9	2.9636884	0.001553343
A_33_P3238171	ZDHHC8	2.960811	0.002294787
A_23_P341567	SLC9B2	2.9604886	0.001955647
A_24_P942969	FUT2	2.9553094	9.51E-04
A_33_P3315021	RPL23AP7	2.9524615	0.001458923
A_23_P150343	SLN	2.947428	0.004558841
A_19_P00323040	LINC01322	2.9426181	0.015393163
A_21_P0010480	lnc-SMC1B-3	2.920268	8.40E-04
A_33_P3369461	AMIGO1	2.914447	0.004230631
A_19_P00319376	UBA6-AS1	2.9114428	0.004488969
A_23_P13740	NAV3	2.910128	0.001212391
A_33_P3257252	ZNF551	2.9033217	0.018231181
A_19_P00322409	STXBP5-AS1	2.893406	0.011757487
A_21_P0006728	LINC00704	2.8903358	0.001429456
A_24_P944427	SETD5	2.8888078	0.002762158
A_19_P00322611	lnc-SPAG1-3	2.8833904	0.013465672
A_19_P00317054	SATB1-AS1	2.882177	0.001065415
A_23_P381505	VWDE	2.8808122	0.00982206
A_21_P0000630	LOC643733	2.8770165	0.049467713
A_23_P46045	RGS5	2.8703287	0.015432062
A_21_P0014631	LOC101929787	2.8640647	0.003112219
A_21_P0014637	LOC101927765	2.8619282	0.036154553
A_21_P0001551	LOC102723542	2.8612995	0.011285767
A_21_P0004986	lnc-GMDS-4	2.860543	0.003112144
A_33_P3318322	STEAP4	2.8577094	0.003464489

A_33_P3839897	RNU4ATAC	2.8562343	0.005437078
A_19_P00315633	LOC101927668	2.8539927	8.50E-04
A_23_P32805	GRID1	2.850312	0.034114413
A_21_P0010629	XLOC_l2_000961	2.8458257	0.004489372
A_23_P69958	AP3S1	2.8438957	0.001136014
A_33_P3219840	ZNRD1-AS1	2.8225045	0.007553819
A_33_P3523501	LOC374890	2.818489	0.001523453
A_32_P71571	FAM19A4	2.809707	0.010277399
A_21_P0012368	XLOC_l2_009639	2.8094635	0.00301937
A_24_P149124	NREP	2.807413	0.003262563
A_21_P0013071	XLOC_l2_012925	2.8034134	0.033590067
A_21_P0003575		2.7974808	0.002766335
A_33_P3383724	TRIM61	2.7945228	0.0033548
A_33_P3288074	lnc-ATG2B-2	2.793859	0.002361266
A_33_P3238166	PXDN	2.792264	9.90E-04
A_23_P99540	ZFP36L1	2.7902744	0.001158211
A_21_P0007319	lnc-GALNTL4-1	2.7812984	0.001178472
A_19_P00318314	LOC101927151	2.7792757	0.030106539
A_33_P3316539	SLC7A2	2.7757952	9.26E-04
A_21_P0010562	XLOC_l2_000427	2.77376	0.045379907
A_19_P00316911		2.7653217	0.002676914
A_24_P40529	TMLHE	2.7568843	0.02178784
A_23_P120103	KCNS3	2.7467008	9.55E-04
A_23_P431179	HIST1H4A	2.7409277	0.001522938
A_33_P3210468	MATN4	2.7401123	0.003268605
A_24_P570049	PPARA	2.7361557	0.001693762
A_19_P00322339	LINC00707	2.7359962	0.015547684
A_33_P3256113	GUSBP1	2.7235708	0.03265913
A_21_P0011129	TPTE2P6	2.7231605	0.013152546
A_24_P350683	SLC9A1	2.7208524	0.018551812
A_23_P365248	KCNG4	2.7183137	0.011095022
A_23_P433855	RGS4	2.7126982	0.013389442
A_32_P195401	FAM117B	2.7106895	0.00102101
A_21_P0012781		2.709578	0.001525392
A_23_P397937	SAMD3	2.7088192	0.001026994
A_23_P340131	PRSS16	2.7081034	0.002161157
A_33_P3368323		2.7071414	0.006813012
A_23_P213014	SLC2A9	2.7038202	0.002319971
A_21_P0013312	XLOC_l2_013853	2.7034655	0.023370747
A_21_P0004504	LOC101928505	2.7025936	0.033265244
A_19_P00322576		2.6962063	0.027612394
A_23_P36882	NTS	2.6947265	0.01752146
A_32_P129419	ICE2	2.691144	0.011348358
A_33_P3418394	ATG12	2.6910927	0.001987401
A_33_P3417865	ZFP36L1	2.6901257	0.001125528
A_21_P0010776	PARP8	2.6769934	0.00147776
A_21_P0012776	XLOC_l2_011118	2.671715	0.001750796
A_33_P3380642	FRAS1	2.6622856	0.014307763
A_21_P0004517	TMEM161B-AS1	2.6599905	0.023995122
A_33_P3307980	lnc-FAM133B-1	2.6571975	0.044708353
A_19_P00320846	UBA6-AS1	2.6562202	0.006388252
A_33_P3389113	TRAF3IP2-AS1	2.6549346	0.00673057
A_33_P3326617	FAHD1	2.6535285	0.001274888
A_33_P3286254	AP3S1	2.6507707	0.001107561
A_21_P0008871	FLJ42627	2.6458707	0.008923164
A_33_P3222009	ARL14EPL	2.640448	0.001515321
A_33_P3607359	LOC399815	2.6366057	0.001226995
A_19_P00321549	SATB1-AS1	2.6299045	0.001514552
A_33_P3308167	POLR2J4	2.627766	0.008769497
A_33_P3389298	ZNF30	2.6277454	0.001740414
A_23_P422212	SLC35F3	2.6271155	0.001219878
A_32_P66364	PPP1R1C	2.619359	0.00145404
A_33_P3222341	PITPNC1	2.6192875	0.002231015

A_23_P354827	ZNF550	2.6175592	0.001339138
A_33_P3324814	SATB1-AS1	2.6148732	0.001263156
A_32_P102935	SPDYA	2.6134884	0.001553549
A_23_P70359	AGPAT4-IT1	2.6128976	0.008681143
A_32_P34003	FIGN	2.6122806	0.004110557
A_21_P0004273	lnc-XRCC4-1	2.6119955	0.001118232
A_19_P00315780	LOC101929709	2.6088047	0.006808994
A_33_P3237804	lnc-RP11-351M8.1.1-1	2.603564	0.03101354
A_23_P78018	ABCA5	2.5999029	0.002122651
A_24_P63019	IL1R2	2.5985148	0.001451294
A_24_P212811	ANKRD34A	2.5977402	0.003551572
A_32_P75661	PDIA6	2.5970893	0.030801728
A_21_P0000538	LINC01138	2.5955942	0.002356069
A_23_P169978	ZNF608	2.593349	0.009831995
A_23_P250516	LOC101928710	2.5894048	0.023787435
A_19_P00320723	SRP14-AS1	2.5888712	0.001554329
A_23_P71530	TNFRSF11B	2.5829442	0.001518038
A_21_P0012674		2.5828488	0.001172963
A_23_P204436	GIT2	2.5763035	0.002173518
A_33_P3290394	IL2RG	2.575781	0.002060642
A_33_P3234521	DIS3L	2.575188	0.001574812
A_24_P400172	LOC100130691	2.5733612	0.016552273
A_23_P415015	ATL2	2.5727615	0.005584965
A_33_P3318763	LOC101927497	2.5724723	0.001739405
A_33_P3224795	IKZF5	2.5682411	0.003041969
A_21_P0013256		2.5665011	0.001192393
A_24_P260134	NMNAT3	2.5657794	0.001729764
A_23_P157268	CLDN12	2.564329	0.001302928
A_21_P0014380		2.5604572	0.006607769
A_19_P00319030	LOC100506136	2.5515306	0.00270756
A_24_P350576	TNIK	2.5511136	0.002528457
A_21_P0010449	LOC100506737	2.5493844	0.001230474
A_21_P0007089	PPP2R2D	2.547963	0.001506273
A_33_P3216994	HERC4	2.5441878	0.003958169
A_23_P300056	CDC42	2.543693	0.025842903
A_33_P3378435		2.5409591	0.017853532
A_21_P0011970		2.5398378	0.02222668
A_33_P3228271	CST3	2.536879	0.001435159
A_19_P00803685	ANKRD33B	2.535718	0.04705027
A_33_P3228642		2.5336099	0.004951548
A_24_P4816	GABARAPL1	2.532399	0.004902566
A_23_P374322	LACC1	2.528722	0.049870025
A_23_P255331	MGARP	2.5202038	0.04850564
A_19_P00322407	STXBP5-AS1	2.5190625	0.008924692
A_21_P0011107	ZNF891	2.5150447	0.004300484
A_21_P0007678	lnc-EFCAB4B-3	2.5132656	0.003570027
A_19_P00315738	LOC101929709	2.512431	0.002071664
A_19_P00320579	LINC-PINT	2.5123234	0.001294077
A_19_P00318297	APTR	2.5115678	0.001692646
A_19_P00809382	SETD1B	2.509019	0.04026119
A_33_P3253747	CYP1A2	2.507326	0.001895062
A_33_P3565787	PSMG3-AS1	2.5063162	0.001948796
A_21_P0012507	AADACP1	2.5055194	0.004702823
A_33_P3406245	TAF1A	2.5031466	0.003751065
A_23_P324327	GPRC5B	2.4995434	0.002587324
A_33_P3238402		2.4980164	0.005017712
A_33_P3260654		2.4974468	0.007983109
A_33_P3323842	BDNF-AS	2.496827	0.001807853
A_33_P3416009	DONSON	2.495795	0.034551613
A_21_P0013398		2.4912422	0.002387557
A_33_P3304107	KLHL28	2.490972	0.001345393
A_23_P70328	CENPQ	2.4886613	0.001802289
A_23_P412186	ZNF252P	2.4850135	0.002184166

A_33_P3233135	KIF16B	2.4771914	0.002017307
A_33_P3401990	VPREB3	2.4758878	0.002537323
A_23_P123086	PSMG3-AS1	2.4678571	0.002943153
A_32_P154053	ATG9B	2.4671538	0.002228641
A_21_P0010452	LINC00899	2.4671361	0.024573898
A_21_P0013285	LOC101927668	2.4663045	0.002414021
A_23_P76622	DCT	2.461157	0.003215252
A_33_P3304893		2.4601278	0.001429777
A_23_P404162	HDAC9	2.457268	0.009910041
A_23_P5654	IL37	2.4542296	0.003754881
A_33_P3230259	NCAPH	2.4494815	0.003159443
A_21_P0006040	lnc-ACTL7A-1	2.44887	0.007470671
A_32_P116556	ZNF469	2.4486208	0.021393098
A_21_P0008620	SRP14-AS1	2.4467127	0.006429358
A_33_P3410019		2.4446979	0.002327364
A_23_P69030	COL8A1	2.439361	0.001684572
A_33_P3368695	HES7	2.4385293	0.002016971
A_23_P24176	CCNJ	2.4371738	0.014626814
A_23_P165668	SLC35F5	2.4348128	0.015905162
A_33_P3309201	C16orf95	2.4333398	0.008762213
A_23_P211110	SIM2	2.4314187	0.005647917
A_23_P27332	TCF4	2.4308462	0.001512453
A_23_P20392	PSD3	2.430581	0.00372097
A_21_P0014898		2.4245706	0.00913789
A_21_P0011383	UBE2Q2P2	2.4240825	0.0378375
A_21_P0010632	XLOC_l2_001011	2.4227555	0.004834114
A_33_P3367062	SWT1	2.420886	0.004920072
A_21_P0001608		2.418006	0.031128284
A_21_P0012175	XLOC_l2_009050	2.4153512	0.00747144
A_32_P92505	LCLAT1	2.4150858	0.00230578
A_33_P3531204	C1QTNF9B-AS1	2.4147427	0.001617375
A_21_P0005905	lnc-FAM84B-8	2.4105282	0.002027901
A_21_P0006566	lnc-RAB40AL-1	2.4097679	0.027454488
A_33_P3385351		2.4095314	0.002478241
A_21_P0007726		2.4074743	0.005289653
A_23_P80570	AADAC	2.4062743	0.002777215
A_33_P3300916	AADACP1	2.4050713	0.002758748
A_23_P259863	CD177	2.3996387	0.005659602
A_19_P00317897	LOC101928673	2.3987453	0.007409591
A_23_P38677	SLMO1	2.397812	0.001819726
A_23_P167997	HIST1H2BG	2.3973737	0.001966074
A_21_P0000852	LOC100507412	2.3923647	0.007970128
A_24_P3783	HIST1H2BM	2.3918223	0.002799372
A_32_P440768	ALOX12P2	2.390839	0.023264715
A_23_P200801	PDE4DIP	2.3890243	0.002967515
A_24_P67898	MGEA5	2.3884854	0.003297345
A_33_P3416881	C2orf27A	2.387648	0.001564691
A_33_P3261505	EPG5	2.3863578	0.009460707
A_24_P260440	TNPO1	2.3861752	0.001566211
A_24_P935782	ZNF121	2.3807628	0.003972531
A_21_P0013584	LOC102725126	2.3795245	0.011212465
A_33_P3401673	GIT1	2.3779008	0.030824654
A_33_P3248265	LTB	2.3775733	0.003714285
A_21_P0011337	XLOC_l2_004771	2.3764973	0.04821082
A_21_P0011452	ERVK13-1	2.374452	0.036924686
A_21_P0004718	NQO2	2.3737583	0.00215943
A_23_P315364	CXCL2	2.371659	0.001726781
A_32_P78783	ZNF778	2.3703194	0.00429383
A_24_P359441	CRYBB3	2.3691926	0.005312924
A_33_P3213892	PRDM15	2.3687756	0.001638241
A_21_P0005906		2.3685536	0.001977465
A_33_P3306078	MYH16	2.3664064	0.001626954
A_33_P3421520	IKZF5	2.3582761	0.002249517

A_23_P29723	SGOL1	2.3579254	0.005489147
A_23_P129128	TARSL2	2.3577664	0.005011081
A_33_P3361891	TMPRSS7	2.357576	0.01517892
A_33_P3250438	SMIM7	2.3558483	0.001915338
A_21_P0010453	lnc-C22orf26-2	2.355314	0.002367397
A_33_P3337540	IFNAR2	2.3552032	0.001669635
A_24_P104091	ORAOV1	2.352327	0.002643981
A_23_P68234	GPR75	2.35169	0.038696647
A_23_P35293	GJB5	2.34913	0.004063716
A_23_P122852	SMARCD3	2.3464024	0.002661298
A_24_P29686	ZMIZ2	2.3456354	0.001691844
A_33_P3299872	HINT3	2.344713	0.002099977
A_33_P3340565	PRSS53	2.3444543	0.002277977
A_33_P3280009		2.337826	0.002235972
A_23_P36724	FBXL14	2.3369293	0.001867188
A_24_P262127	RRAD	2.3299732	0.003132288
A_23_P143143	ID2	2.3270762	0.001763214
A_23_P218358	CDRT1	2.3252914	0.002309005
A_19_P00316257	LOC101927668	2.3247335	0.005562091
A_33_P3286536	FUT4	2.3236024	0.002127378
A_23_P136460	FAM13B	2.3216493	0.003324201
A_21_P0005217	lnc-IL6-3	2.3206263	0.004196873
A_32_P112592	LINC01140	2.3191018	0.010451207
A_24_P68908	LOC344887	2.3023784	0.009341903
A_21_P0013751	XLOC_l2_015441	2.3017588	0.007238659
A_24_P917819	ANKRD30BP2	2.3010936	0.021651514
A_23_P73721	RRAGB	2.3005784	0.009649792
A_24_P217234	SLC3A1	2.300437	0.006030453
A_33_P3413732		2.299795	0.002287183
A_21_P0005359	LOC100506178	2.2980232	0.002723132
A_33_P3324810		2.2961028	0.007041038
A_23_P89589	PER1	2.295258	0.004561504
A_23_P427760	PIWIL4	2.2861173	0.003230176
A_33_P3245449	LOC389834	2.2857008	0.002193591
A_21_P0009302	CDRT1	2.2845817	0.003536081
A_23_P25674	CKB	2.2801208	0.037757006
A_21_P0000694	NIFK-AS1	2.2798007	0.003210245
A_21_P0013592	XLOC_l2_014850	2.2762704	0.003538018
A_33_P3265394	WDR74	2.274138	0.047125746
A_33_P3271121		2.2737777	0.005791928
A_21_P0008570	lnc-USP8-2	2.2716835	0.026498398
A_21_P0008354	lnc-TTC9-1	2.2712495	0.005793555
A_21_P0013834	CT45A7	2.270229	0.003299207
A_23_P3552	LOC102723428	2.2684414	0.003387616
A_23_P5568	SFT2D3	2.2651448	0.008529184
A_21_P0003078	LINCR-0002	2.2633529	0.007340451
A_32_P58937	MBTD1	2.2607667	0.002881245
A_23_P364544	C12orf60	2.26	0.003283521
A_24_P46953	SGK3	2.2575624	0.002453872
A_33_P3362148	TTC28-AS1	2.2540958	0.003544195
A_33_P3212754	NUTM2F	2.2535176	0.007213751
A_32_P69368	ID2	2.2522879	0.002843535
A_19_P00322663		2.250177	0.00293623
A_33_P3301469	ANKRD30BL	2.2479463	0.007186989
A_33_P3233005	MBTD1	2.2469516	0.008108617
A_21_P0014024	SATB1-AS1	2.244721	0.002135315
A_21_P0009158		2.244259	0.008256999
A_33_P3816688	PPARGC1B	2.2438564	0.007667218
A_23_P254254	SGSH	2.2399592	0.008495493
A_23_P7896	DUSP22	2.2384858	0.034433443
A_21_P0005949	PVT1	2.2368863	0.002001829
A_32_P36143		2.2331216	0.025951719
A_23_P383132	HIC2	2.2326875	0.00262329

A_33_P3323718	UACA	2.2320316	0.005636556
A_23_P211106	SETD4	2.2306995	0.002094202
A_33_P3244593		2.2300863	0.009961015
A_19_P00800264	OTUD6B-AS1	2.2296896	0.002463095
A_33_P3324505		2.2296178	0.006100114
A_23_P22625	SLC9A6	2.2266269	0.002419117
A_33_P3326432	SEPW1	2.2258196	0.00241284
A_33_P3358626	TAF4B	2.2241576	0.002041136
A_33_P3339650	DST	2.2236373	0.005325315
A_23_P65240	COL4A1	2.2231872	0.04728554
A_33_P3228862	LRRC69	2.2229438	0.006010123
A_33_P3357530	SLC12A7	2.2226548	0.006205211
A_33_P3228322	IL18BP	2.222408	0.005423965
A_23_P214079	SPINK1	2.2221909	0.002092294
A_23_P56553	METTL8	2.2204769	0.002392452
A_23_P209232	CLIP4	2.2190514	0.005424322
A_21_P0014059	PIGC	2.2168844	0.002163301
A_24_P50801	NRP2	2.2165952	0.002338414
A_21_P0000594	BOK-AS1	2.211335	0.012829431
A_23_P388871	HIST4H4	2.2095819	0.020164156
A_32_P12104	ANAPC1	2.2084687	0.002911539
A_32_P129950	NHLRC3	2.2078154	0.005691978
A_19_P00317034		2.206521	0.002843287
A_21_P0010743		2.2042425	0.007602035
A_33_P3299254	VPREB3	2.200639	0.003300576
A_24_P173124	FLCN	2.2005746	0.002638824
A_32_P220750	PLGLB1	2.1997504	0.017658923
A_33_P3384617	XLOC_l2_003882	2.1936352	0.005960056
A_33_P3245454	LOC389834	2.1935313	0.013315892
A_23_P90172	PPP1R15A	2.192144	0.00326618
A_33_P3227556		2.1920774	0.003463425
A_33_P3343828	lnc-CEMP1-1	2.191899	0.002876706
A_23_P342053	RBBP6	2.190769	0.002737267
A_33_P3394395	FAM73A	2.1905875	0.015472497
A_33_P3367196	CNTNAP2	2.1876981	0.014369179
A_24_P310256	LGI4	2.1867824	0.011278221
A_23_P254081	LIAS	2.1856155	0.002988442
A_23_P155666	NAAA	2.1842127	0.002301487
A_23_P138680	IL15RA	2.183917	0.002483761
A_33_P3399634	RASSF10	2.183147	0.003064422
A_21_P0003091	lnc-AC069257.9.1-5	2.182511	0.006393911
A_24_P89891	TRAF1	2.1816058	0.004179164
A_33_P3404759		2.1800194	0.006936099
A_33_P3289426	ZNF775	2.1786711	0.005202974
A_33_P3339336	FAM86FP	2.1773133	0.00271895
A_33_P3396214	KREMEN2	2.1766543	0.003628258
A_23_P33196	COL5A2	2.1763983	0.002945133
A_24_P106910	PTCH1	2.1758127	0.044578467
A_21_P0002741	lnc-PPM1M-2	2.1734333	0.047276422
A_23_P44569	ABCC2	2.1721556	0.019719552
A_23_P401106	PDE2A	2.1707726	0.004677835
A_19_P00813076	LOC101927151	2.1705244	0.019483192
A_21_P0010654	HIST2H2BF	2.1690872	0.00290995
A_33_P3249936	C3orf67	2.1686056	0.00713536
A_23_P345928	METTL25	2.1685228	0.00558398
A_21_P0001374	NBPF15	2.16781	0.002622569
A_33_P3389558		2.1676857	0.004346075
A_24_P886040	DCP2	2.1676314	0.002514187
A_33_P3255229	SETD7	2.1671145	0.004599251
A_21_P0014660		2.1666422	0.004188849
A_21_P0002823	LINCR-0002	2.1666245	0.003415718
A_23_P312358	BEND7	2.1644588	0.010431623
A_23_P59714	MGC16142	2.1644511	0.037065644

A_23_P85903	TLR5	2.1633687	0.006957888
A_32_P46981	HSBP1L1	2.162145	0.002342471
A_23_P393749	CATSPER3	2.1603346	0.006849113
A_33_P3371819	PTCD2	2.1585398	0.004185117
A_32_P149492	NBPF11	2.1578057	0.005036137
A_33_P3247848	ZNF814	2.1562374	0.005454149
A_24_P375205	MKL2	2.1557267	0.00648813
A_32_P94667	PDE4DIP	2.1548665	0.003451035
A_23_P66260	ZNF267	2.1548028	0.002570269
A_23_P379034	BAIAP2L2	2.149972	0.002410038
A_23_P205789	GABPB1	2.149871	0.00430927
A_24_P151	KCNAB2	2.1487637	0.004497804
A_21_P0012067		2.148678	0.02359825
A_23_P72117	SMPDL3A	2.1485612	0.003360936
A_23_P69497	CLEC3B	2.1483107	0.020448163
A_33_P3264528	HOXA11	2.147989	0.002604608
A_33_P3503937	LOC284581	2.1479182	0.002365526
A_21_P0011780	LINC01534	2.147787	0.03703331
A_23_P65068	EID3	2.146795	0.003820764
A_19_P00321339	LOC102724384	2.1458187	0.02038056
A_32_P194779	ZBTB34	2.144608	0.004559499
A_23_P64173	CARD16	2.1429713	0.002504885
A_23_P133408	CSF2	2.1428685	0.002689975
A_23_P253484	AADAT	2.1421895	0.006540505
A_23_P111865	ZSCAN21	2.141286	0.002834088
A_33_P3279109	ZNF75A	2.1409411	0.00290641
A_33_P3214785	LEPROT	2.1408846	0.014104866
A_33_P3254756	UBA6-AS1	2.1403542	0.007763105
A_19_P00321739	LINC00472	2.1377392	0.03324276
A_21_P0010635	LINC01057	2.136448	0.005170828
A_33_P3365988	TGFB2-AS1	2.1298695	0.026708758
A_21_P0001934	lnc-DHRS9-1	2.1294165	0.04109695
A_21_P0013553		2.1291866	0.03785832
A_33_P3424462	CNST	2.12622	0.004396845
A_21_P0010993	LOC101929174	2.1254246	0.040696267
A_33_P3341722		2.1242855	0.003617131
A_33_P3409904	VAMP4	2.1226795	0.00689457
A_24_P336931	ANKRD36	2.1223907	0.002495632
A_33_P3336282	TAF4B	2.1200461	0.004592801
A_23_P96688	SUV420H1	2.119106	0.004134025
A_33_P3220723	KIAA0922	2.1183176	0.003118136
A_23_P382602	BCL9	2.1176739	0.007351313
A_23_P397019	DNAJC25	2.1163175	0.003472808
A_23_P32135	C9orf9	2.1156867	0.005549027
A_21_P0012409	XLOC_l2_009968	2.1143472	0.003231027
A_33_P3320493	RAI1	2.1120427	0.042722773
A_33_P3247175	C4orf47	2.110868	0.01437681
A_33_P3368800	NBPF12	2.110823	0.004233921
A_23_P319565	PGBD3	2.1107125	0.002859296
A_23_P38505	CXCL16	2.1082916	0.003668529
A_33_P3343316	SH3BGRL2	2.1065807	0.005351469
A_21_P0011510	ALOX12P2	2.1054852	0.006448097
A_19_P00321383		2.105082	0.002691234
A_33_P3339687	ZNF669	2.104639	0.029907167
A_24_P171058	TMEM64	2.1041512	0.00593089
A_24_P118011	PRORSD1P	2.103782	0.016506672
A_24_P332081	JAKMIP3	2.1024494	0.007495532
A_23_P387471	MICB	2.102423	0.030915538
A_33_P3354267	AKIRIN1	2.0997608	0.00280331
A_23_P150609	IGF2	2.0993905	0.002698865
A_21_P0010633	LINC01057	2.0986295	0.003755679
A_23_P151133	TSPAN9	2.0985591	0.002976553
A_33_P3351999		2.09797	0.003999764

A_23_P342600	STK35	2.096195	0.00766607
A_33_P3260455		2.0960665	0.004072531
A_23_P128940	VCPKMT	2.0956643	0.00300372
A_21_P0003320	LOC100996286	2.0954034	0.03380032
A_23_P161507	MTL5	2.0951178	0.004210283
A_24_P409494	BCL2L13	2.0926337	0.005580861
A_23_P216568	FAM206A	2.0923703	0.003163538
A_21_P0000023	RIOK2	2.0921433	0.003246274
A_23_P23443	EFHD2	2.0908775	0.002671141
A_19_P00318183	LINC01197	2.0901487	0.003068817
A_33_P3256778	CNTF	2.0899558	0.005886301
A_33_P3374190	INPP5A	2.0896058	0.004720216
A_24_P18146	PSD3	2.088881	0.003105478
A_23_P217968	SUV420H1	2.0884974	0.002870117
A_21_P0014184	lnc-GLCCI1-2	2.0861099	0.036095593
A_21_P0010775	PARP8	2.0850317	0.003897053
A_33_P3327822	SH3BGR	2.0828187	0.015848553
A_33_P3363355	ICAM4	2.0813344	0.004628265
A_21_P0012815	LOC102723888	2.0810711	0.03890549
A_33_P3399248	UFM1	2.0801046	0.002914541
A_33_P3347937	HSF1	2.079343	0.025684714
A_33_P3379017	HIC2	2.0788834	0.022970568
A_21_P0014094		2.0781107	0.014680336
A_21_P0009763	lnc-ANKRD27-2	2.0765166	0.010604191
A_21_P0003885	LRPAP1	2.0757127	0.005096579
A_24_P284584	ZNF559	2.0756128	0.008620925
A_24_P194748	ZNF275	2.075146	0.0072534
A_24_P239606	GADD45B	2.0749876	0.003022886
A_33_P3322730		2.0746791	0.007442316
A_21_P0013727	LOC101928413	2.0743067	0.003045316
A_32_P215938	GPSM1	2.0739467	0.003337595
A_24_P257416	CXCL2	2.0720942	0.002949484
A_33_P3285047	ZNF75A	2.070542	0.005956265
A_33_P3255631	ZNF780A	2.0695148	0.005093383
A_23_P127948	ADM	2.0692368	0.006540628
A_33_P3280603	PPP4R1L	2.0689223	0.048183367
A_23_P44836	NT5DC2	2.0677047	0.007124629
A_24_P261567	GDPD5	2.0666268	0.040011726
A_33_P3777584	SERAC1	2.0662553	0.004688374
A_33_P3245066	SLC35E2B	2.0643327	0.002951203
A_21_P0012215		2.06351	0.023906982
A_23_P112774	PTP4A3	2.0604057	0.004697217
A_33_P3422439		2.0596294	0.025183223
A_33_P3211634	PPIL6	2.058613	0.011155571
A_33_P3419545	CDADC1	2.0584536	0.003796231
A_23_P1691	MMP1	2.0578809	0.003742439
A_23_P19673	SGK1	2.0571885	0.004399689
A_23_P74663	TAF1A	2.0567133	0.007189121
A_33_P3367850	CHRM4	2.0560172	0.021280922
A_33_P3325467	SMIM12	2.055647	0.004570478
A_33_P3342345	XLOC_l2_004817	2.055311	0.032968804
A_32_P110390	TMEM171	2.0549653	0.006964875
A_33_P3326927	ZNF19	2.0526662	0.022447618
A_33_P3797820	LOC102723652	2.0523791	0.005274509
A_21_P0012273	NBEA	2.0508337	0.009029138
A_24_P192805	CARD17	2.0496361	0.010253011
A_24_P392022	EEF2KMT	2.049233	0.004352545
A_21_P0013960	LOC101930246	2.0491757	0.004620124
A_23_P254212	RPA4	2.0463321	0.014451476
A_19_P00322977	LINC-PINT	2.0454645	0.003007682
A_24_P365015	HOXB13	2.0453463	0.011469111
A_23_P7325	BST1	2.0411665	0.016115405
A_33_P3405789	TULP3	2.040952	0.008412275

A_33_P3242863	NT5M	2.039989	0.003913104
A_33_P3363091	VAC14	2.0399885	0.00648794
A_24_P100368	DYNLT3	2.039178	0.004082388
A_24_P16913	ABCC4	2.0388052	0.016253177
A_32_P232035	LOC100270746	2.038136	0.005569504
A_21_P0013523	OTUD6B-AS1	2.0367653	0.005243811
A_23_P501805	LIPT1	2.0358808	0.003083327
A_23_P47885	LRIG3	2.0356064	0.003246393
A_24_P795371	NR2F2-AS1	2.0354977	0.007342006
A_24_P21447	SURF6	2.0341542	0.003167084
A_33_P3245412	TRMT10B	2.0339582	0.003775242
A_23_P414308	FLCN	2.0330431	0.003530843
A_33_P3278911	PHF20L1	2.031573	0.013324673
A_23_P13094	MMP10	2.0313258	0.005318083
A_33_P3862375	USP45	2.029774	0.003345891
A_23_P20970	ATG12	2.029585	0.003146248
A_21_P0011951	XLOC_l2_008203	2.0295327	0.003574472
A_21_P0010742		2.0283349	0.010370392
A_33_P3252800	PTPRR	2.0275235	0.003237856
A_23_P27724	SEPW1	2.0275037	0.004636312
A_21_P0011758	XLOC_l2_007097	2.0271442	0.005321061
A_21_P0001704	lnc-NBPF16-4	2.026628	0.003433566
A_23_P254120	FBXO9	2.0259423	0.003431693
A_33_P3419835	FBXL19-AS1	2.0248542	0.037398003
A_33_P3389728	NR5A2	2.023881	0.003566312
A_33_P3399064	RNA5-8S5	2.0238602	0.003262517
A_21_P0000898	MIR181A1HG	2.023117	0.015828986
A_21_P0011949	XLOC_l2_008203	2.0216265	0.004717266
A_21_P0012780		2.0206764	0.005177946
A_24_P147252	ZNF23	2.0197067	0.004921486
A_19_P00315631	LOC101927668	2.0195816	0.015727822
A_21_P0011536	LRRC37BP1	2.0175931	0.013363573
A_33_P3354796	FLJ42627	2.017027	0.007330732
A_19_P00321886	LINC-PINT	2.0169253	0.003151682
A_21_P0012208	lnc-C21orf58-1	2.0152304	0.014494773
A_33_P3420816	GDF1	2.0142884	0.021432688
A_23_P138461	C10orf2	2.0141275	0.00596721
A_21_P0010239		2.0131311	0.008394382
A_23_P428219	EZH1	2.0122776	0.004384397
A_33_P3289286	TMEM57	2.0115037	0.007815767
A_33_P3345001		2.0104034	0.008105206
A_33_P3371999	TPPP	2.0089657	0.004171401
A_23_P202905	TIRAP	2.0089183	0.00457948
A_23_P74778	C1orf54	2.0088308	0.003194176
A_21_P0008517	LOC100506476	2.008419	0.01146649
A_24_P176079	WASF3	2.0080075	0.004342369
A_23_P39542	C2orf76	2.0077922	0.004658972
A_19_P00322260	LOC101929709	2.0072691	0.011849981
A_21_P0006912	lnc-SMC3-1	2.0069096	0.007416176
A_23_P47924	PTPRR	2.005187	0.006880164
A_33_P3239455	GTF2IRD2B	2.0032666	0.024939153
A_21_P0014175	LOC100506302	2.0029812	0.01129932
A_24_P925635	SEPT7P2	2.0027502	0.016691912
A_33_P3330353	MOSPD1	2.001049	0.00352457
A_19_P00325336		2.0009985	0.038310222
A_23_P69537	NMU	-2.0003507	0.003455797
A_33_P3308446	RHOB	-2.000514	0.007238018
A_24_P921933	SRSF1	-2.0034933	0.010602064
A_23_P165783	MLPH	-2.0039997	0.003409183
A_23_P348257	NUAK1	-2.0052772	0.003216404
A_21_P0001178	lnc-GOLPH3L-1	-2.0054574	0.013246782
A_23_P4160	NBR2	-2.006334	0.03495231
A_23_P253586	DOPEY2	-2.0067208	0.009621458

A_23_P42909	TMEM139	-2.00918	0.003526633
A_33_P3344169	DYX1C1	-2.0098677	0.012910576
A_23_P151307	RAPGEF3	-2.0126734	0.008006577
A_23_P331748	CD33	-2.0164351	0.003287999
A_33_P3351536	PTK2B	-2.0172293	0.006937373
A_23_P3643	DNASE1L2	-2.017279	0.014343778
A_24_P236935	KLK6	-2.0174258	0.006109681
A_33_P3423121	RDX	-2.0190945	0.006363282
A_33_P3865368	LOC254896	-2.020177	0.003889506
A_33_P3253596	KIF4A	-2.0225773	0.006218116
A_24_P408047	PLEKHA4	-2.0227454	0.003400649
A_24_P68079	TRANK1	-2.022923	0.007672886
A_24_P235266	GRB10	-2.0236976	0.005485836
A_19_P00320759	FLJ32255	-2.0237849	0.011519634
A_23_P410613	TMEM263	-2.0246978	0.003198267
A_23_P210900	ACSS2	-2.0261037	0.004304908
A_33_P3397399	PPP3CB	-2.0261824	0.027935393
A_24_P148796	MST1	-2.026949	0.004662524
A_21_P0014313		-2.027168	0.003346019
A_23_P84872	SECISBP2	-2.0283446	0.005023708
A_23_P365738	ARC	-2.0306733	0.016224507
A_23_P159125	SLC16A5	-2.0310478	0.003930452
A_23_P148047	PTGER4	-2.0318046	0.003980678
A_23_P146444	CORO2A	-2.032329	0.011092204
A_23_P35617	PLCE1	-2.032845	0.007752727
A_23_P157793	CA9	-2.0329275	0.013632862
A_33_P3338559	RBBP4	-2.0353076	0.005861934
A_24_P134727	TFAM	-2.0373394	0.014125912
A_23_P372848	P2RX1	-2.0378556	0.010088713
A_33_P3290567	WEE1	-2.0394099	0.003040243
A_33_P3303212	CCDC74A	-2.0399494	0.004799526
A_23_P259166	TCEAL4	-2.0399897	0.003531459
A_23_P77661	ZNF720	-2.040056	0.005050858
A_33_P3227225		-2.041559	0.009012925
A_23_P390097	TTC39B	-2.0424042	0.015852852
A_23_P33894	MAGED2	-2.0438035	0.003078346
A_24_P161018	PARP14	-2.0443933	0.003292712
A_33_P3413083	RHEB	-2.0454023	0.003580472
A_32_P720220	LINC01559	-2.0462642	0.003647014
A_23_P157628	DEFB4A	-2.0471714	0.010940613
A_33_P3258392	EDN1	-2.0493724	0.011300291
A_23_P52761	MMP7	-2.0500357	0.00326503
A_24_P69095	ENC1	-2.0540283	0.004118657
A_23_P96965	SYNC	-2.0541642	0.0035881
A_33_P3387145	SH3KBP1	-2.0558884	0.008483267
A_23_P134925	BNIP3L	-2.0578244	0.003052995
A_32_P202759	FAM171B	-2.0590653	0.003701675
A_23_P71379	PSCA	-2.0603125	0.0056471
A_33_P3349947	TCP1	-2.0612667	0.003119837
A_33_P3322724	POLR3A	-2.061681	0.004272394
A_23_P167269	TMA16	-2.0621204	0.004745028
A_23_P3042	PPP2R5E	-2.0644405	0.002869613
A_33_P3380417	SLC25A30	-2.0680351	0.009227547
A_33_P3276693	PGF	-2.070056	0.002828791
A_23_P100203	HSBP1	-2.0714395	0.004018243
A_23_P35082	SESN2	-2.0719235	0.004662927
A_24_P67534	SMIM10	-2.0722399	0.005454397
A_23_P203665	ACER3	-2.0726752	0.003566505
A_33_P3298980		-2.0740414	0.006434071
A_23_P97123	ANKRD36BP1	-2.0749886	0.00591929
A_21_P0004593		-2.0753884	0.008807305
A_33_P3494748	TMEM65	-2.0763538	0.002903131
A_33_P3258061	PALM3	-2.0776193	0.009564115

A_21_P0013462		-2.0783699	0.00331333
A_21_P0006520	lnc-ATP2B3-1	-2.0788167	0.015593748
A_24_P38347	DPYSL2	-2.0790403	0.00617608
A_24_P254551	ARHGEF9	-2.079248	0.003713943
A_24_P873414	PLEKHB2	-2.0808396	0.003219724
A_23_P206059	PRC1	-2.0816672	0.002783165
A_33_P3503537	LOC285178	-2.082478	0.005462611
A_19_P00323454	KANSL1-AS1	-2.082517	0.008740024
A_32_P18440	ARID5B	-2.0833874	0.004367198
A_21_P0007133		-2.0863564	0.006630396
A_23_P334870	TMEM217	-2.0869546	0.008269058
A_23_P381102	CCDC74B	-2.087682	0.007871661
A_23_P130515	CEACAM3	-2.0878847	0.006703384
A_23_P359277	ELOVL7	-2.0880778	0.002877147
A_23_P109881	ITIH4	-2.0884578	0.0268562
A_23_P86900	B4GAT1	-2.0934157	0.002777269
A_23_P46170	MED8	-2.0934541	0.002643682
A_23_P501933	CACNG6	-2.093742	0.002874352
A_19_P00320440	LINC00662	-2.0954237	0.006151612
A_23_P87082	ROBO3	-2.0956597	0.00767842
A_23_P121795	SORBS2	-2.0961697	0.00426854
A_24_P331704	KRT80	-2.0963657	0.002870122
A_23_P132159	USP18	-2.098858	0.003849482
A_23_P57306	CHAF1B	-2.099529	0.003003629
A_33_P3231297	CREG1	-2.1002982	0.002770887
A_23_P315206	CCBL1	-2.1017823	0.009730545
A_32_P218025	LOC100506253	-2.1021175	0.003981647
A_21_P0011456	PLA2G10	-2.1021373	0.007509693
A_23_P10182	ACOX2	-2.10246	0.002935863
A_32_P231446	HIPK1	-2.1038191	0.003101218
A_23_P89410	BECN1	-2.1042037	0.002923851
A_23_P108437	FZD5	-2.1050892	0.003123174
A_23_P53126	LMO2	-2.106353	0.003329007
A_23_P93641	AKR1B10	-2.107807	0.003195762
A_24_P390060	IQCD	-2.1078913	0.006971908
A_21_P0001676	lnc-BTBD19-1	-2.110945	0.008893146
A_23_P213102	PALLD	-2.1115754	0.002674161
A_24_P23995	RNF187	-2.1126504	0.002576282
A_23_P372096	NOL4L	-2.1137624	0.005806275
A_32_P178966	TMEM170B	-2.1146467	0.004735326
A_23_P411612	SPRYD4	-2.1150434	0.019740814
A_23_P8196	ME1	-2.1164165	0.003733024
A_33_P3380992	AKR1B15	-2.1171825	0.00482895
A_19_P00321259	LINC01133	-2.1177394	0.003247993
A_23_P14774	CTSH	-2.1189141	0.002521896
A_23_P202810	OVOL1	-2.1191328	0.002991255
A_33_P3307495	STRA6	-2.1203897	0.003711849
A_23_P418199	LOC730098	-2.1207376	0.00312954
A_21_P0014096		-2.121305	0.020418642
A_24_P16124	IFITM4P	-2.122957	0.011837528
A_24_P944299	CDC42BPA	-2.1233687	0.003209366
A_24_P37540	ARPC4-TTLL3	-2.1244154	0.006482344
A_24_P371962	AMD1	-2.1248987	0.002553176
A_23_P400298	PRSS22	-2.1251233	0.003888528
A_24_P334640	PAQR8	-2.127717	0.022430064
A_23_P148475	KIF4A	-2.1301112	0.003398434
A_23_P218579	GLB1L	-2.130402	0.00252229
A_23_P209735	ARMC9	-2.1305685	0.003305579
A_21_P0007885	LOC643770	-2.131256	0.003598702
A_24_P345451	CYBRD1	-2.133121	0.022605747
A_23_P24784	TNNI2	-2.1338372	0.036932707
A_33_P3263841	RCHY1	-2.134291	0.002901928
A_21_P0009781	UCA1	-2.1383185	0.01592829

A_24_P376339	CCNL2	-2.138506	0.00253468
A_23_P152949	LRRC46	-2.1390269	0.00480613
A_23_P166823	TNNC1	-2.1391912	0.002874457
A_23_P133058	MRFAP1L1	-2.1392787	0.002607843
A_24_P230938	MORN4	-2.1396773	0.029521374
A_23_P137016	SAT1	-2.1398287	0.003181492
A_33_P3316223	SNAPC1	-2.1402946	0.00532155
A_33_P3302957	PLEKHG4	-2.140343	0.016577592
A_21_P0014894		-2.1412501	0.006213323
A_33_P3268313	PGAM2	-2.1420832	0.007572125
A_23_P167308	RNF4	-2.143557	0.002417343
A_33_P3371718	SAT1	-2.144131	0.002900543
A_24_P30194	IFIT5	-2.1445136	0.0358973
A_24_P703830	NANOS3	-2.144719	0.003755946
A_33_P3237096	INPP5F	-2.1464798	0.003215285
A_33_P3210099	ALPK3	-2.1468165	0.009171036
A_33_P3309859	lnc-FAM43A-2	-2.1474879	0.0069003
A_33_P3210278	SYNE2	-2.147996	0.00293258
A_19_P00809417	ATF7IP2	-2.152589	0.002503634
A_24_P101617		-2.1526058	0.004673892
A_24_P307580	HTATIP2	-2.152929	0.003160537
A_24_P112160	UPK3B	-2.1563385	0.003502069
A_32_P66881	TLR4	-2.156874	0.006102856
A_33_P3338186	HEXDC	-2.1576517	0.002867477
A_23_P58506	ELL2	-2.1585326	0.003566678
A_33_P3390778	TRIM46	-2.1612122	0.004918966
A_21_P0011633	KRT14	-2.161231	0.020060038
A_24_P398940	CASC4	-2.1648414	0.004591031
A_21_P0007747	LOC643770	-2.1651208	0.007476148
A_24_P254705	ZNF695	-2.1659951	0.002546679
A_33_P3278058	PIH1D2	-2.1676724	0.003259886
A_32_P152437	AKAP12	-2.1679986	0.003005606
A_23_P219197	RGS3	-2.1689038	0.002938688
A_24_P928052	NRP1	-2.1691403	0.00259658
A_24_P916141	DCAF7	-2.1700044	0.003698708
A_24_P921897	HOOK1	-2.170619	0.005397609
A_19_P00317272	LINC01191	-2.173837	0.01919296
A_23_P82588	C7orf55	-2.1748648	0.003581719
A_33_P3258782	AP1S2	-2.1750448	0.004711327
A_24_P77082	KMO	-2.1751933	0.002281901
A_23_P8083	LY6G6C	-2.1756103	0.005211407
A_24_P752208		-2.1774788	0.013246406
A_19_P00315717	FAM200B	-2.1778793	0.00248073
A_33_P3447441	lnc-WDR1-1	-2.178343	0.002370967
A_33_P3296181	CCL3L3	-2.1789675	0.003033403
A_23_P86021	SELENBP1	-2.1808076	0.00278056
A_24_P181101	TMEM135	-2.1815436	0.00252776
A_24_P48204	SECTM1	-2.1820319	0.003287043
A_24_P128524	ICMT	-2.1843772	0.04568627
A_23_P117782	LARP6	-2.185081	0.00300297
A_24_P64126	CPNE3	-2.186058	0.002605083
A_23_P216225	EGR3	-2.188331	0.003858259
A_24_P944616	HP1BP3	-2.1894076	0.002328445
A_23_P431346	PRR15	-2.1902342	0.002249342
A_23_P152082	SPTBN5	-2.190615	0.004176032
A_24_P129341	AKR1B10	-2.1919005	0.003777612
A_33_P3309556	PTPRE	-2.1927197	0.009725562
A_33_P3332215	MUC1	-2.195751	0.003509686
A_23_P200493	LBR	-2.1960404	0.002457945
A_32_P37592	SCARNA17	-2.1960866	0.003172485
A_24_P323598	ESCO2	-2.196669	0.002598139
A_24_P331560	STS	-2.1975994	0.004632051
A_32_P86763	TGM2	-2.1981711	0.004392372

A_23_P103617	ANXA9	-2.1996253	0.002378017
A_23_P2271	PTHLH	-2.199753	0.003367313
A_21_P0003294	lnc-NAA50-2	-2.201331	0.009772847
A_23_P69738	RASL11B	-2.2014985	0.040172797
A_23_P113793	ZBED2	-2.2020004	0.002341719
A_23_P156687	CFB	-2.2060692	0.02565863
A_24_P270728	NUPR1	-2.2078977	0.002898963
A_33_P3388391	GJB4	-2.2080228	0.002622858
A_33_P3270863	XDH	-2.2088647	0.002172546
A_33_P3243702	KLHL30	-2.2096121	0.009676041
A_33_P3318581	PLOD2	-2.21089	0.002441879
A_21_P0004564	lnc-AC008394.1-1	-2.2119775	0.00692281
A_24_P174503	AMT	-2.2141757	0.006867558
A_24_P927189	OXNAD1	-2.2143452	0.002925522
A_23_P154411	PPIG	-2.2152255	0.006055099
A_23_P102037	COQ10B	-2.2157784	0.002792652
A_33_P3392391	CPT1C	-2.217171	0.017853802
A_32_P209094	FGGY	-2.2180293	0.002609081
A_33_P3306153	KIAA1841	-2.2189722	0.003297517
A_23_P214821	EDN1	-2.221394	0.002176677
A_23_P118203	ZG16B	-2.2224402	0.004991957
A_23_P210763	JAG1	-2.2236905	0.002690995
A_33_P3398597	EPS8L1	-2.2240589	0.002629229
A_32_P88310	LOC730183	-2.2243972	0.005738013
A_24_P115511	RAB14	-2.2276363	0.019221596
A_24_P917886	MUC5AC	-2.228636	0.002210307
A_19_P00321009	LINC01133	-2.2297213	0.00220741
A_33_P3214466	MESP1	-2.2299645	0.002257878
A_33_P3290919	BAG1	-2.2307873	0.002231087
A_21_P0002549	lnc-GPR55-2	-2.2310526	0.028282223
A_21_P0000132	C16orf93	-2.2331908	0.029321853
A_23_P64617	FZD4	-2.2341475	0.002161633
A_23_P26024	C15orf48	-2.243176	0.002099013
A_24_P190472	SLPI	-2.2433932	0.002770536
A_33_P3402615	SLC6A9	-2.246126	0.002146823
A_23_P218817	CPT1B	-2.2480052	0.002730879
A_23_P98995	CALCOCO1	-2.2483172	0.002115154
A_23_P394395	JPH2	-2.2496433	0.017249933
A_23_P252306	ID1	-2.2515943	0.002303258
A_23_P141715	TPGS2	-2.2517192	0.001935466
A_23_P257164	AMT	-2.2525988	0.003103421
A_23_P137984	S100A10	-2.2531836	0.002147159
A_23_P50000	FAM57A	-2.2547712	0.004004962
A_23_P417415	ACOT11	-2.2565799	0.002566224
A_24_P267592	SAMHD1	-2.25708	0.002204498
A_23_P316487	H2AFV	-2.2573564	0.002225689
A_23_P121527	KLHL5	-2.2576234	0.002319453
A_23_P146654	BAG1	-2.2587976	0.002287329
A_23_P217384	AP1S2	-2.2615592	0.002225365
A_23_P38167	GPRC5C	-2.2645102	0.002220921
A_23_P82868	PLAT	-2.264847	0.001917225
A_24_P52697	H19	-2.2691944	0.002195033
A_21_P0014013		-2.2710164	0.002187229
A_33_P3232504	CYSRT1	-2.2726328	0.003490462
A_23_P91140	PECR	-2.272936	0.00429829
A_23_P116235	MDK	-2.2741911	0.002149233
A_23_P46936	EGR2	-2.2749846	0.003174444
A_24_P364236	NDUFC2	-2.2755098	0.002677951
A_24_P9090	HNRNPDL	-2.2767248	0.00341907
A_24_P253251	SLC7A1	-2.279076	0.002612826
A_33_P3252884	PHF12	-2.279237	0.002221793
A_33_P3352307	RPS6KA1	-2.2795677	0.003112798
A_19_P00323082	H19	-2.2798016	0.004002925

A_24_P95439	CARS	-2.2820966	0.002045019
A_23_P409438	IFNL2	-2.2832026	0.002267617
A_33_P3728167	CDH5	-2.2838159	0.005289935
A_32_P176018	ACTL8	-2.2838886	0.002089123
A_33_P3328772	PLEKHA5	-2.2847946	0.035949226
A_23_P128817	PCK2	-2.2854888	0.003656985
A_33_P3330498	ALDH7A1	-2.2857246	0.010554615
A_23_P15348	MPRIP	-2.286103	0.003554202
A_19_P00321010	LINC01133	-2.2861102	0.00259595
A_32_P14610	PDLIM5	-2.286796	0.001987623
A_24_P228130	CCL3L3	-2.286892	0.003160371
A_21_P0014351	SP2-AS1	-2.287338	0.003866935
A_33_P3235410	PTPLA	-2.287981	0.003420347
A_23_P106741	PSMD7	-2.2893007	0.00327732
A_23_P133543	KLHL3	-2.2895584	0.010248584
A_24_P450285	CCDC153	-2.2910106	0.002043617
A_21_P0007626		-2.2929332	0.012870353
A_33_P3397940	CALML3-AS1	-2.2972438	0.006766254
A_23_P138194	NCF2	-2.298043	0.001872252
A_23_P137856	MUC1	-2.3011632	0.002359101
A_23_P217737	ATP7A	-2.3018427	0.002269121
A_33_P3360341	GATA3	-2.3037863	0.001803706
A_23_P389919	WHSC1	-2.306138	0.001818221
A_23_P416468	PIF1	-2.308387	0.004393946
A_33_P3386117	RER1	-2.3087249	0.001904942
A_33_P3238920	INPP5F	-2.3093004	0.002945815
A_24_P84608		-2.309736	0.003730817
A_32_P76720	NT5DC3	-2.309852	0.002715836
A_21_P0013113	XLOC_l2_013189	-2.3143542	0.003787187
A_24_P158421	SAR1A	-2.3212183	0.002576238
A_33_P3290924	BAG1	-2.3219724	0.002088364
A_24_P295010	SERPINB9	-2.3246036	0.002189778
A_33_P3389827	PROM2	-2.325261	0.00265469
A_23_P24157	PYROXD2	-2.326433	0.007791312
A_23_P135722	BTC	-2.3273087	0.003816631
A_33_P3246505	MAP3K8	-2.3284056	0.007433513
A_33_P3288659	ACTL8	-2.3289137	0.004460415
A_24_P248606	ACSL3	-2.329013	0.001733559
A_23_P252471	PECAM1	-2.329763	0.005334836
A_33_P3866631	DKFZP564C152	-2.3300393	0.001861716
A_33_P3499174	URM1	-2.3309205	0.002668628
A_24_P120251	TM4SF18	-2.332246	0.004011405
A_23_P21207	UBA7	-2.3393648	0.00306308
A_23_P23438	SEMA4A	-2.340282	0.002967184
A_33_P3423979	PALLD	-2.3405647	0.001726723
A_24_P857624	TNRC6C-AS1	-2.3409772	0.002689214
A_33_P3318668	CYSRT1	-2.3466768	0.002077994
A_33_P3217704	FAM214B	-2.3477006	0.009941967
A_24_P80532	CCNG2	-2.3478882	0.007298547
A_33_P3461416	GP6	-2.3483434	0.003325216
A_23_P103601	MAN1C1	-2.3491342	0.006658063
A_33_P3314176	FAM46C	-2.3526366	0.01537869
A_23_P125643	ASB9	-2.3542655	0.001722254
A_23_P86100	KLHDC9	-2.3563473	0.002602277
A_23_P4190	ACSF2	-2.357932	0.001874972
A_33_P3234697	LXN	-2.3621187	0.001954838
A_23_P54469	ISL2	-2.3623502	0.006336391
A_23_P64721	HCAR3	-2.3651211	0.010993619
A_23_P215227	DNAJB6	-2.3706558	0.001869127
A_23_P30495	HMGCR	-2.3724556	0.001649062
A_21_P0005252	LOC340340	-2.3744366	0.002214966
A_23_P205959	ALDH1A3	-2.3750997	0.002040342
A_33_P3313796	CCDC34	-2.3771894	0.001715134

A_24_P41570	H2AFZ	-2.3790247	0.001941638
A_24_P335305	OAS3	-2.3795893	0.00340444
A_32_P32653	SENP5	-2.3807592	0.001740263
A_23_P166459	LGALS1	-2.3876512	0.002028134
A_33_P3332547	IQCJ-SCHIP1	-2.3882992	0.003100894
A_32_P163147	VSIG1	-2.3892035	0.004693987
A_33_P3326210	ESCO2	-2.3899946	0.002640285
A_23_P216455	RLN2	-2.3903253	0.001628082
A_24_P1255	BCCIP	-2.391139	0.002020888
A_23_P11286	HNRNPH2	-2.3967533	0.002452869
A_32_P56249	USP30-AS1	-2.403358	0.02276962
A_23_P90732	PNKD	-2.4055555	0.003291604
A_23_P87049	SORL1	-2.4057138	0.001542546
A_23_P500300	TRIM15	-2.4144425	0.001515104
A_33_P3285911	KIAA1919	-2.4162164	0.001681671
A_21_P0013246	UPK3B	-2.4190166	0.001936294
A_23_P156880	ENPP1	-2.4239388	0.010977859
A_33_P3333800	NOXO1	-2.4278946	0.002232962
A_33_P3391603	LAMA4	-2.4312181	0.001551611
A_33_P3540143	IL17RA	-2.4321225	0.001758102
A_33_P3228593		-2.4327898	0.017018802
A_24_P255218	MYO5A	-2.4354932	0.002923626
A_33_P3844650	ANGPTL2	-2.4359329	0.01646894
A_23_P385771	PAOX	-2.437751	0.003740839
A_23_P250735	CBX7	-2.4397125	0.001447689
A_33_P3367396	FAM177B	-2.439784	0.03233022
A_33_P3321432	FAM198B	-2.4399598	0.004086967
A_23_P99661	ARHGEF40	-2.4406736	0.003270668
A_24_P329487	FAM84B	-2.4408963	0.007397488
A_23_P6651	FAM208A	-2.4417746	0.006752389
A_24_P236091	ENO2	-2.4419844	0.002854733
A_23_P83579	ARNT2	-2.4444098	0.008449281
A_24_P393470	MEF2BNB	-2.445131	0.001644537
A_24_P50245	HLA-DMA	-2.4473677	0.00638138
A_23_P500861	SYNE1	-2.448261	0.00669805
A_24_P10233	DAPK2	-2.4489756	0.002482705
A_33_P3369844	CD24	-2.4504313	0.001481011
A_33_P3292540	CDKN2C	-2.4505188	0.001438392
A_23_P93881	SYPL1	-2.451295	0.00252498
A_23_P328545	GABRP	-2.4530678	0.015335645
A_21_P0000687	NPPA-AS1	-2.4549716	0.013084812
A_33_P3405213	PECAM1	-2.4566824	0.001476461
A_33_P3272395	RAB19	-2.4626224	0.01422585
A_23_P2674	KRT4	-2.4649196	0.001504377
A_24_P396650	RPS6KA1	-2.4721665	0.002565414
A_24_P243749	PDK4	-2.475127	0.001529245
A_24_P336718	RABIF	-2.4765499	0.019446503
A_33_P3799692		-2.477683	0.001745889
A_33_P3344579	DLD	-2.482217	0.003284406
A_23_P137543	ZNF362	-2.4847383	0.001991514
A_24_P842006	C16orf93	-2.4877965	0.013164157
A_33_P3358342	SIAH2	-2.4924922	0.001309946
A_33_P3340154	DGCR14	-2.4926794	0.001420301
A_33_P3410459	SCARB2	-2.4931326	0.001460788
A_33_P3278941	REC8	-2.4946282	0.001707394
A_33_P3230254	NCAPG	-2.4992166	0.001829316
A_24_P71661	CRTAP	-2.4992476	0.00269793
A_23_P111194	SPDEF	-2.5002916	0.001377409
A_23_P106463	OAZ2	-2.5106483	0.001361118
A_23_P250607	PLS3	-2.513801	0.001672004
A_23_P92161	ARL14	-2.514883	0.001627548
A_33_P3251054	RNF38	-2.517362	0.001432091
A_33_P3341490	KRT42P	-2.5179892	0.002985139

A_24_P678104	STMN3	-2.5222893	0.001457109
A_23_P18684	CLGN	-2.5232353	0.006409074
A_23_P62081	SCG5	-2.5282907	0.001689892
A_23_P24004	IFIT2	-2.5341802	0.001271653
A_21_P0007235	lnc-P2RY2-2	-2.5343707	0.001394554
A_33_P3210585	AAK1	-2.536488	0.00186819
A_24_P8220	HS6ST1	-2.5424204	0.0012248
A_21_P0010500	GNAS-AS1	-2.5428746	0.001214962
A_23_P148556	ABCD1	-2.5466988	0.002105791
A_23_P304897	BDKRB2	-2.5481148	0.001230192
A_33_P3360972	EXOC3L4	-2.551479	0.001482283
A_23_P259621	LAT2	-2.55175	0.001445541
A_23_P416178	CCDC136	-2.5592656	0.002316057
A_23_P256391	GOLGA4	-2.5595903	0.00170139
A_23_P134714	HRSP12	-2.5639958	0.001885272
A_23_P63243	C1orf43	-2.5642815	0.00197019
A_33_P3241511	SERPIND1	-2.5694962	0.00124652
A_23_P406135	IFT172	-2.5701463	0.001912266
A_23_P42306	HLA-DMA	-2.580227	0.001314486
A_33_P3248439	MVB12B	-2.580229	0.00131547
A_33_P3268507	CEACAM1	-2.5814288	0.002325382
A_21_P0000069	VEPH1	-2.5830338	0.001160961
A_23_P53176	FOLR1	-2.586062	0.001690379
A_33_P3417695	ODF3B	-2.5927587	0.002964977
A_24_P412976	TMEM143	-2.5957043	0.001586377
A_23_P39766	GLS	-2.5977402	0.001157079
A_21_P0002781	LINC01213	-2.6007607	0.001297539
A_23_P210210	EPAS1	-2.6072657	0.001455084
A_24_P175187	SAMD9	-2.6109397	0.002217213
A_33_P3358740	OSBPL7	-2.6122212	0.001105084
A_21_P0000015	PARP9	-2.6123304	0.002404938
A_33_P3385870	CCNG2	-2.6233137	0.001517198
A_21_P0011928	PEBP1	-2.6295114	0.003023037
A_21_P0007329	lnc-CCDC34-1	-2.6309452	0.006026684
A_23_P29684	VILL	-2.6342165	0.001296374
A_33_P3410194		-2.6446948	0.001218224
A_24_P304071	IFIT2	-2.6538227	0.002961431
A_24_P29595	STXBP5	-2.6543825	0.002363629
A_23_P339309	LIN52	-2.6583745	0.002021625
A_23_P145694	ASNS	-2.6623354	0.001619032
A_23_P102706	SNPH	-2.665381	0.001363072
A_33_P3333317	OPTN	-2.6670275	0.001231967
A_33_P3347869	C3	-2.6695523	0.001324437
A_33_P3289218	AAK1	-2.6726027	0.001206808
A_33_P3376249	S100A2	-2.680301	0.001255048
A_23_P500000	SCEL	-2.6807678	0.001149498
A_23_P53193	SYTL2	-2.683562	0.001187006
A_23_P142574	MOGAT1	-2.6902714	0.001166194
A_23_P156017	GOLPH3	-2.693187	0.001174233
A_33_P3220919	ADRBK2	-2.6947215	0.001073493
A_23_P65442	IRF9	-2.6968694	0.001249924
A_23_P59950	SLC39A14	-2.70024	0.001464253
A_33_P3420416	LGALS9	-2.702586	0.007546025
A_23_P11224	MMGT1	-2.7060652	0.001519917
A_19_P00326514		-2.7062132	0.001250066
A_32_P9382	MZT1	-2.7074063	9.72E-04
A_23_P41390	SH3TC1	-2.7163398	0.005921239
A_23_P329573	ITGB2	-2.7237668	0.002206422
A_23_P217114	ALAD	-2.727831	0.002822263
A_24_P101651	CSAG4	-2.730128	0.00159206
A_24_P902728	CAPRIN1	-2.7312598	0.001047993
A_33_P3340025	GINS1	-2.7316608	9.68E-04
A_33_P3351101	TYSND1	-2.7337124	0.002221655

A_33_P3216610	TMPRSS4	-2.7364464	0.001095901
A_33_P3377209	ENSA	-2.7444253	0.001002082
A_23_P40240	CTSZ	-2.7487047	0.001484603
A_33_P3374623	ABCA7	-2.7537515	9.33E-04
A_23_P80048	FER1L4	-2.7543068	0.001294833
A_24_P12435	NCOA7	-2.7555897	0.001001925
A_19_P00805833	ATF7IP2	-2.7562082	0.001175023
A_33_P3304170	PIK3CG	-2.7572541	9.51E-04
A_23_P13604	PEBP1	-2.7623303	0.003282635
A_23_P88865	CMTM3	-2.7650168	9.26E-04
A_33_P3307875	SPTSSA	-2.7662764	0.001023063
A_23_P255057	SLC35A5	-2.7674515	0.002567085
A_21_P0000603	ASB9P1	-2.7676797	0.017972067
A_33_P3359084	TAS1R3	-2.7742915	9.79E-04
A_33_P3340342	CMTM3	-2.7869194	0.002613251
A_23_P209055	CD22	-2.789982	0.016245142
A_23_P155052	APOL6	-2.790268	0.001153622
A_33_P3393170	CAPN5	-2.7914796	0.001771627
A_33_P3413905	ADM2	-2.7968674	0.002709856
A_33_P3369153	KIF3C	-2.7976148	0.007907297
A_21_P0007122	ZBED5-AS1	-2.7998188	0.002975827
A_32_P44394	AIM2	-2.8001926	0.001430455
A_21_P0010626	PSAT1	-2.8028765	0.00103886
A_23_P339240	PLCH1	-2.803311	0.005990509
A_23_P85952	DENND2D	-2.803344	0.001067878
A_32_P229746	DNAJB6	-2.8056016	0.001066158
A_23_P139912	IGFBP6	-2.806464	0.002912123
A_21_P0009342	WFDC21P	-2.807646	0.002160662
A_21_P0010773	FLJ43315	-2.8115988	8.64E-04
A_33_P3316587	SLC22A18AS	-2.8137987	0.001679936
A_23_P84576	ANTXR1	-2.8164403	0.004743026
A_33_P3298139	CRLF2	-2.8240604	0.006503127
A_23_P127140	RAB11FIP2	-2.8258739	0.007568856
A_33_P3363188	FLJ43315	-2.8320918	9.89E-04
A_33_P3300308	MAP1LC3A	-2.8322635	0.007692381
A_24_P354689	SPOCK1	-2.838123	0.001318362
A_33_P3244803	ACOX1	-2.8518193	8.38E-04
A_21_P0013177	XLOC_l2_013293	-2.8525877	0.001496112
A_23_P48596	RNASE1	-2.8585298	9.12E-04
A_24_P33982	MILR1	-2.8593113	9.95E-04
A_23_P347468	FZD3	-2.8605793	8.88E-04
A_23_P140146	IFI27L2	-2.8631442	0.001161453
A_33_P3253234	IQSEC2	-2.8692946	8.24E-04
A_21_P0011175	CASC4	-2.8739028	0.004921899
A_32_P46238	KRT17P5	-2.8780181	0.01575803
A_33_P3630780	lnc-EGR3-1	-2.883024	0.015221559
A_23_P88767	PLA2G10	-2.8874967	8.03E-04
A_33_P3395014	ACOXL	-2.892203	0.007059033
A_33_P3214635	FECH	-2.8946097	0.001081342
A_23_P81048	STIM2	-2.898807	8.81E-04
A_23_P89941	CDKN2D	-2.8998313	8.38E-04
A_23_P89030	C16orf95	-2.9030478	0.001317038
A_24_P294832	PTP4A1	-2.9045777	9.28E-04
A_19_P00805702	C1GALT1	-2.90575	0.003012453
A_23_P315345	PIN4	-2.909041	0.001100421
A_23_P388681	ELAVL1	-2.9096398	0.001102717
A_24_P37409	DUSP2	-2.9115148	0.001992428
A_24_P221485		-2.918168	0.001755131
A_24_P7594	APOL6	-2.9366145	0.001457572
A_23_P209731	ARMC9	-2.94353	0.004084607
A_23_P54758	GDE1	-2.948725	0.001828088
A_24_P372134	TMEM140	-2.9489014	0.003664372
A_21_P0000058	PROM2	-2.954769	8.19E-04

A_32_P87013	CXCL8	-2.9578924	0.025887154
A_23_P204269	USP15	-2.95874	9.19E-04
A_33_P3333982	PDE4DIP	-2.9590774	0.008725762
A_33_P3303649	MB	-2.9633482	0.001311973
A_24_P413988	TGOLN2	-2.980391	0.001152269
A_33_P3697530	SEMA4D	-2.985638	0.001162371
A_33_P3379916	GLS	-2.9872403	0.001751167
A_23_P104073	S100A3	-2.9922395	0.001049407
A_33_P3382177	TIMP2	-2.9934313	0.001036448
A_32_P113508	ATG16L1	-2.9975808	7.50E-04
A_33_P3363420	FRMD3	-2.9984448	8.23E-04
A_32_P205241	GJA3	-3.0072994	0.01725774
A_23_P207879	CARD14	-3.0108957	0.009713897
A_33_P3336422	ZBED5-AS1	-3.0110626	0.001019365
A_23_P81158	ADH1C	-3.0118504	0.002515264
A_23_P49499	ST6GALNAC2	-3.018724	0.001078249
A_33_P3230526	MPRIP	-3.0194504	0.030561604
A_24_P38081	FKBP5	-3.0205903	0.001820982
A_23_P253524	CENPE	-3.0248227	8.44E-04
A_23_P154358	PROM2	-3.0380318	8.06E-04
A_23_P63050	UROD	-3.0396907	0.001459329
A_23_P141447	RDM1	-3.0408492	0.010713586
A_21_P0000002	ACBD7	-3.0452018	0.013861166
A_23_P23074	IFI44	-3.0568717	7.67E-04
A_33_P3373750	BRD4	-3.0601208	6.60E-04
A_23_P312851	PMEL	-3.0641413	0.001430047
A_23_P250358	HERC6	-3.0660417	0.004575132
A_23_P100001	FAM174B	-3.0718615	0.001103774
A_32_P206899	DNAH2	-3.0733407	9.21E-04
A_33_P3253807	CEBPG	-3.0795774	6.47E-04
A_33_P3296846	TMPRSS4	-3.0813851	0.004158984
A_23_P94703	TOR1B	-3.0836337	0.001128962
A_21_P0011933	LOC442028	-3.087673	7.34E-04
A_33_P3315719	PLEKHH2	-3.0949783	0.001369328
A_33_P3229107	MIR205HG	-3.095541	0.00107585
A_33_P3290443	SCARNA9	-3.1017962	9.32E-04
A_21_P0013514	XLOC_l2_014504	-3.118028	0.001303449
A_23_P38346	DHX58	-3.12657	8.19E-04
A_23_P398566	NR4A3	-3.128605	7.12E-04
A_23_P67785	SPAG16	-3.1316016	7.26E-04
A_21_P0013475		-3.139935	7.93E-04
A_24_P941167	APOL6	-3.1433396	6.12E-04
A_23_P105012	HRASLS2	-3.146403	7.21E-04
A_33_P3327108	NLK	-3.1467524	0.001850743
A_21_P0012551		-3.1491387	7.19E-04
A_33_P3356910	TCEAL6	-3.149284	7.94E-04
A_23_P421306	SYT12	-3.1533544	0.001270575
A_33_P3408757	FOXO6	-3.1576872	0.001010253
A_23_P103104	MFNG	-3.1698334	9.47E-04
A_23_P169437	LCN2	-3.1763377	6.84E-04
A_23_P43763	PLLP	-3.1764495	0.01691517
A_33_P3248794	NAB2	-3.1967797	6.09E-04
A_23_P71148	BLVRA	-3.197458	6.09E-04
A_24_P673786	PIP4K2A	-3.2015696	9.94E-04
A_23_P35645	RBM17	-3.215803	8.39E-04
A_24_P66001	UQCR10	-3.2202232	6.19E-04
A_23_P64828	OAS1	-3.2223868	0.001005329
A_33_P3336686	CLIC3	-3.2292397	9.64E-04
A_24_P175176	PHTF2	-3.2321255	0.008853568
A_33_P3315320	CNTD1	-3.2409792	0.00326139
A_33_P3330911	BCAS1	-3.2459378	0.001220207
A_33_P3380523	PRTFDC1	-3.2460754	0.004157415
A_23_P307536	SH3D21	-3.2467644	9.17E-04

A_23_P388433	C4orf3	-3.2572405	8.52E-04
A_23_P259692	PSAT1	-3.2709515	0.001637809
A_23_P101407	C3	-3.2768428	0.001082468
A_23_P380318	EGR4	-3.2860537	5.57E-04
A_23_P146456	CTSV	-3.288896	6.81E-04
A_33_P3295358	ANGPTL4	-3.293526	5.51E-04
A_32_P416161	XKRX	-3.3090918	0.021359062
A_33_P3278362	ANKRD2	-3.3145251	0.001163464
A_23_P386320	MFI2	-3.3261728	0.002596486
A_24_P822704	TMEM198	-3.3311005	0.001448941
A_33_P3321034	PLXNA4	-3.344593	0.010312194
A_23_P333218	ERGIC1	-3.3456433	5.09E-04
A_23_P17663	MX1	-3.3542967	6.73E-04
A_32_P41065	TMCC1	-3.3665142	9.32E-04
A_23_P107775	TMEM190	-3.3678718	0.003554116
A_23_P85693	GBP2	-3.370017	5.87E-04
A_21_P0000351	SCARNA22	-3.3868916	0.00177823
A_24_P413941	C2orf69	-3.3937333	7.88E-04
A_33_P3363425	FRMD3	-3.4305859	4.91E-04
A_23_P88351	ATL1	-3.4407537	0.003311631
A_21_P0013512		-3.4497871	0.002096286
A_33_P3266828	TMEM8B	-3.4644296	5.16E-04
A_23_P47704	UCP2	-3.4692445	8.68E-04
A_23_P123672	TDRD7	-3.4729495	0.001370494
A_24_P301557	LPIN2	-3.4746344	0.00305387
A_32_P524904	C11orf86	-3.4793289	0.001146577
A_33_P3315779	HERC6	-3.4954371	0.019949904
A_23_P98631	HPS5	-3.4998686	6.68E-04
A_33_P3378514	PDE5A	-3.503786	6.04E-04
A_23_P62890	GBP1	-3.5053468	0.001439089
A_19_P00321671	lnc-MMRN1-2	-3.509635	0.038597982
A_23_P430902	MORN4	-3.5210931	0.001034107
A_24_P53282	CPD	-3.523147	7.98E-04
A_24_P238499	TYMSOS	-3.5298846	6.07E-04
A_24_P933418	ABI2	-3.5355103	6.67E-04
A_23_P252541	RAB7B	-3.5359006	0.001201407
A_32_P192545	TCEAL6	-3.545403	4.90E-04
A_33_P3408918	SAA2	-3.5473945	0.001842779
A_32_P18470	TCEAL5	-3.5476015	0.001456515
A_23_P6771	LMCD1	-3.5566945	5.73E-04
A_23_P32404	ISG20	-3.5625465	6.34E-04
A_23_P69383	PARP9	-3.5822601	5.68E-04
A_33_P3416414		-3.6104832	0.002195468
A_23_P82047	STXBP5	-3.6236997	0.001551095
A_23_P152838	CCL5	-3.6474864	8.76E-04
A_23_P404494	IL7R	-3.6484177	4.02E-04
A_32_P452655	LGALS9C	-3.6488428	4.90E-04
A_33_P3384462	THSD4	-3.6518886	6.02E-04
A_23_P38537	KRT16	-3.6547258	5.72E-04
A_33_P3243887	IL11	-3.6561332	5.82E-04
A_33_P3223495		-3.6707382	4.93E-04
A_24_P45446	GBP4	-3.6789339	3.94E-04
A_23_P146417	TMEM245	-3.6794415	4.83E-04
A_23_P23346	MLLT11	-3.6847184	8.50E-04
A_32_P166693	HEG1	-3.6910074	0.004687741
A_23_P87013	TAGLN	-3.7052066	0.002759304
A_33_P3219596	LINC00925	-3.7078571	5.71E-04
A_21_P0011517	KRT14	-3.7097156	0.001720863
A_23_P75786	SLC15A3	-3.7413273	8.91E-04
A_23_P142918	C2orf69	-3.8013065	0.001805462
A_24_P882732		-3.8217678	4.26E-04
A_23_P104318	DDIT4	-3.823902	5.08E-04
A_23_P116557	LGALS9	-3.8248272	7.05E-04

A_32_P76627		-3.8305404	0.001014943
A_24_P382319	CEACAM1	-3.854888	6.65E-04
A_21_P0000479	SNORA11D	-3.8710442	5.93E-04
A_23_P202720	SLC35C1	-3.8754096	3.76E-04
A_19_P00317815	LOC101927934	-3.8895836	0.005338546
A_23_P106675	PLCG2	-3.9007113	4.64E-04
A_21_P0005651		-3.9224954	0.020726396
A_33_P3209229	RAB26	-3.949619	3.52E-04
A_23_P96158	KRT17	-3.95526	7.69E-04
A_23_P58266	S100P	-3.9564445	5.40E-04
A_24_P228611	FER1L4	-3.9703703	0.00298979
A_21_P0011578		-4.009164	4.41E-04
A_33_P3237977	LARP6	-4.0166416	4.15E-04
A_33_P3215640	PI16	-4.0284085	4.83E-04
A_33_P3406072	FRMD3	-4.043311	0.003013872
A_21_P0007217	lnc-LGALS12-2	-4.061233	0.002753245
A_33_P3283611	IFIT3	-4.0851607	3.16E-04
A_33_P3239954	USP14	-4.102555	0.001569971
A_23_P139500	BHLHE41	-4.103315	6.04E-04
A_24_P274270	STAT1	-4.1144314	4.53E-04
A_24_P240259	LRRC31	-4.115681	3.51E-04
A_24_P11061	CSAG1	-4.121627	5.79E-04
A_24_P346101	PRELID2	-4.1545863	3.38E-04
A_24_P887857		-4.1715727	4.07E-04
A_23_P132515	SIDT1	-4.1784153	0.04344236
A_23_P431388	SPOCD1	-4.195264	3.55E-04
A_21_P0008670	LINC00925	-4.1978793	0.001741401
A_33_P3348164	TTLL7	-4.2090974	6.25E-04
A_24_P220485	OLFML2A	-4.22765	0.005408548
A_24_P322771	TFF1	-4.267451	3.78E-04
A_23_P41114	CSTA	-4.3135757	0.006955415
A_33_P3857239	KRT42P	-4.3399773	2.74E-04
A_23_P113237	CRLF2	-4.378316	0.013888253
A_33_P3306983	C11orf31	-4.3825293	3.46E-04
A_23_P42353	ETV7	-4.395603	0.022561993
A_32_P62963	KRT16P2	-4.403206	3.45E-04
A_23_P23947	MAP3K8	-4.4118757	0.049031716
A_23_P340318	C11orf31	-4.477005	0.00113765
A_23_P64611	P2RY6	-4.494579	0.004333936
A_24_P567298		-4.5076036	0.00123198
A_23_P12343	GSTM3	-4.508561	3.60E-04
A_33_P3345534	KRT14	-4.527597	0.001013996
A_23_P202658	GSTP1	-4.5299377	2.91E-04
A_23_P87011	TAGLN	-4.547944	5.23E-04
A_23_P60130	MAL2	-4.5716515	5.01E-04
A_23_P74799	SLC25A24	-4.592331	3.19E-04
A_33_P3269924	HIP1R	-4.619397	2.54E-04
A_33_P3372910	DDX58	-4.626783	2.55E-04
A_33_P3354451	TRIM31	-4.6537995	5.26E-04
A_33_P3304172	MUC6	-4.7363405	0.028403768
A_23_P145874	SAMD9L	-4.7513313	2.30E-04
A_23_P85783	PHGDH	-4.8102612	4.71E-04
A_23_P25868	NAA30	-4.822734	2.11E-04
A_33_P3264846	SAMD9L	-4.85777	2.40E-04
A_32_P105549	ANXA8L1	-4.860423	2.43E-04
A_23_P23048	S100A9	-4.88148	0.005113259
A_21_P0012601	lnc-MMRN1-2	-4.9339676	4.07E-04
A_23_P819	ISG15	-4.9537516	3.54E-04
A_23_P45871	IFI44L	-4.980685	2.43E-04
A_24_P111996	HFE	-5.041593	2.87E-04
A_24_P236799	RAB31	-5.070511	4.26E-04
A_24_P146683	MSMB	-5.126778	5.82E-04
A_23_P1962	RARRES3	-5.1863785	2.63E-04

A_33_P3418170	DDX58	-5.244879	2.83E-04
A_23_P415021	METTL7A	-5.2795553	1.86E-04
A_23_P71480	DEFB1	-5.441025	2.56E-04
A_23_P210554	SPATA2	-5.516813	4.79E-04
A_23_P41470	DDX60	-5.6164594	1.88E-04
A_23_P105794	EPSTI1	-5.6367702	2.41E-04
A_21_P0000310	SNORA12	-5.671422	0.005590105
A_23_P344421	ROBO4	-5.6825824	1.85E-04
A_33_P3269723	ZSWIM7	-5.713958	0.001918953
A_23_P139786	OASL	-5.71754	1.82E-04
A_23_P110196	HERC5	-5.8465447	1.64E-04
A_23_P254507	HOPX	-5.900045	0.005021634
A_23_P207564	CCL4L2	-5.9560556	0.022537807
A_23_P204782	MDM1	-6.3432384	0.020106312
A_33_P3376971	CHAC1	-6.3481274	2.63E-04
A_23_P337800	IFNL1	-6.348483	3.05E-04
A_33_P3268622	LY6D	-6.368071	5.64E-04
A_23_P57364	TFF2	-6.382899	1.86E-04
A_33_P3376965	CHAC1	-6.4491024	6.37E-04
A_23_P324754	CEMIP	-6.476768	3.82E-04
A_21_P0002779	TM4SF1-AS1	-6.4824886	0.035774566
A_33_P3419988		-6.4910836	0.017306907
A_24_P28722	RSAD2	-6.4992404	1.13E-04
A_23_P39955	ACTG2	-6.5228825	0.013829938
A_23_P307544	PLXNA2	-6.551103	1.18E-04
A_23_P104199	ITGB1	-6.592889	1.86E-04
A_21_P0014466	LOC101928152	-6.6245875	1.14E-04
A_23_P19663	CTGF	-6.688004	0.001198801
A_23_P143713	APOBEC3G	-6.892527	6.28E-04
A_23_P56356	PLB1	-7.005197	1.08E-04
A_23_P94186	LYPD2	-7.1444798	0.007460184
A_23_P201376	SSX2IP	-7.226971	1.00E-04
A_33_P3339865	CALML5	-7.6100807	1.03E-04
A_23_P52266	IFIT1	-7.690957	9.57E-05
A_21_P0006537		-7.78396	9.62E-05
A_23_P95790	ITLN1	-7.8084974	0.005018879
A_23_P166797	RTP4	-8.412615	1.32E-04
A_33_P3259203	TMEM255B	-8.522111	0.007456157
A_33_P3535175		-8.566684	0.001722696
A_33_P3225522	OAS2	-8.639452	2.68E-04
A_33_P3354607	CCL4L2	-8.725195	8.99E-04
A_23_P156327	TGFBI	-8.844732	7.09E-05
A_21_P0014514		-9.418101	0.001937676
A_21_P0013865	LOC102725299	-9.95734	9.73E-05
A_24_P102650	MUC5B	-10.704958	1.69E-04
A_33_P3423941	IFITM1	-11.446535	3.03E-04
A_33_P3225512	OAS2	-11.777884	8.85E-05
A_23_P72737	IFITM1	-14.079743	4.32E-05
A_21_P0002780	TM4SF1-AS1	-14.11726	1.16E-04
A_24_P557479	XAF1	-14.976566	2.97E-04
A_23_P39465	BST2	-16.492905	3.03E-05
A_33_P3220911	BST2	-17.992977	6.20E-05
A_23_P204087	OAS2	-22.569792	6.25E-05
A_24_P270460	IFI27	-23.609858	3.92E-05
A_23_P201459	IFI6	-23.966183	1.90E-05
A_23_P6263	MX2	-66.47237	9.81E-06

Supplementary Table S7. Proteins identified with ChIRP-MS

Accession	Description	Sum PEP Score	Coverage [%]	# Peptides	# PSMs	# Unique Peptides	Score Mascot	Score Sequest HT
P04908	histone H2A type 1-B/E [OS=Homo sapiens]	36.025	38	6	166	2	1421	211.14
P07900	Heat shock protein HSP 90-alpha [OS=Homo sapiens]	23.28	11	7	90	2	851	78.61
Q92945	Far upstream element-binding protein 2 [OS=Homo sapiens]	18.314	8	5	52	4	531	69.86
P36551	Oxygen-dependent coproporphyrinogen-III oxidase, mitochondrial [OS=Homo sapiens]	17.873	13	4	18	4	55	25.69
Q7RTS7	Keratin, type II cytoskeletal 74 [OS=Homo sapiens]	17.285	4	4	46	1	295	47.6
Q99832	T-complex protein 1 subunit eta [OS=Homo sapiens]	17.06	12	5	32	5	344	29.11
P23396-1	40S ribosomal protein S3 [OS=Homo sapiens]	16.962	22	5	38	5	282	44.45
P62249	40S ribosomal protein S16 [OS=Homo sapiens]	16.627	32	6	28	6	415	23.56
P46783	40S ribosomal protein S10 [OS=Homo sapiens]	16.433	21	3	30	3	470	41.18
P07737	profilin-1 [OS=Homo sapiens]	16.138	30	4	42	4	638	72.24
P52597	Heterogeneous nuclear ribonucleoprotein F [OS=Homo sapiens]	14.133	6	2	18	2	226	31.96
P63104-1	14-3-3 protein zeta/delta [OS=Homo sapiens]	13.658	11	3	47	1	499	42.5
P26583	High mobility group protein B2 [OS=Homo sapiens]	12.861	13	3	30	1	265	36.14
P63244	Receptor of activated protein C kinase 1 [OS=Homo sapiens]	12.096	12	4	24	4	136	30.17
P67809	Nuclease-sensitive element-binding protein 1 [OS=Homo sapiens]	12.074	8	3	67	3	518	57.08
P07951-1	Tropomyosin beta chain [OS=Homo sapiens]	12.057	13	3	32	1	339	38.81
P23526-1	Adenosylhomocysteinase [OS=Homo sapiens]	12.012	11	4	19	4	185	23.37
Q13263	Transcription intermediary factor 1-beta [OS=Homo sapiens]	11.677	5	2	4	2	51	2.56
Q562R1	Beta-actin-like protein 2 [OS=Homo sapiens]	10.831	16	5	32	1	178	20.75
O14979-1	Heterogeneous nuclear ribonucleoprotein D-like [OS=Homo sapiens]	10.828	10	5	26	2	162	21.84
P0C0S5	Histone H2A.Z [OS=Homo sapiens]	10.112	23	3	112	1	877	107.76
Q92841	Probable ATP-dependent RNA helicase DDX17 [OS=Homo sapiens]	9.935	4	3	12	2	188	20.25
P07195	L-lactate dehydrogenase B chain [OS=Homo sapiens]	9.867	13	4	18	2	176	17.45
P00338-1	L-lactate dehydrogenase A chain [OS=Homo sapiens]	9.832	11	4	18	2	124	14.65
Q9HB71	Calcyclin-binding protein [OS=Homo sapiens]	9.789	11	2	22	2	388	3.35
P49368-1	T-complex protein 1 subunit gamma [OS=Homo sapiens]	9.395	7	3	22	3	373	31.2
P04083	annexin A1 [OS=Homo sapiens]	9.322	12	3	14	3	129	16.74
Q15654-1	thyroid receptor-interacting protein 6 [OS=Homo sapiens]	8.906	4	1	8	1	74	16.35
P50990	T-complex protein 1 subunit theta [OS=Homo sapiens]	8.22	4	2	18	2	256	26.43
P78371-1	T-complex protein 1 subunit beta [OS=Homo sapiens]	7.913	4	2	13	2	136	8
P46781	40S ribosomal protein S9 [OS=Homo sapiens]	7.904	24	4	31	4	250	27.2
P15880	40S ribosomal protein S2 [OS=Homo sapiens]	7.258	12	3	28	3	192	32.98
P42704	Leucine-rich PPR motif-containing protein, mitochondrial [OS=Homo	7.156	2	2	4	2	51	5.8
P62701	40S ribosomal protein S4, X isoform [OS=Homo sapiens]	7.128	13	3	17	3	153	17.09
P62258-1	14-3-3 protein epsilon [OS=Homo sapiens]	7.041	5	2	35	1	164	22.93
Q13838-1	spliceosome RNA helicase DDX39B [OS=Homo sapiens]	7.025	6	3	8	3	89	5.06
Q9UN86	Ras GTPase-activating protein-binding protein 2 [OS=Homo sapiens]	6.993	6	2	13	1	40	2.46
P18124	60S ribosomal protein L7 [OS=Homo sapiens]	6.914	10	3	15	3	116	17.16
P17987	T-complex protein 1 subunit alpha [OS=Homo sapiens]	6.893	5	3	12	3	104	15.53
P62913	60S ribosomal protein L11 [OS=Homo sapiens]	6.659	15	2	14	2	187	15.16
P63173	60s ribosomal protein l38 [OS=Homo sapiens]	6.591	19	1	6	1	100	10.3
P52292	Importin subunit alpha-1 [OS=Homo sapiens]	6.23	4	2	10	2	227	0
Q01130	serine/arginine-rich splicing factor 2 [OS=Homo sapiens]	6.135	8	3	32	3	396	38.66
P11021	78 kDa glucose-regulated protein [OS=Homo sapiens]	6.087	4	2	12	1	142	17.17
Q08211	Atp-dependent rna helicase a [OS=Homo sapiens]	6.038	3	3	12	3	80	9.26
P62857	40S ribosomal protein S28 [OS=Homo sapiens]	5.929	28	1	6	1	46	9.87
P08708	40S ribosomal protein S17 [OS=Homo sapiens]	5.806	9	2	6	2	47	2.53
P62316	Small nuclear ribonucleoprotein Sm D2 [OS=Homo sapiens]	5.769	14	1	6	1	45	12.26
O75494-1	Serine/arginine-rich splicing factor 10 [OS=Homo sapiens]	5.742	5	1	14	1	162	20.72
Q15366-1	Poly(rC)-binding protein 2 [OS=Homo sapiens]	5.692	7	2	24	2	334	29.38
P17844	probable ATP-dependent RNA helicase DDX5 [OS=Homo sapiens]	5.383	4	3	6	2	31	4.71
Q07666	KH domain-containing, RNA-binding, signal transduction-associated protein 1 [OS=Homo sapiens]	5.333	4	2	12	2	58	10.95
P35268	60S ribosomal protein L22 [OS=Homo sapiens]	5.248	20	2	6	2	28	7.36
P99999	cytochrome c [OS=Homo sapiens]	5.105	18	2	4	2	35	2.13
P40227-1	T-complex protein 1 subunit zeta [OS=Homo sapiens]	5.087	2	2	6	2	51	6.09
Q13347	Eukaryotic translation initiation factor 3 subunit I [OS=Homo sapiens]	5.074	4	1	2	1	42	4.39
O43169	Cytochrome b5 type B [OS=Homo sapiens]	5.007	9	1	2	1	52	4.21
O00422	Histone deacetylase complex subunit SAP18 [OS=Homo sapiens]	4.988	12	2	6	2	27	5.17
P26599	Polypyrimidine tract-binding protein 1 [OS=Homo sapiens]	4.982	7	2	4	2	54	6.69
Q04837	Single-stranded DNA-binding protein, mitochondrial [OS=Homo sapiens]	4.883	7	1	8	1	60	12.34
P84090	Enhancer of rudimentary homolog [OS=Homo sapiens]	4.681	15	1	8	1	130	
Q9Y2W1	Thyroid hormone receptor-associated protein 3 [OS=Homo sapiens]	4.627	3	3	13	3	19	13.17
P15407	Fos-related antigen 1 [OS=Homo sapiens]	4.577	5	1	4	1	17	2.37
Q9NYF8-1	Bcl-2-associated transcription factor 1 [OS=Homo sapiens]	4.57	2	2	3	2	44	2.8
O43707	Alpha-actinin-4 [OS=Homo sapiens]	4.545	1	1	4	1	81	6.29
P08621-1	U1 small nuclear ribonucleoprotein 70 kDa [OS=Homo sapiens]	4.434	2	1	8	1	48	9.41
O75937	DnaJ homolog subfamily C member 8 [OS=Homo sapiens]	4.427	8	1	2	1	0	0
O75152	Zinc finger CCCH domain-containing protein 11A [OS=Homo sapiens]	4.427	3	1	6	1	70	2.82
Q8WWI1	LIM domain only protein 7 [OS=Homo sapiens]	4.366	2	2	17	2	231	23.86
P49773	Histidine triad nucleotide-binding protein 1 [OS=Homo sapiens]	4.232	11	1	6	1	21	5.39
P12270	Nucleoprotein TPR [OS=Homo sapiens]	4.214	1	2	7	2	39	0

P62910	60S ribosomal protein L32 [OS=Homo sapiens]	4.193	10	1	6	1	102	9.29
Q15424-1	Scaffold attachment factor B1 [OS=Homo sapiens]	4.009	1	1	2	1	41	2.98
Q13404	Ubiquitin-conjugating enzyme E2 variant 1 [OS=Homo sapiens]	3.967	8	1	4	1	97	
Q9Y3Y2	Chromatin target of PRMT1 protein [OS=Homo sapiens]	3.96	5	1	2	1	57	3.42
Q9Y266	nuclear migration protein nudC [OS=Homo sapiens]	3.938	5	1	6	1	48	9.53
Q9UQ35	serine/arginine repetitive matrix protein 2 [OS=Homo sapiens]	3.899	1	2	9	2	35	5.31
P40926	Malate dehydrogenase, mitochondrial [OS=Homo sapiens]	3.887	7	1	2	1	23	2.53
P25398	40S ribosomal protein S12 [OS=Homo sapiens]	3.877	8	1	6	1	71	6.97
Q99623	Prohibitin-2 [OS=Homo sapiens]	3.733	4	1	6	1	74	3.94
P62263	40S ribosomal protein S14 [OS=Homo sapiens]	3.681	9	1	2	1	37	2.83
P04792	Heat shock protein beta-1 [OS=Homo sapiens]	3.633	5	1	6	1	94	8.01
P31153	S-adenosylmethionine synthase isoform type-2 [OS=Homo sapiens]	3.628	7	2	4	2	27	2.44
P30101	Protein disulfide-isomerase A3 [OS=Homo sapiens]	3.566	2	1	8	1	125	9.93
P62906	60S ribosomal protein L10A [OS=Homo sapiens]	3.558	6	1	3	1	20	5.04
P62333	26S proteasome regulatory subunit 10B [OS=Homo sapiens]	3.353	4	1	2	1	0	3.45
Q86U42-1	polyadenylate-binding protein 2 [OS=Homo sapiens]	3.331	7	1	2	1	0	
P50914	60S ribosomal protein L14 [OS=Homo sapiens]	3.208	6	1	6	1	0	7.6
Q16881-1	Thioredoxin reductase 1, cytoplasmic [OS=Homo sapiens]	3.198	2	1	6	1	33	7.93
P48643	T-complex protein 1 subunit epsilon [OS=Homo sapiens]	3.176	2	1	4	1	84	5.77
P84098	60S ribosomal protein L19 [OS=Homo sapiens]	3.168	5	1	4	1	55	6.58
P50991	T-complex protein 1 subunit delta [OS=Homo sapiens]	3.097	3	2	8	2	29	5.38
Q9Y3U8	60S ribosomal protein L36 [OS=Homo sapiens]	3.07	30	2	6	2	21	5.78
P22234	multifunctional protein ADE2 [OS=Homo sapiens]	3.021	3	1	5	1	88	
P35232	Prohibitin [OS=Homo sapiens]	2.973	4	1	4	1	0	2.33
P29401	Transketolase [OS=Homo sapiens]	2.969	2	1	6	1	100	7.52
Q01081	Splicing factor U2AF 35 kDa subunit [OS=Homo sapiens]	2.936	6	1	2	1	38	3.27
Q13185	chromobox protein homolog 3 [OS=Homo sapiens]	2.91	8	1	4	1	0	5.24
P61353	60S ribosomal protein L27 [OS=Homo sapiens]	2.8	6	1	4	1	70	3.53
P23588	eukaryotic translation initiation factor 4B [OS=Homo sapiens]	2.747	3	2	8	2	13	7.49
P00505	Aspartate aminotransferase, mitochondrial [OS=Homo sapiens]	2.604	2	1	6	1	49	1.66
P43686	26S proteasome regulatory subunit 6B [OS=Homo sapiens]	2.555	3	1	6	1	0	7.85
Q8WXF1	Paraspeckle component 1 [OS=Homo sapiens]	2.451	2	1	4	1	19	6.23
O15143	Actin-related protein 2/3 complex subunit 1B [OS=Homo sapiens]	2.435	3	1	4	1	50	4.85
O75821	Eukaryotic translation initiation factor 3 subunit G [OS=Homo sapiens]	2.385	3	1	2	1	25	2.19
P20700	Lamin-B1 [OS=Homo sapiens]	2.35	2	1	4	1	29	4.5
P30838	Aldehyde dehydrogenase, dimeric NADP-preferring [OS=Homo sapiens]	2.24	3	1	2	1	36	1.71
Q7KZF4	staphylococcal nuclease domain-containing protein 1 [OS=Homo sapiens]	2.223	1	1	2	1	27	1.99
Q9GZT3-1	SRA stem-loop-interacting RNA-binding protein, mitochondrial [OS=Homo sapiens]	2.203	9	1	2	1	20	2.44
Q9UMS4	Pre-mRNA-processing factor 19 [OS=Homo sapiens]	2.182	4	1	4	1	0	2.84
P39019	40S ribosomal protein S19 [OS=Homo sapiens]	2.151	14	2	6	2	24	4.81
Q8N1F7-1	Nuclear pore complex protein Nup93 [OS=Homo sapiens]	2.142	1	1	2	1	39	3.04
P13010	X-ray repair cross-complementing protein 5 [OS=Homo sapiens]	2.091	1	1	6	1	63	2.01
P62888	60S ribosomal protein L30 [OS=Homo sapiens]	2.088	10	1	6	1	110	8.72
P55209	Nucleosome assembly protein 1-like 1 [OS=Homo sapiens]	2.074	3	1	2	1	17	2.22
Q99426	tubulin-folding cofactor B [OS=Homo sapiens]	1.999	4	1	2	1	36	2.18
P62633-1	Cellular nucleic acid-binding protein [OS=Homo sapiens]	1.994	7	1	2	1	22	2.03
P12429	annexin A3 [OS=Homo sapiens]	1.941	2	1	1	1	22	
Q14247-1	Src substrate cortactin [OS=Homo sapiens]	1.86	3	1	2	1	17	3.4
Q2TAY7	WD40 repeat-containing protein SMU1 [OS=Homo sapiens]	1.845	2	1	2	1	15	2.22
O75367-1	Core histone macro-H2A.1 [OS=Homo sapiens]	1.788	2	1	4	1	0	1.74
Q9NZM1	Myoferlin [OS=Homo sapiens]	1.787	1	1	2	1	0	2.05
P12004	proliferating cell nuclear antigen [OS=Homo sapiens]	1.784	3	1	2	1	0	1.7
Q15181	Inorganic pyrophosphatase [OS=Homo sapiens]	1.775	3	1	6	1	58	6.01
Q13126-1	S-methyl-5'-thioadenosine phosphorylase [OS=Homo sapiens]	1.723	4	1	2	1	13	2.18
Q13435	Splicing factor 3b subunit 2 [OS=Homo sapiens]	1.699	1	1	5	1	38	7.12
Q14004-1	Cyclin-dependent kinase 13 [OS=Homo sapiens]	1.671	1	1	1	1		1.72
O00571	ATP-dependent RNA helicase DDX3X [OS=Homo sapiens]	1.663	2	1	2	1	28	1.81
Q9HAV7	GrpE protein homolog 1, mitochondrial [OS=Homo sapiens]	1.609	5	1	9	1	0	2.05
O43396	Thioredoxin-like protein 1 [OS=Homo sapiens]	1.583	2	1	8	1	100	1.83
Q01469	Fatty acid-binding protein, epidermal [OS=Homo sapiens]	1.558	7	1	4	1	31	
P27816-1	Microtubule-associated protein 4 [OS=Homo sapiens]	1.55	1	1	2	1	0	1.85
P31150	Rab GDP dissociation inhibitor alpha [OS=Homo sapiens]	1.49	2	1	2	1	45	0
Q00059	Transcription factor A, mitochondrial [OS=Homo sapiens]	1.41	6	1	4	1	0	0
O95644-1	Nuclear factor of activated T-cells, cytoplasmic 1 [OS=Homo sapiens]	1.387	1	1	2	1		4.82
Q9NX63	MICOS complex subunit MIC19 [OS=Homo sapiens]	1.21	4	1	2	1	13	0
P61513	60S ribosomal protein L37a [OS=Homo sapiens]	1.118	10	1	1	1		0
Q9Y230	RuvB-like 2 [OS=Homo sapiens]	1.095	3	1	2	1	0	2.1
Q14847	LIM and SH3 domain protein 1 [OS=Homo sapiens]	1.037	5	1	4	1	18	8.16
Q15643	Thyroid receptor-interacting protein 11 [OS=Homo sapiens]	1.023	2	1	4	1		3.56
P32969	60S ribosomal protein L9 [OS=Homo sapiens]	0.998	4	1	3	1	39	0
P11586	C-1-tetrahydrofolate synthase, cytoplasmic [OS=Homo sapiens]	0.929	1	1	2	1	35	1.89
Q00688	peptidyl-prolyl cis-trans isomerase FKBP3 [OS=Homo sapiens]	0.927	4	1	2	1	0	0

Supplementary Table S8. RNAs identified by ChIRP-RNA-seq analysis

Gene ID	Gene name	Category
ENSG00000240541.3	TM4SF1-AS1	lncRNA
ENSG00000283944.1	MIR4709	miRNA
ENSG00000237875.1	AL353691.1	processed_pseudogene
ENSG00000210191.1	MT-TL2	Mt_tRNA
ENSG00000278523.1	Y_RNA	misc_RNA
ENSG00000227063.5	RPL41P1	processed_pseudogene
ENSG00000274197.1	U4	snRNA
ENSG00000273744.1	U4	snRNA
ENSG00000284541.1	MIR4517	miRNA
ENSG00000066044.15	ELAVL1	protein_coding
ENSG00000207129.1	RNA5SP187	rRNA_pseudogene
ENSG00000207032.1	Y_RNA	misc_RNA
ENSG00000276664.1	AC004542.5	misc_RNA
ENSG00000235174.1	RPL39P3	processed_pseudogene
ENSG00000206738.1	Y_RNA	misc_RNA
ENSG00000277599.1	AP000944.2	misc_RNA
ENSG00000236439.4	AC099336.2	processed_pseudogene
ENSG00000199332.1	Y_RNA	misc_RNA
ENSG00000265724.1	MIR4284	miRNA
ENSG00000172428.11	COPS9	protein_coding
ENSG00000262526.2	AC120057.2	protein_coding
ENSG00000284154.1	MIR3605	miRNA
ENSG00000207194.1	RNU6-1026P	snRNA
ENSG00000238516.1	Y_RNA	misc_RNA
ENSG00000252082.1	RNU6-547P	snRNA
ENSG00000202470.1	Y_RNA	misc_RNA
ENSG00000199753.1	SNORD104	snoRNA
ENSG00000167608.12	TMC4	protein_coding
ENSG00000207198.1	RNU6-1195P	snRNA
ENSG00000209482.1	SNORD83A	snoRNA
ENSG00000206799.1	SNORA32	snoRNA
ENSG00000104219.13	ZDHHC2	protein_coding
ENSG00000272707.1	AC046143.2	lncRNA
ENSG00000252269.1	RNU4ATAC12P	snRNA
ENSG00000273542.2	H4C12	protein_coding
ENSG00000204196.5	RPL12P16	processed_pseudogene
ENSG00000126790.12	L3HYPDH	protein_coding
ENSG00000199301.1	RNU6-208P	snRNA
ENSG00000200250.1	RNU6-1147P	snRNA
ENSG00000207162.1	RNU6-549P	snRNA
ENSG00000143156.14	NME7	protein_coding
ENSG00000184939.16	ZFP90	protein_coding
ENSG00000275908.1	AC004381.2	snRNA
ENSG00000222604.2	AC007956.1	snoRNA
ENSG00000226744.1	AC005326.1	processed_pseudogene
ENSG00000124299.15	PEPD	protein_coding
ENSG00000151835.16	SACS	protein_coding
ENSG00000271598.1	AC008739.3	processed_pseudogene
ENSG00000206847.1	Y_RNA	misc_RNA
ENSG00000135127.11	BICDL1	protein_coding
ENSG00000274760.1	AL049766.1	misc_RNA
ENSG00000284038.1	MIR10A	miRNA
ENSG00000160606.11	TLCD1	protein_coding
ENSG00000206865.1	Y_RNA	misc_RNA
ENSG00000201221.1	RNU4-40P	snRNA

ENSG00000244270.1	RPL32P29	processed_pseudogene
ENSG00000243621.1	AC003989.2	processed_pseudogene
ENSG00000206881.1	RNU6-190P	snRNA
ENSG00000252283.1	Vault	misc_RNA
ENSG00000221264.1	MIR1284	miRNA
ENSG00000199551.1	RNU6-545P	snRNA
ENSG00000239129.1	AC104982.1	snoRNA
ENSG00000251785.1	RNA5SP20	rRNA_pseudogene
ENSG00000201900.1	RNY1P13	misc_RNA
ENSG00000268836.1	Z69706.1	lncRNA
ENSG00000200385.1	AL136296.1	snoRNA
ENSG00000278526.1	AC002542.5	misc_RNA
ENSG00000254564.1	AC100767.1	processed_pseudogene
ENSG00000267755.1	AC005329.2	lncRNA
ENSG00000206620.1	SNORD45C	snoRNA
ENSG00000242612.7	DECR2	protein_coding
ENSG00000202119.1	RNU6-302P	snRNA
ENSG00000206583.1	RNU6-1292P	snRNA
ENSG00000206880.1	RNU6-1310P	snRNA
ENSG00000207362.1	RNU6-422P	snRNA
ENSG00000156755.10	IGKV1OR-2	IG_V_pseudogene
ENSG00000263859.1	AC145207.6	lncRNA
ENSG00000100490.9	CDKL1	protein_coding
ENSG00000269621.1	AL589765.7	lncRNA
ENSG00000231992.1	AC092802.3	lncRNA
ENSG00000148444.16	COMMD3	protein_coding
ENSG00000277290.1	AC136475.10	processed_pseudogene
ENSG00000259715.1	AC022087.1	lncRNA
ENSG00000284435.1	MIR4691	miRNA
ENSG00000033011.13	ALG1	protein_coding
ENSG00000201114.1	Y_RNA	misc_RNA
ENSG00000184363.10	PKP3	protein_coding
ENSG00000259187.1	AC122108.1	lncRNA
ENSG00000212664.5	AC064799.1	processed_pseudogene
ENSG00000283418.1	U6	snRNA
ENSG00000230953.2	AC099677.1	processed_pseudogene
ENSG00000261061.1	AC092718.4	lncRNA
ENSG00000253051.1	SNORA31B	snoRNA
ENSG00000252824.1	AC005086.1	snoRNA
ENSG00000231153.1	AC002429.1	processed_pseudogene
ENSG00000271870.1	AC024060.2	lncRNA
ENSG00000205609.13	EIF3CL	protein_coding
ENSG00000270170.2	NCBP2AS2	protein_coding
ENSG00000200138.1	RNY1P10	misc_RNA
ENSG00000201217.1	Y_RNA	misc_RNA
ENSG00000252874.2	Y_RNA	misc_RNA
ENSG00000199631.1	SNORD33	snoRNA
ENSG00000239602.1	RPL35AP16	processed_pseudogene
ENSG00000264349.1	MIR4258	miRNA
ENSG00000243056.2	EIF4EBP3	protein_coding
ENSG00000253048.1	RNU4-60P	snRNA
ENSG00000163728.11	TTC14	protein_coding
ENSG00000163319.11	MRPS18C	protein_coding
ENSG00000103356.18	EARS2	protein_coding
ENSG00000137831.15	UACA	protein_coding
ENSG00000202498.1	SNORD116	snoRNA
ENSG00000206811.1	SNORA10	snoRNA
ENSG00000241990.5	PRR34-AS1	lncRNA

ENSG00000133433.11	GSTT2B	protein_coding
ENSG00000146826.17	MAP11	protein_coding
ENSG00000164978.18	NUDT2	protein_coding
ENSG00000163156.12	SCNM1	protein_coding
ENSG00000166347.19	CYB5A	protein_coding
ENSG00000158716.9	DUSP23	protein_coding
ENSG00000243260.3	RN7SL558P	misc_RNA
ENSG00000206977.1	AL365205.2	snoRNA
ENSG00000212443.1	SNORA53	snoRNA
ENSG00000277184.1	SNORA9	snoRNA
ENSG00000184924.5	PTRHD1	protein_coding
ENSG00000202363.1	SNORA62	snoRNA
ENSG00000079482.13	OPHN1	protein_coding
ENSG00000119203.14	CPSF3	protein_coding
ENSG00000175387.16	SMAD2	protein_coding
ENSG00000025796.14	SEC63	protein_coding
ENSG00000272418.1	AC090607.4	lncRNA
ENSG00000165113.13	GKAP1	protein_coding
ENSG00000169607.13	CKAP2L	protein_coding
ENSG00000286048.1	AC008966.3	lncRNA
ENSG00000179029.15	TMEM107	protein_coding
ENSG00000118922.17	KLF12	protein_coding
ENSG00000253676.1	TAGLN2P1	processed_pseudogene
ENSG00000265415.1	AC099850.3	lncRNA
ENSG00000248256.1	OCIAD1-AS1	lncRNA
ENSG00000258917.1	ZMYND19P1	processed_pseudogene
ENSG00000175701.10	MTLN	protein_coding
ENSG00000201863.1	AC098591.1	snoRNA
ENSG00000267198.1	AC091132.4	lncRNA
ENSG00000103490.14	PYCARD	protein_coding
ENSG00000212457.1	RNU6-644P	snRNA
ENSG00000277978.1	AC010542.5	lncRNA
ENSG00000229257.2	AL807752.2	lncRNA
ENSG00000240723.3	RN7SL382P	misc_RNA
ENSG00000264937.1	AC100830.2	lncRNA
ENSG00000264810.1	MIR4441	miRNA
ENSG00000125144.14	MT1G	protein_coding
ENSG00000200959.1	SNORA74A	snoRNA
ENSG00000143374.17	TARS2	protein_coding
ENSG00000256448.5	AP000763.3	lncRNA
ENSG00000237854.3	LINC00674	transcribed_unprocessed_pseudogene
ENSG00000244391.3	RN7SL330P	misc_RNA
ENSG00000121064.13	SCPEP1	protein_coding
ENSG00000196668.4	LINC00173	lncRNA
ENSG00000183527.12	PSMG1	protein_coding
ENSG00000133250.14	ZNF414	protein_coding
ENSG00000170006.12	TMEM154	protein_coding
ENSG00000221340.1	RNU6ATAC18P	snRNA
ENSG00000251703.1	RNU6-998P	snRNA
ENSG00000176383.9	B3GNT4	protein_coding
ENSG00000202071.1	Y_RNA	misc_RNA
ENSG00000274093.1	AC009032.1	lncRNA
ENSG00000177058.12	SLC38A9	protein_coding
ENSG00000234268.1	AP000936.3	processed_pseudogene
ENSG00000267203.1	SNRPGP4	processed_pseudogene
ENSG00000140563.15	MCTP2	protein_coding
ENSG00000164494.12	PDSS2	protein_coding
ENSG00000252994.1	RNU6-1231P	snRNA

ENSG00000272831.1	AC027644.3	lncRNA
ENSG00000243015.2	RN7SL737P	misc_RNA
ENSG00000135469.14	COQ10A	protein_coding
ENSG00000250917.1	AL035458.2	lncRNA
ENSG00000183943.6	PRKX	protein_coding
ENSG00000228292.1	AL512604.1	processed_pseudogene
ENSG00000226266.6	AC009961.1	lncRNA
ENSG00000115963.13	RND3	protein_coding
ENSG00000223313.1	RNU6-516P	snRNA
ENSG00000228830.1	AL160408.2	lncRNA
ENSG00000223305.1	RN7SKP30	misc_RNA
ENSG00000158163.15	DZIP1L	protein_coding
ENSG00000148700.15	ADD3	protein_coding
ENSG00000214279.13	SCART1	protein_coding
ENSG00000251754.1	RNU6-999P	snRNA
ENSG00000221955.11	SLC12A8	protein_coding
ENSG00000260335.1	AC133555.4	unprocessed_pseudogene
ENSG00000224597.10	SVIL-AS1	transcribed_unprocessed_pseudogene
ENSG00000156983.16	BRPF1	protein_coding
ENSG00000006025.12	OSBPL7	protein_coding
ENSG00000256826.1	ATP5MFP4	processed_pseudogene
ENSG00000163960.12	UBXN7	protein_coding
ENSG00000246705.4	H2AJ	protein_coding
ENSG00000066379.15	POLR1H	protein_coding
ENSG00000207820.1	MIR545	miRNA
ENSG00000252414.1	RNU6-100P	snRNA
ENSG00000252554.1	RNU6-861P	snRNA
ENSG00000206603.1	SNORA22B	snoRNA
ENSG00000254829.1	AP003032.2	lncRNA
ENSG00000199700.1	RNU6-223P	snRNA
ENSG00000201162.1	RNU6-454P	snRNA
ENSG00000201709.1	RNU6-686P	snRNA
ENSG00000202445.1	RNU6-669P	snRNA
ENSG00000206631.1	RNU6-657P	snRNA
ENSG00000222051.1	RNU6-1165P	snRNA
ENSG00000252941.1	RNA5SP340	rRNA_pseudogene
ENSG00000201076.1	RNU4-51P	snRNA
ENSG00000286811.1	AL353138.1	lncRNA
ENSG00000154124.5	OTULIN	protein_coding
ENSG00000250322.2	AC026402.1	processed_pseudogene
ENSG00000100364.19	KIAA0930	protein_coding
ENSG00000222344.1	RNU6-613P	snRNA
ENSG00000233184.7	AC093157.1	lncRNA
ENSG00000102125.16	TAZ	protein_coding
ENSG00000104267.10	CA2	protein_coding
ENSG00000206650.1	SNORA70G	snoRNA
ENSG00000223345.3	H2BP1	transcribed_unprocessed_pseudogene
ENSG00000014824.14	SLC30A9	protein_coding
ENSG00000275607.1	AC135507.2	processed_pseudogene
ENSG00000268521.1	VN1R83P	unprocessed_pseudogene
ENSG00000254665.1	AC091053.1	lncRNA
ENSG00000201035.1	RNA5SP469	rRNA_pseudogene
ENSG00000282542.1	AC008993.1	TEC
ENSG00000145901.16	TNIP1	protein_coding
ENSG00000104213.13	PDGFRL	protein_coding
ENSG00000197603.15	CPLANE1	protein_coding
ENSG00000172748.14	ZNF596	protein_coding
ENSG00000235954.7	TTC28-AS1	lncRNA

ENSG00000207595.1	MIR181A2	miRNA
ENSG00000280120.1	AC073857.1	TEC
ENSG00000257303.2	AC073896.2	lncRNA
ENSG00000200418.1	SNORA63B	snoRNA
ENSG00000269427.1	AC024075.3	lncRNA
ENSG00000163322.14	ABRAXAS1	protein_coding
ENSG00000260708.1	AL118516.1	lncRNA
ENSG00000280242.1	AL450226.2	TEC
ENSG00000271626.1	H3P42	processed_pseudogene
ENSG00000165934.13	CPSF2	protein_coding
ENSG00000211643.2	IGLV5-52	IG_V_gene
ENSG00000215630.6	GUSBP9	unprocessed_pseudogene
ENSG00000267751.5	AC009005.1	lncRNA
ENSG00000215283.3	HMGB3P24	processed_pseudogene
ENSG00000259399.1	TGIF2-RAB5IF	protein_coding
ENSG00000259001.3	AL355075.4	lncRNA
ENSG00000263647.1	BPTFP1	unprocessed_pseudogene
ENSG00000230955.1	AL929472.2	lncRNA
ENSG00000182165.18	TP53TG1	lncRNA
ENSG00000152818.18	UTRN	protein_coding
ENSG00000160446.19	ZDHHC12	protein_coding
ENSG00000229298.1	TUBB8P1	processed_pseudogene
ENSG00000222432.1	Y_RNA	misc_RNA
ENSG00000143314.12	MRPL24	protein_coding
ENSG00000231993.1	EP300-AS1	lncRNA
ENSG00000130772.14	MED18	protein_coding
ENSG00000230902.1	FAM204CP	processed_pseudogene
ENSG00000259112.2	NDUFC2-KCTD14	protein_coding
ENSG00000145217.14	SLC26A1	protein_coding
ENSG00000164506.14	STXBP5	protein_coding
ENSG00000169371.14	SNUPN	protein_coding
ENSG00000103995.14	CEP152	protein_coding
ENSG00000260304.1	AC009088.2	lncRNA
ENSG00000200842.1	Y_RNA	misc_RNA
ENSG00000223450.1	AL590632.1	processed_pseudogene
ENSG00000239453.1	SIDT1-AS1	lncRNA
ENSG00000213963.6	AC019080.1	lncRNA
ENSG00000135250.17	SRPK2	protein_coding
ENSG00000118997.14	DNAH7	protein_coding
ENSG00000261717.5	AC009163.5	protein_coding
ENSG00000114023.15	FAM162A	protein_coding
ENSG00000251062.1	AC025459.1	processed_pseudogene
ENSG00000279394.1	AC015871.4	TEC
ENSG00000265315.1	RN7SL199P	misc_RNA
ENSG00000265411.1	RN7SL656P	misc_RNA
ENSG00000235058.1	ZMYND10-AS1	lncRNA
ENSG00000103496.15	STX4	protein_coding
ENSG00000171132.14	PRKCE	protein_coding
ENSG00000275994.1	SNORA24	snoRNA
ENSG00000234005.3	GAPDHP22	processed_pseudogene
ENSG00000127423.11	AUNIP	protein_coding
ENSG00000133597.11	ADCK2	protein_coding
ENSG00000263756.1	AL645941.3	lncRNA
ENSG00000224463.1	RPL39P14	processed_pseudogene
ENSG00000222249.1	RNU6-262P	snRNA
ENSG00000236900.1	TIMM9P1	processed_pseudogene
ENSG00000116954.8	RRAGC	protein_coding
ENSG00000243333.3	RN7SL174P	misc_RNA

ENSG00000110169.11	HPX	protein_coding
ENSG00000265749.6	AC135178.4	lncRNA
ENSG00000286161.1	AC104066.4	lncRNA
ENSG00000027697.15	IFNGR1	protein_coding
ENSG00000224946.1	AC007312.1	processed_pseudogene
ENSG00000260246.1	AC000032.1	lncRNA
ENSG00000232430.1	RPL31P15	processed_pseudogene
ENSG00000163818.17	LZTFL1	protein_coding
ENSG00000171700.14	RGS19	protein_coding
ENSG00000018699.13	TTC27	protein_coding
ENSG00000100478.15	AP4S1	protein_coding
ENSG00000254205.1	AC009686.1	lncRNA
ENSG00000116783.15	TNNI3K	protein_coding
ENSG00000132196.15	HSD17B7	protein_coding
ENSG00000239149.1	SNORA59A	snoRNA
ENSG00000168438.15	CDC40	protein_coding
ENSG00000248161.5	AC098487.1	lncRNA
ENSG00000275185.1	AC130324.3	lncRNA
ENSG00000140057.9	AK7	protein_coding
ENSG00000242173.10	ARHGDIG	protein_coding
ENSG00000255301.1	AP002893.1	lncRNA
ENSG00000276036.1	RNA5SP440	rRNA_pseudogene
ENSG00000238186.1	AL603839.2	lncRNA
ENSG00000119514.7	GALNT12	protein_coding
ENSG00000232136.2	DUXAP7	processed_pseudogene
ENSG00000260111.1	AC012184.1	lncRNA
ENSG00000274383.1	AC103691.1	lncRNA
ENSG00000187630.17	DHRS4L2	protein_coding
ENSG00000125954.12	CHURC1-FNTB	protein_coding
ENSG00000275857.1	AC009133.4	lncRNA
ENSG00000255686.1	AC073864.1	lncRNA
ENSG00000227540.1	DNAJC9-AS1	lncRNA
ENSG00000253093.1	RNA5SP179	rRNA_pseudogene
ENSG00000248155.1	CR545473.1	unprocessed_pseudogene
ENSG00000256148.1	AP000763.2	processed_pseudogene
ENSG00000147586.10	MRPS28	protein_coding
ENSG00000269711.1	AC008763.3	protein_coding
ENSG00000232713.2	RPS12P3	processed_pseudogene
ENSG00000287779.1	AL162742.2	transcribed_unprocessed_pseudogene
ENSG00000229299.2	AL121845.1	lncRNA
ENSG00000253229.1	HIGD1AP6	processed_pseudogene
ENSG00000278099.1	RNVU1-2A	snRNA
ENSG00000275221.2	H2AC15	protein_coding
ENSG00000247809.9	NR2F2-AS1	lncRNA
ENSG00000287287.1	AL133476.1	lncRNA
ENSG00000074621.14	SLC24A1	protein_coding
ENSG00000182625.3	AC091046.1	processed_pseudogene
ENSG00000189283.10	FHIT	protein_coding
ENSG00000159915.12	ZNF233	protein_coding
ENSG00000254463.1	PPIAP41	processed_pseudogene
ENSG00000207022.1	AL162740.1	snoRNA
ENSG00000241007.1	SEPTIN7P6	processed_pseudogene
ENSG00000239465.1	AC090543.1	processed_pseudogene
ENSG00000274561.1	AC005332.3	lncRNA
ENSG00000121988.18	ZRANB3	protein_coding
ENSG00000278017.1	AC064801.1	lncRNA
ENSG00000151575.14	TEX9	protein_coding
ENSG00000221420.2	SNORA81	snoRNA

ENSG00000214628.3	NDUFB5P2	processed_pseudogene
ENSG00000168961.17	LGALS9	protein_coding
ENSG00000047230.15	CTPS2	protein_coding
ENSG00000260252.1	AC009087.1	lncRNA
ENSG00000164051.14	CCDC51	protein_coding
ENSG00000243498.2	UBA52P5	processed_pseudogene
ENSG00000144559.10	TAMM41	protein_coding
ENSG00000085760.15	MTIF2	protein_coding
ENSG00000140548.10	ZNF710	protein_coding
ENSG00000226284.1	ARPC3P1	processed_pseudogene
ENSG00000178537.10	SLC25A20	protein_coding
ENSG00000272533.1	SNORA28	snoRNA
ENSG00000237036.5	ZEB1-AS1	lncRNA
ENSG00000241217.3	RN7SL809P	misc_RNA
ENSG00000259232.2	AC105129.1	processed_pseudogene
ENSG00000262766.1	AC135050.5	lncRNA
ENSG00000287260.1	AL357075.3	lncRNA
ENSG00000244356.3	RN7SL398P	misc_RNA
ENSG00000250950.1	AC093752.2	lncRNA
ENSG00000254484.1	AP002336.1	lncRNA
ENSG00000242375.1	AL590705.3	lncRNA
ENSG00000278590.1	RN7SL113P	misc_RNA
ENSG00000253293.5	HOXA10	protein_coding
ENSG00000223989.1	AL357140.1	lncRNA
ENSG00000153094.24	BCL2L11	protein_coding
ENSG00000152942.19	RAD17	protein_coding
ENSG00000081181.8	ARG2	protein_coding
ENSG00000280317.1	AL732618.1	TEC
ENSG00000115474.7	KCNJ13	protein_coding
ENSG00000142046.15	TMEM91	protein_coding
ENSG00000272155.1	AC055822.1	lncRNA
ENSG00000206706.1	Y_RNA	misc_RNA
ENSG00000206927.1	Y_RNA	misc_RNA
ENSG00000207294.1	Y_RNA	misc_RNA
ENSG00000207425.1	Y_RNA	misc_RNA
ENSG00000089091.16	DZANK1	protein_coding
ENSG00000277143.1	AC074276.2	processed_pseudogene
ENSG00000243708.10	PLA2G4B	protein_coding
ENSG00000258531.2	BANF1P1	processed_pseudogene
ENSG00000248559.1	AC109454.2	lncRNA
ENSG00000200063.1	AC061992.1	snoRNA
ENSG00000222588.1	AL355598.1	snoRNA
ENSG00000273682.1	AC109583.3	processed_pseudogene
ENSG00000231312.7	MAP4K3-DT	lncRNA
ENSG00000262979.1	AC124319.1	lncRNA
ENSG00000101745.17	ANKRD12	protein_coding
ENSG00000259970.1	AC099668.1	lncRNA
ENSG00000273036.3	AL390726.4	transcribed_processed_pseudogene
ENSG00000166262.16	FAM227B	protein_coding
ENSG00000252355.1	RN7SKP287	misc_RNA
ENSG00000205808.7	PLPP6	protein_coding
ENSG00000255929.5	AP000943.2	lncRNA
ENSG00000246283.2	AC090510.1	lncRNA
ENSG00000154153.13	RETREG1	protein_coding
ENSG00000222057.1	RNU4-62P	snRNA
ENSG00000257957.1	QRSL1P3	processed_pseudogene
ENSG00000167874.7	TMEM88	protein_coding
ENSG00000113966.10	ARL6	protein_coding

ENSG00000151090.20	THRB	protein_coding
ENSG00000173451.7	THAP2	protein_coding
ENSG00000167311.14	ART5	protein_coding
ENSG00000211644.3	IGLV1-51	IG_V_gene
ENSG00000212607.1	SNORA3B	snoRNA
ENSG00000269951.1	AC090181.2	lncRNA
ENSG00000233114.2	AL358075.3	processed_pseudogene
ENSG00000276470.1	AL021155.2	misc_RNA
ENSG00000270553.1	AC011921.3	processed_pseudogene
ENSG00000189164.15	ZNF527	protein_coding
ENSG00000228663.1	PSMD10P1	processed_pseudogene
ENSG00000225551.1	AL157827.1	unprocessed_pseudogene
ENSG00000213145.10	CRIP1	protein_coding
ENSG00000244026.6	FAM86DP	transcribed_unprocessed_pseudogene
ENSG00000149798.5	CDC42EP2	protein_coding
ENSG00000176485.12	PLAAT3	protein_coding
ENSG00000219355.2	RPL31P52	processed_pseudogene
ENSG00000204540.11	PSORS1C1	protein_coding
ENSG00000096968.14	JAK2	protein_coding
ENSG00000123643.13	SLC36A1	protein_coding
ENSG00000167130.18	DOLPP1	protein_coding
ENSG00000198040.11	ZNF84	protein_coding
ENSG00000188910.8	GJB3	protein_coding
ENSG00000204816.5	FGF7P5	unprocessed_pseudogene
ENSG00000212396.1	RNA5SP323	rRNA_pseudogene
ENSG00000257954.1	AC125611.2	processed_pseudogene
ENSG00000265185.6	SNORD3B-1	snoRNA
ENSG00000280400.1	AC127459.3	TEC
ENSG00000234197.1	ETV5-AS1	lncRNA
ENSG00000259191.2	AC027237.1	processed_pseudogene
ENSG00000008869.12	HEATR5B	protein_coding
ENSG00000206952.3	SNORA50A	snoRNA
ENSG00000274386.5	TMEM269	protein_coding
ENSG00000110871.15	COQ5	protein_coding
ENSG00000197140.15	ADAM32	protein_coding
ENSG00000239351.1	NPM1P29	processed_pseudogene
ENSG00000197375.13	SLC22A5	protein_coding
ENSG00000279672.1	AP006621.5	TEC
ENSG00000160282.14	FTCD	protein_coding
ENSG00000276161.1	SNORA17B	snoRNA
ENSG00000206647.1	AC007240.1	snoRNA
ENSG00000275097.1	AC024940.5	lncRNA
ENSG00000224429.8	LINC00539	lncRNA
ENSG00000125388.20	GRK4	protein_coding
ENSG00000272114.1	AL136131.3	lncRNA
ENSG00000265055.2	AC145343.1	lncRNA
ENSG00000243883.3	RN7SL419P	misc_RNA
ENSG00000273568.1	AC131009.3	lncRNA
ENSG00000263335.1	AF001548.2	lncRNA
ENSG00000233460.1	RPL35AP31	processed_pseudogene
ENSG00000268047.1	AC018766.1	lncRNA
ENSG00000271064.1	AC027644.2	processed_pseudogene
ENSG00000202434.1	AC010746.1	snoRNA
ENSG00000208772.1	SNORD94	snoRNA
ENSG00000221491.2	SNORA2C	snoRNA
ENSG00000258761.1	AC116903.1	lncRNA
ENSG00000212163.6	SNORD91A	snoRNA
ENSG00000273733.1	AC011472.4	lncRNA

ENSG00000235077.1	AC073842.1	lncRNA
ENSG00000233382.7	NKAPP1	transcribed_processed_pseudogene
ENSG00000270075.1	AL162742.1	lncRNA
ENSG00000266160.2	RN7SL612P	misc_RNA
ENSG00000207165.1	SNORA70	snoRNA
ENSG00000287525.1	AL627308.3	lncRNA
ENSG00000223807.1	BOLA3P4	processed_pseudogene
ENSG00000274020.4	LINC01138	lncRNA
ENSG00000118515.11	SGK1	protein_coding
ENSG00000280136.2	AC240565.2	lncRNA
ENSG00000243896.4	OR2A7	protein_coding
ENSG00000102312.23	PORCN	protein_coding
ENSG00000264829.1	AC087749.2	lncRNA
ENSG00000277688.1	AC243585.2	lncRNA
ENSG00000262003.1	AC087392.1	lncRNA
ENSG00000253911.1	AP003467.1	lncRNA
ENSG00000287168.1	AL512625.3	lncRNA
ENSG00000163888.4	CAMK2N2	protein_coding
ENSG00000197813.6	AC011450.1	lncRNA
ENSG00000198618.5	PPIAP22	processed_pseudogene
ENSG00000229644.6	NAMPTP1	processed_pseudogene
ENSG00000238251.2	NSA2P7	processed_pseudogene
ENSG00000246889.2	AP000487.1	lncRNA
ENSG00000135932.11	CAB39	protein_coding
ENSG00000125703.15	ATG4C	protein_coding
ENSG00000232667.10	AC004862.1	lncRNA
ENSG00000138079.14	SLC3A1	protein_coding
ENSG00000261385.1	AC009088.3	lncRNA
ENSG00000187187.14	ZNF546	protein_coding
ENSG00000201806.1	RNU4-8P	snRNA
ENSG00000267379.1	AC008569.1	lncRNA
ENSG00000258695.2	AC005225.3	lncRNA
ENSG00000250271.2	AC068647.2	transcribed_unprocessed_pseudogene
ENSG00000264384.2	RN7SL431P	misc_RNA
ENSG00000087303.18	NID2	protein_coding
ENSG00000171621.14	SPSB1	protein_coding
ENSG00000277423.1	AC069234.5	lncRNA
ENSG00000110042.8	DTX4	protein_coding
ENSG00000253668.1	AC103778.1	processed_pseudogene
ENSG00000169981.11	ZNF35	protein_coding
ENSG00000258526.7	AL049828.1	lncRNA
ENSG00000266111.2	AC068025.2	lncRNA
ENSG00000102181.21	CD99L2	protein_coding
ENSG00000254483.1	SUGT1P4	unprocessed_pseudogene
ENSG00000239884.3	RN7SL608P	misc_RNA
ENSG00000144357.18	UBR3	protein_coding
ENSG00000201558.1	RNVU1-6	snRNA
ENSG00000254459.1	AP002812.2	lncRNA
ENSG00000248275.2	TRIM52-AS1	lncRNA
ENSG00000145723.17	GIN1	protein_coding
ENSG00000279718.1	SNX18P12	processed_pseudogene
ENSG00000274976.1	AC087588.1	lncRNA
ENSG00000274985.1	PTCHD3P1	unprocessed_pseudogene
ENSG00000201564.1	RN7SKP50	misc_RNA
ENSG00000279691.1	AC113410.3	TEC
ENSG00000244559.1	AC091179.2	processed_pseudogene
ENSG00000196810.5	CTBP1-DT	lncRNA
ENSG00000274628.5	AL669942.1	transcribed_unprocessed_pseudogene

ENSG00000285794.1	AL031281.1	transcribed_processed_pseudogene
ENSG00000151500.15	THYN1	protein_coding
ENSG00000271853.5	AL162258.1	lncRNA
ENSG00000269688.1	AC008982.2	lncRNA
ENSG00000131779.11	PEX11B	protein_coding
ENSG00000124171.9	PARD6B	protein_coding
ENSG00000218896.1	TUBB8P2	processed_pseudogene
ENSG00000244134.1	RPS12P20	processed_pseudogene
ENSG00000151773.13	CCDC122	protein_coding
ENSG00000235088.1	AL031667.2	processed_pseudogene
ENSG00000148848.14	ADAM12	protein_coding
ENSG00000112394.17	SLC16A10	protein_coding
ENSG00000230082.1	PRRT3-AS1	lncRNA
ENSG00000224629.1	AC004975.1	processed_pseudogene
ENSG00000281501.1	SEPSECS-AS1	lncRNA
ENSG00000253492.1	CDH12P3	unprocessed_pseudogene
ENSG00000254335.1	CDH12P1	unprocessed_pseudogene
ENSG00000111215.12	PRR4	protein_coding
ENSG00000259258.1	AC013553.1	processed_pseudogene
ENSG00000080371.6	RAB21	protein_coding
ENSG00000143375.15	CGN	protein_coding
ENSG00000212769.5	HMGN2P8	processed_pseudogene
ENSG00000281195.1	AC007878.1	lncRNA
ENSG00000213964.3	CHCHD4P4	processed_pseudogene
ENSG00000256747.1	AC009511.2	lncRNA
ENSG00000158966.16	CACHD1	protein_coding
ENSG00000280543.1	ASAP1-IT2	lncRNA
ENSG00000228779.1	Z69666.1	lncRNA
ENSG00000258068.2	AC079600.3	lncRNA
ENSG00000253908.1	AC104115.2	processed_pseudogene
ENSG00000244556.1	ODCP	processed_pseudogene
ENSG00000239257.1	RPL23AP1	transcribed_processed_pseudogene
ENSG00000270409.1	AC090950.1	transcribed_unprocessed_pseudogene
ENSG00000241204.1	AC022730.2	processed_pseudogene
ENSG00000273107.1	AL512598.1	lncRNA
ENSG00000019485.13	PRDM11	protein_coding
ENSG00000103254.10	ANTKMT	protein_coding
ENSG00000276620.1	AL591516.1	processed_pseudogene
ENSG00000249471.8	ZNF324B	protein_coding
ENSG00000143942.5	CHAC2	protein_coding
ENSG00000266469.2	AC005288.1	lncRNA
ENSG00000235663.1	SAPCD1-AS1	lncRNA
ENSG00000267128.2	RNF157-AS1	lncRNA
ENSG00000273061.1	CDC37L1-DT	lncRNA
ENSG00000226721.2	EEF1DP2	processed_pseudogene
ENSG00000267757.4	EML2-AS1	lncRNA
ENSG00000173011.12	TADA2B	protein_coding
ENSG00000275759.1	AC026367.3	lncRNA
ENSG00000254263.1	AC022973.4	lncRNA
ENSG00000275129.1	AC243994.1	processed_pseudogene
ENSG00000153721.19	CNKSR3	protein_coding
ENSG00000245571.7	FAM111A-DT	lncRNA
ENSG00000196656.7	AC004057.1	transcribed_processed_pseudogene
ENSG00000261087.1	ZNNT1	lncRNA
ENSG00000240695.1	AC117382.1	processed_pseudogene
ENSG00000241549.8	GUSBP2	transcribed_unprocessed_pseudogene
ENSG00000265337.1	AC079336.3	lncRNA
ENSG00000119686.10	FLVCR2	protein_coding

ENSG00000107014.9	RLN2	protein_coding
ENSG00000125384.7	PTGER2	protein_coding
ENSG00000277382.1	AC005837.4	lncRNA
ENSG00000078687.17	TNRC6C	protein_coding
ENSG00000277701.5	AC159540.2	lncRNA
ENSG00000252917.1	SNORA74	snoRNA
ENSG00000265073.1	AC010761.2	lncRNA
ENSG00000241211.1	IQCJ-SCHIP1-AS1	lncRNA

Supplementary Table S9. Sequences of siRNAs and shRNAs used in this study

Name	Sequence
Control siRNA	
si-TM4SF1-AS1-1	5'-CCAUCAGUUGGGAGUUGAAGA-3'
si-TM4SF1-AS1-2	5'-GCCAAGUGUCCGAGAUGCAAC-3'
si-TM4SF1-1	5'-CAAACGAUGUGCGAUGCUU-3'
si-TM4SF1-2	5'-GUGCGAUGCUUUCUUCUGUTT-3'
si-Pur-α-1	5'-CCACCUAUCGCAACUCCAUTT-3'
si-Pur-α-2	5'-GCUACUGCAGGGUGAGGAATT-3'
si-YB-1-1	5'-CCUAUGGGCGUCGACCACATT-3'
si-YB-1-2	5'-GUUCCAGUUCAAGGCAGUATT-3'
si-RACK1	5'-GAUAAGACCAUCAUCAUGUGG-3'
Control shRNA	5'-TCCTAAGGTTAAGTCGCCCTCG-3'
sh-TM4SF1AS1-1	5'-CCATCAGTTGGGAGTTGAAGA-3'
sh-TM4SF1AS1-2	5'-GCCAAGTGTCCGAGATGCAAC-3'

Supplementary Table S10. Genes analyzed by targeted sequencing

GC-related genes
APC
ARID1A
CDH1
MUC6
KRAS
PIC3CA
SMAD3
TP53

LncRNA genes
DLEU1
DLEU1-AS1
TM4SF1-AS1
NEAT1
PVT1
TCONS_00006510
TCONS_00007524
TCONS_00009689
TCONS_00010939
TCONS_00011145
TCONS_00011229
TCONS_00017242
TCONS_00021627
TCONS_00021692
TCONS_00024597
TCONS_00029727

Genes located in the antisense of lncRNA genes
ERBB2IP
MLLT4
SEMA3B
TM4SF1
UBE2L5P

Supplementary Table S11. Sequences of probes used in ChIRP analysis

Probe name	Sequence
TM4SF1-AS1-1	5'-ACAGTCAATGCCTTGTTCAA-bio-3'
TM4SF1-AS1-2	5'-TCTTTCAAAGCCTCAACCAG-bio-3'
TM4SF1-AS1-3	5'-GCCCCAGGTTGTAAATTAAA-bio-3'
TM4SF1-AS1-4	5'-CAGGGACGCTGAACACTGAA-bio-3'
TM4SF1-AS1-5	5'-CAGGAACCCTGAACAGTTTT-bio-3'
TM4SF1-AS1-6	5'-CCCAACCTTTCTGTAAACAG-bio-3'
TM4SF1-AS1-7	5'-AATCAGCTCTGAGCAAACCA-bio-3'
LacZ-1	5'-GCGTTAAAGTTGTTCTGCTT-bio-3'
LacZ-2	5'-ATGCCGTGGGTTTCAATATT-bio-3'
LacZ-3	5'-GTAGTTCAGGCAGTTCAATC-bio-3'
LacZ-4	5'-TTTTTGACACCAGACCAACT-bio-3'

bio, biotin
