# Supplementary material for: TM4SF1-AS1 inhibits apoptosis by promoting stress granule formation in cancer cells
Source: Cell Death Dis. 2023 Jul 13;14(7):424. doi: 10.1038/s41419-023-05953-3 (PMC10345132; doi:10.1038/s41419-023-05953-3)
Supplement: Supplementary file 3 — Supplementary Figures [file 41419_2023_5953_MOESM3_ESM.docx]

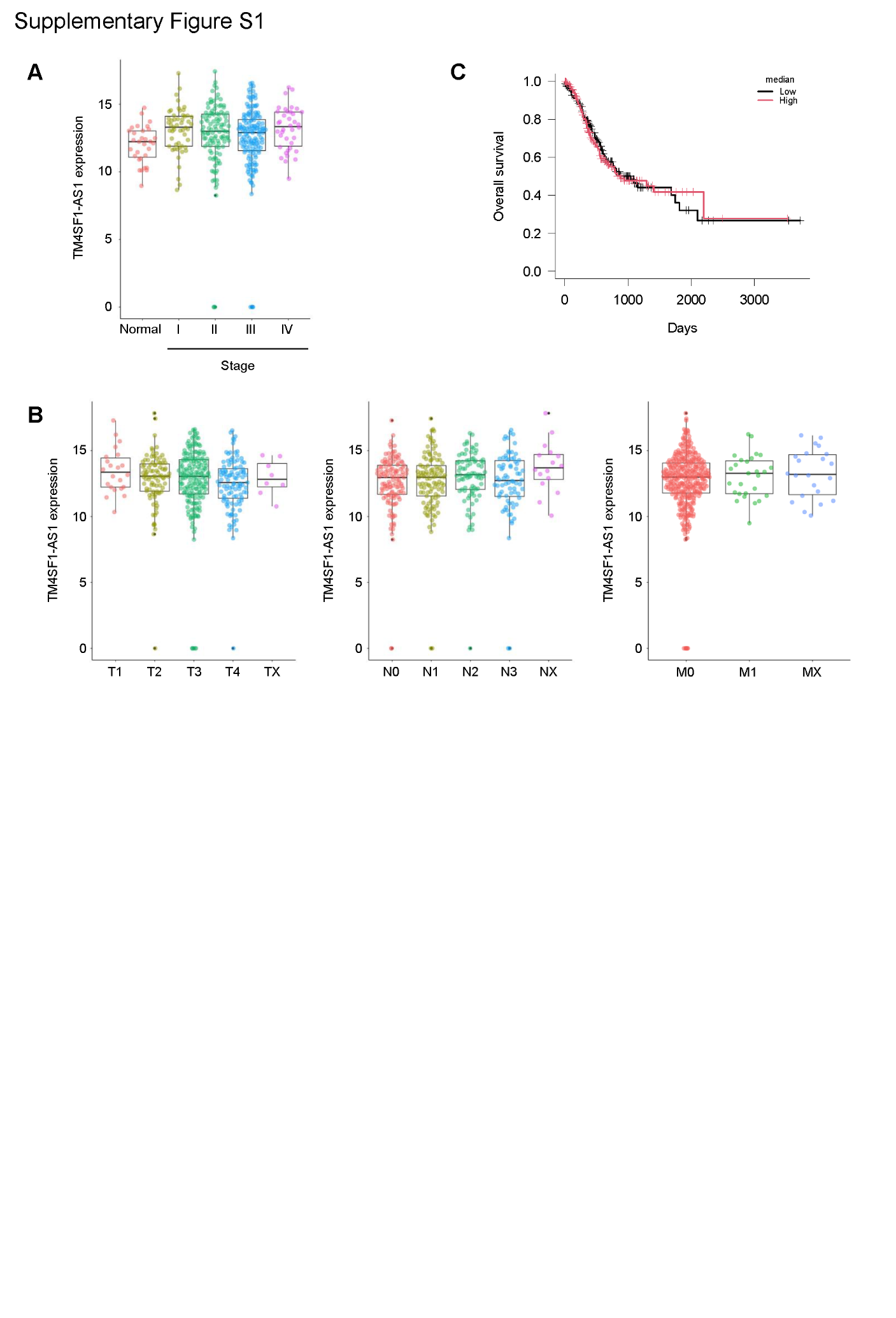


**Supplementary Figure S1**

Expression of TM4SF1-AS1 and clinical features in GC patients. (A) Levels of TM4SF1-AS1 expression in normal gastric tissues and primary GCs at the indicated stages. (B) Levels of TM4SF1‑AS1 expression in primary GCs with the indicated TNM classifications. (C) Kaplan–Meier curves showing the effect of TM4SF1-AS1 expression on overall survival in GC patients (n = 381) in The Cancer Genome Atlas (TCGA) dataset.


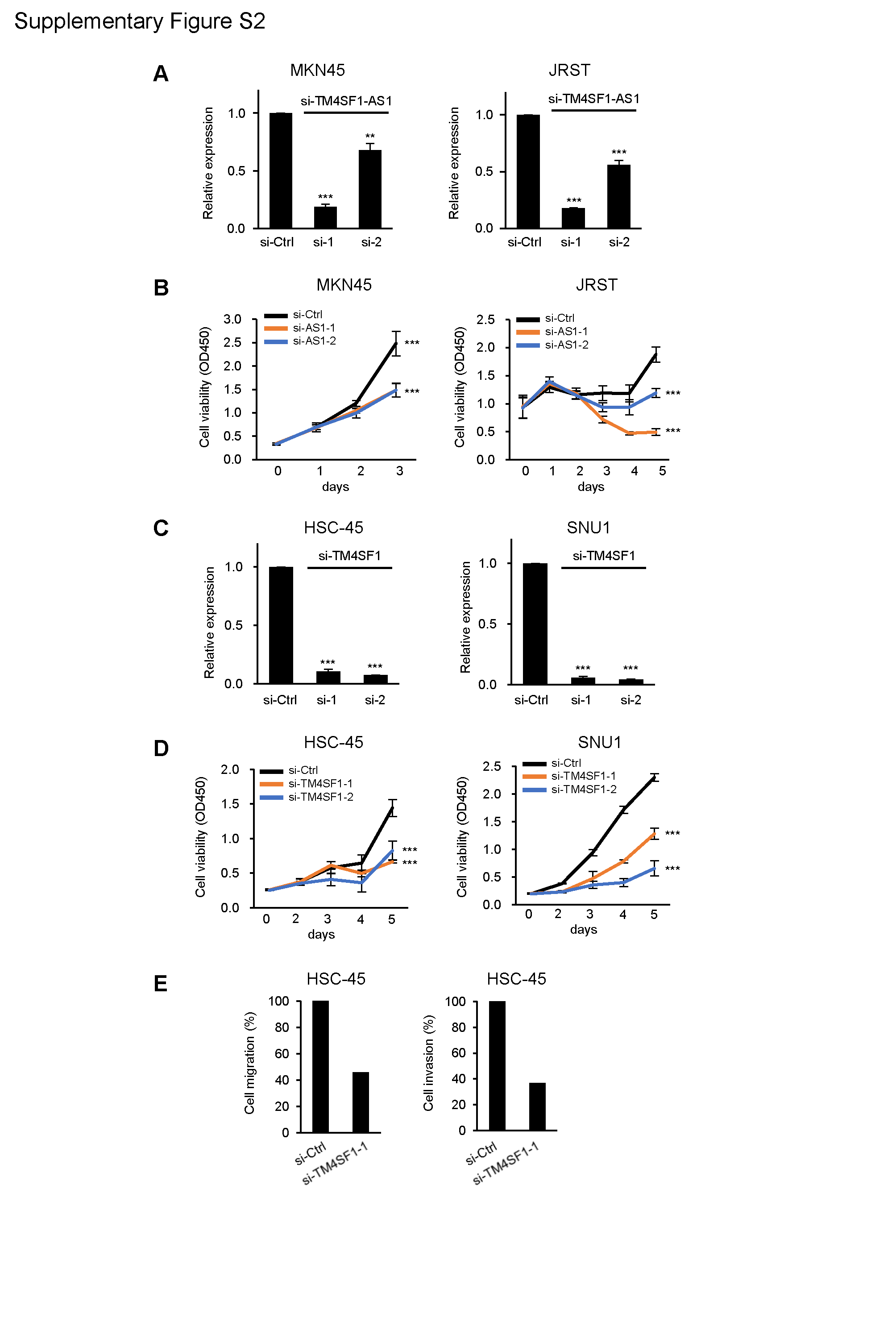


**Supplementary Figure S2**

Oncogenic functions of TM4SF1-AS1 and TM4SF1 in GC cells. (A) qRT-PCR analysis of TM4SF1‑AS1 in the indicated GC cell lines transfected with siRNAs targeting TM4SF1-AS1 (si-1 and -2) or a control siRNA (si-Ctrl). (B) Results of cell viability assays with MKN45 and JRST cells transfected with siRNAs targeting TM4SF1-AS1 or a control siRNA. (C) qRT-PCR analysis of TM4SF1 in HSC-45 and SNU1 cells transfected with a control siRNA or siRNAs targeting TM4SF1 (si-1 and -2). (D) Cell viability assays with HSC-45 and SNU1 cells transfected with siRNAs targeting TM4SF1 or a control siRNA. (E) Results of migration and invasion assays with HSC-45 cells transfected with the indicated siRNAs. Shown are means of eight replications in (B and D); error bars represent SDs. ***P*<0.0.1, ****P*<0.001.


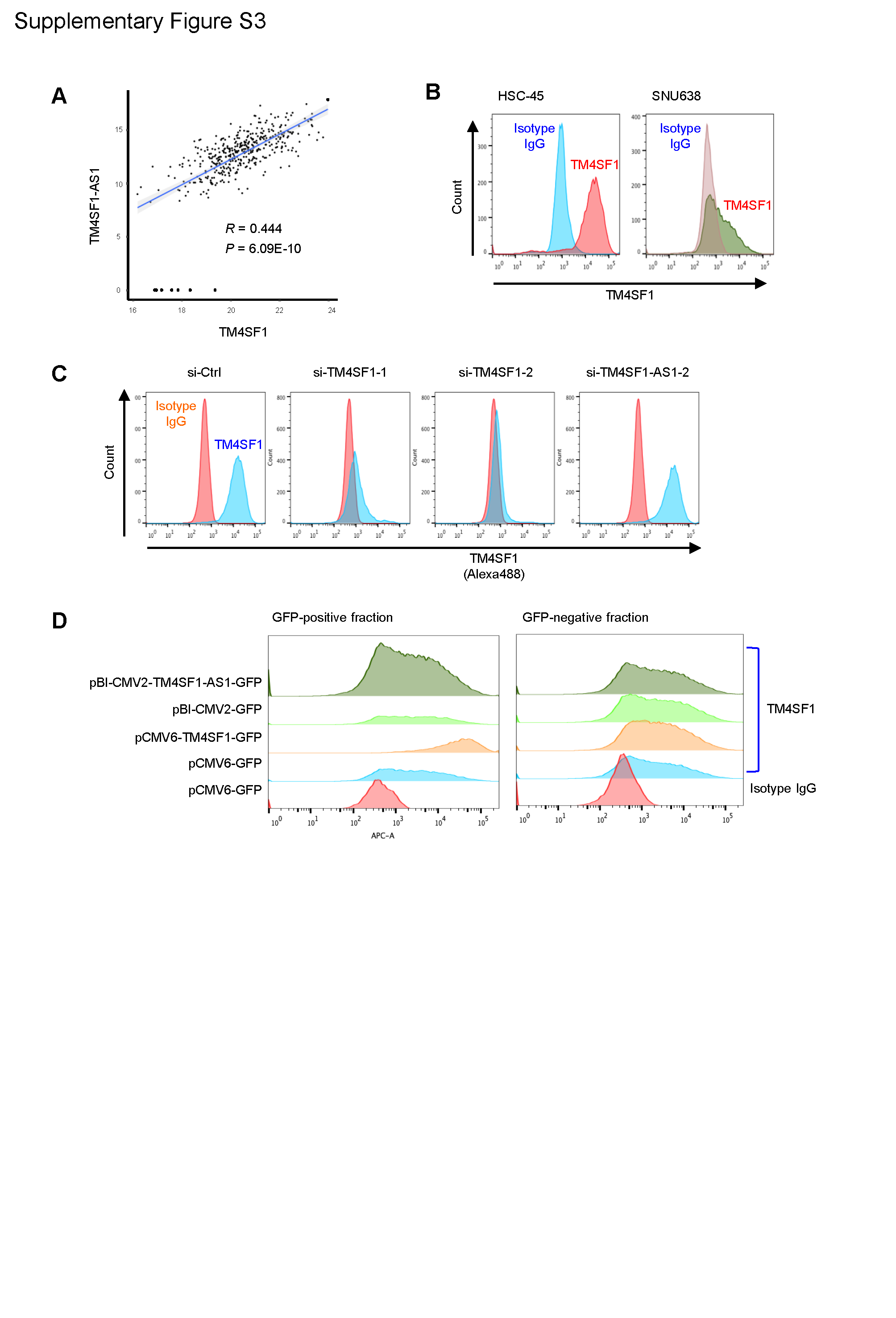


**Supplementary Figure S3**

TM4SF1 and TM4SF1-AS1 are co-expressed in GC cells, but TM4SF1-AS1 does not affect TM4SF1 expression. (A) Scatter plots showing expression levels of TM4SF1 and TM4SF1-AS1 in indicated datasets from TCGA. The Pearson correlation coefficients and P values are shown. (B) Flow cytometric analysis showing expression of TM4SF1 on the cell surface of the indicated GC cell lines. Isotype IgG served as a negative control. (C) Flow cytometric analysis showing expression of TM4SF1 on the surface of HSC-45 cells transfected with siRNAs targeting TM4SF1 (siTM4SF1-1 and -2) or TM4SF1-AS1 (si-TM4SF1-AS1-2) or a control siRNA (si-Ctrl). Note that siRNAs targeting TM4SF1 significantly depleted TM4SF1, while TM4SF1-AS1 knockdown did not affect TM4SF1 expression. (D) Flow cytometric analysis of TM4SF1 on the surface of SNU638 cells transfected with TM4SF1 or TM4SF1-AS1 expression vectors (pCMV6-TM4SF1-GFP or pBI-CMV2-TM4SF1-AS1-GFP) or control vectors (pCMV-GFP or pBI-CMV2-GFP). Results obtained with GFP-positive and ‑negative fractions are shown. Note that TM4SF1 was upregulated in cells transfected with pCMV6-TM4SF1-GFP, while expression of TM4SF1 did not change in cells with ectopic TM4SF1‑AS1 expression.


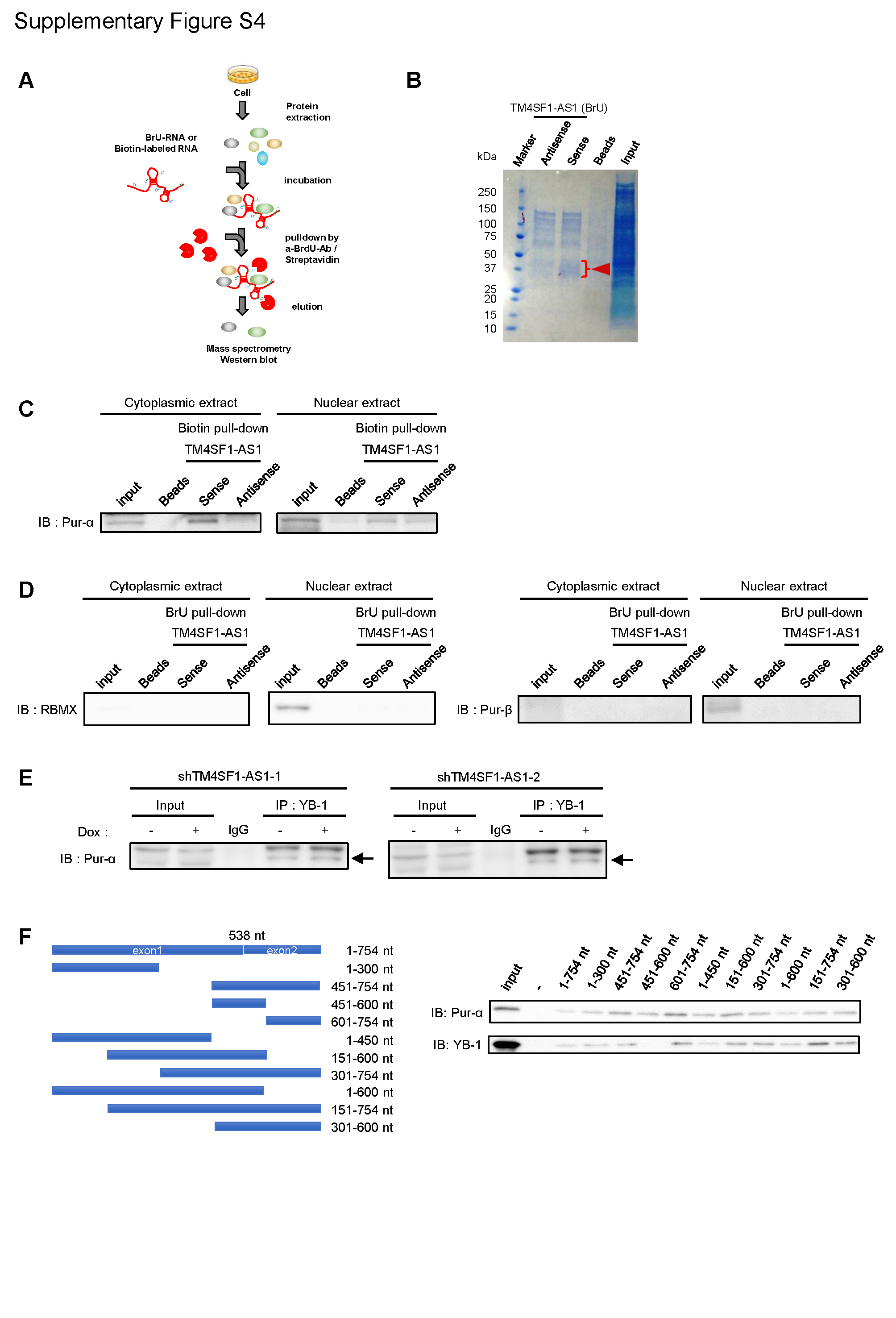


**Supplementary Figure S4**

Identification of proteins that interact with TM4SF1-AS1 in GC cells. (A) Workflow to identify proteins interacting with TM4SF1-AS1 in GC cells. Extracts from HSC-45 cells were pulled-down with BrU or biotin-labeled RNA, after which mass spectroscopy or western blot analyses were performed. (B) CBB staining of the proteins pulled-down with BrU-labeled TM4SF1-AS1, antisense of TM4SF1-AS1 (a negative control) or beads (mock). Bands specific to TM4SF1-AS1 (indicated by an arrow) were extracted and analyzed by mass spectrometry. (C) Extracts of subcellular fractions from HSC-45 cells were pulled-down with biotinylated TM4SF1-AS1 or its antisense and were probed for Pur-α. (D) Extracts of subcellular fractions of HSC-45 cells were pulled-down with BrU-labeled TM4SF1-AS1 or its antisense and were respectively probed for RBMX (left) or Pur-β (right). (E) Co-immunoprecipitation showing interaction between Pur-α and YB-1 in GC cells with TM4SF1-AS1 knockdown. HSC-45 cells with inducible shRNAs (1 and 2) targeting TM4SF1-AS1were incubated with or without Dox for 8 days. YB-1 in the cellular extracts was immunoprecipitated, after which western blotting was used to detect by co-precipitating Pur-α (indicated by arrows). (F) In vitro RNA pull-down showing interactions between deleted TM4SF1‑AS1 and Pur-α or YB-1. A series of TM4SF1-AS1 deletion mutants are indicated on the left. Myc-tagged Pur-α or YB-1 were pulled-down with biotin-labeled deletion mutants and were probed for Pur-α or YB-1(right).


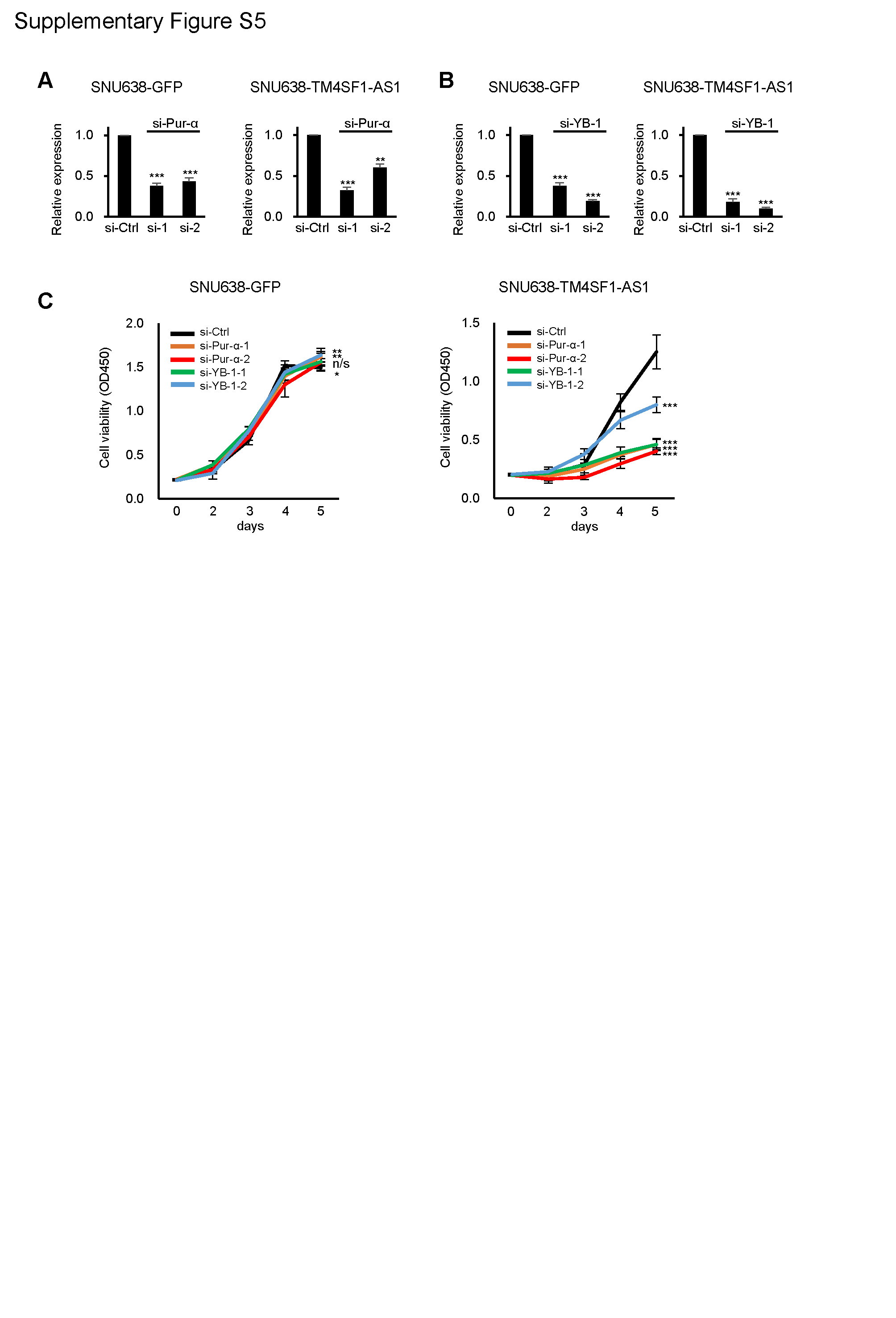


**Supplementary Figure S5**

Depletion of Pur-α or YB-1 attenuates growth of GC cells expressing TM4SF1-AS1. (A, B) qRT-PCR analysis of Pur-α (A) and YB-1 (B) in SNU638-GFP and SNU638-TM4SF1-AS1 cells transfected with the indicated siRNAs (*n* = 3). (C) Results of cell viability assays with SNU638-GFP and SNU638-TM4SF1-AS1 cells transfected with the indicated siRNAs (*n* = 8). **P*<0.05, ***P*<0.01, ****P*<0.001.


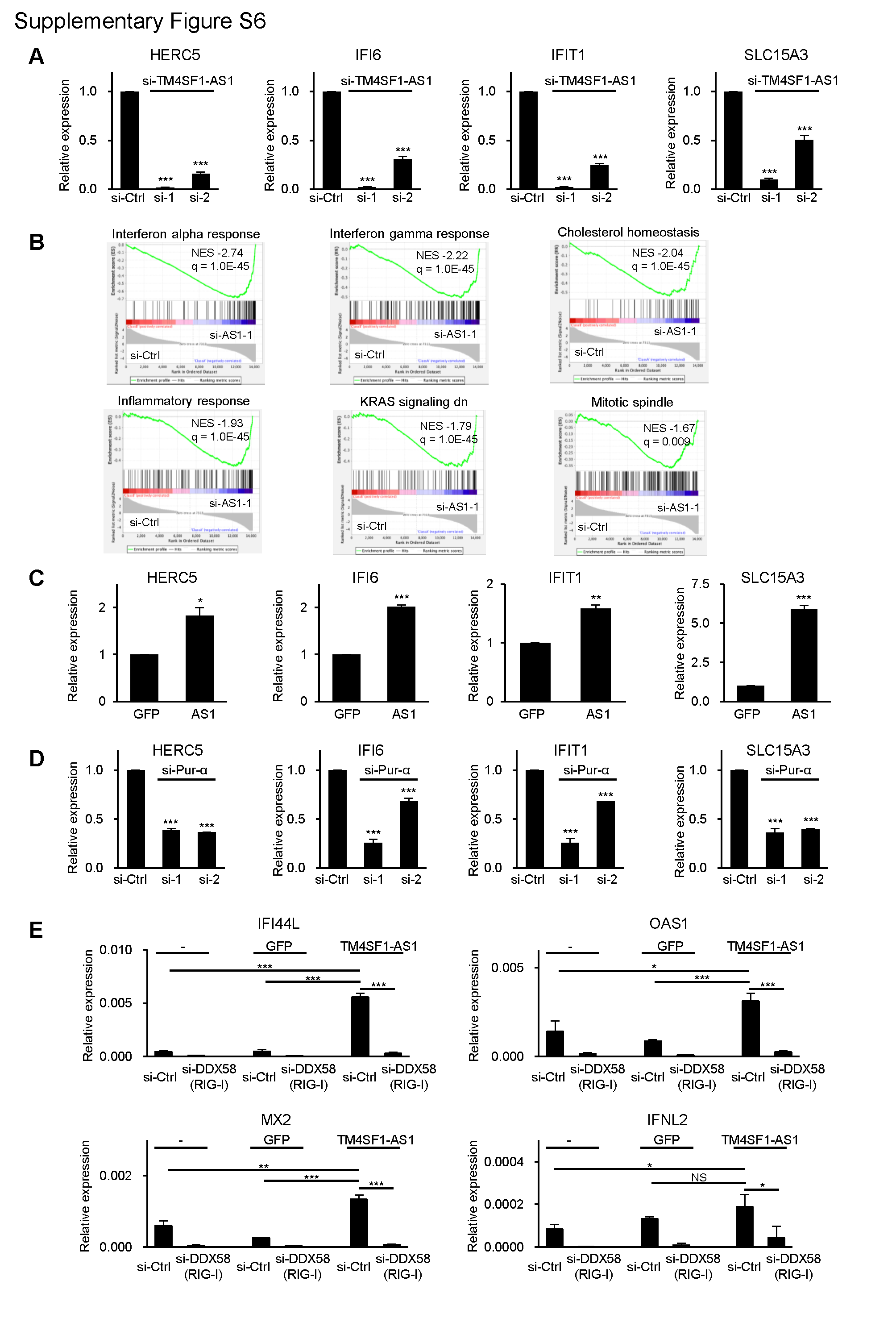


**Supplementary Figure S6**

TM4SF1-AS1 activates interferon signaling in GC cells. (A) qRT-PCR analysis of the indicated interferon-stimulated genes (ISGs) in HSC-45 cells transfected with a control siRNA or siRNAs targeting TM4SF1-AS1. (B) Results of a GSEA using microarray data obtained from HSC-45 cells after TM4SF1 knockdown. (C) qRT-PCR analysis of the indicated ISGs in SNU638-GFP and SNU638-TM4SF1-AS1 cells. (D) qRT-PCR analysis of the indicated ISGs in SNU638-AS1 cells transfected with a control siRNA or siRNAs targeting Pur-α. (E) qRT-PCR analysis of the indicated IRDS gene in SNU638, SNU638-GFP and SNU638-TM4SF1-AS1 cells transfected with a control siRNA or a siRNA targeting DDX58 (RIG-I). **P*<0.05, ***P*<0.01, ****P*<0.001, NS, not significant.


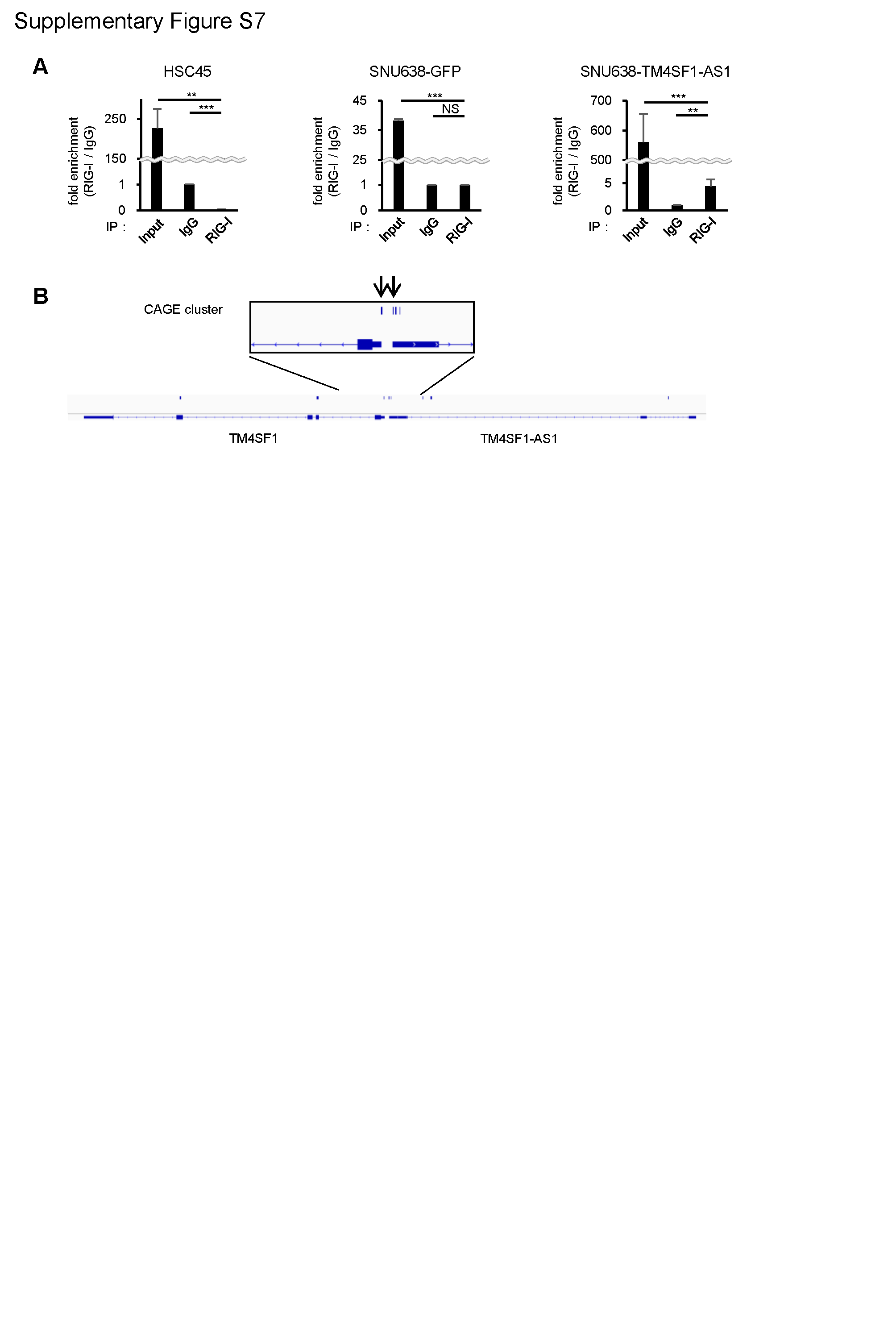


**Supplementary Figure S7**

TM4SF1-AS1 may not directly interact with RIG-I in GC cells. (A) Results of RIP-qPCR assays. RIG-I expressed in the indicated GC cells was immunoprecipitated, and co-precipitated TM4SF1‑AS1 was detected by qRT-PCR. IgG served as a negative control (*n* = 3). (B) Cap analysis of gene expression sequencing (CAGE-seq) results of TM4SF1 and TM4SF1-AS1 obtained from the functional annotation of the mammalian genome (FANTOM) dataset. Arrows indicate CAGE clusters representing transcriptional start sites of capped mRNAs. ***P*<0.01, ****P*<0.001, NS, not significant.


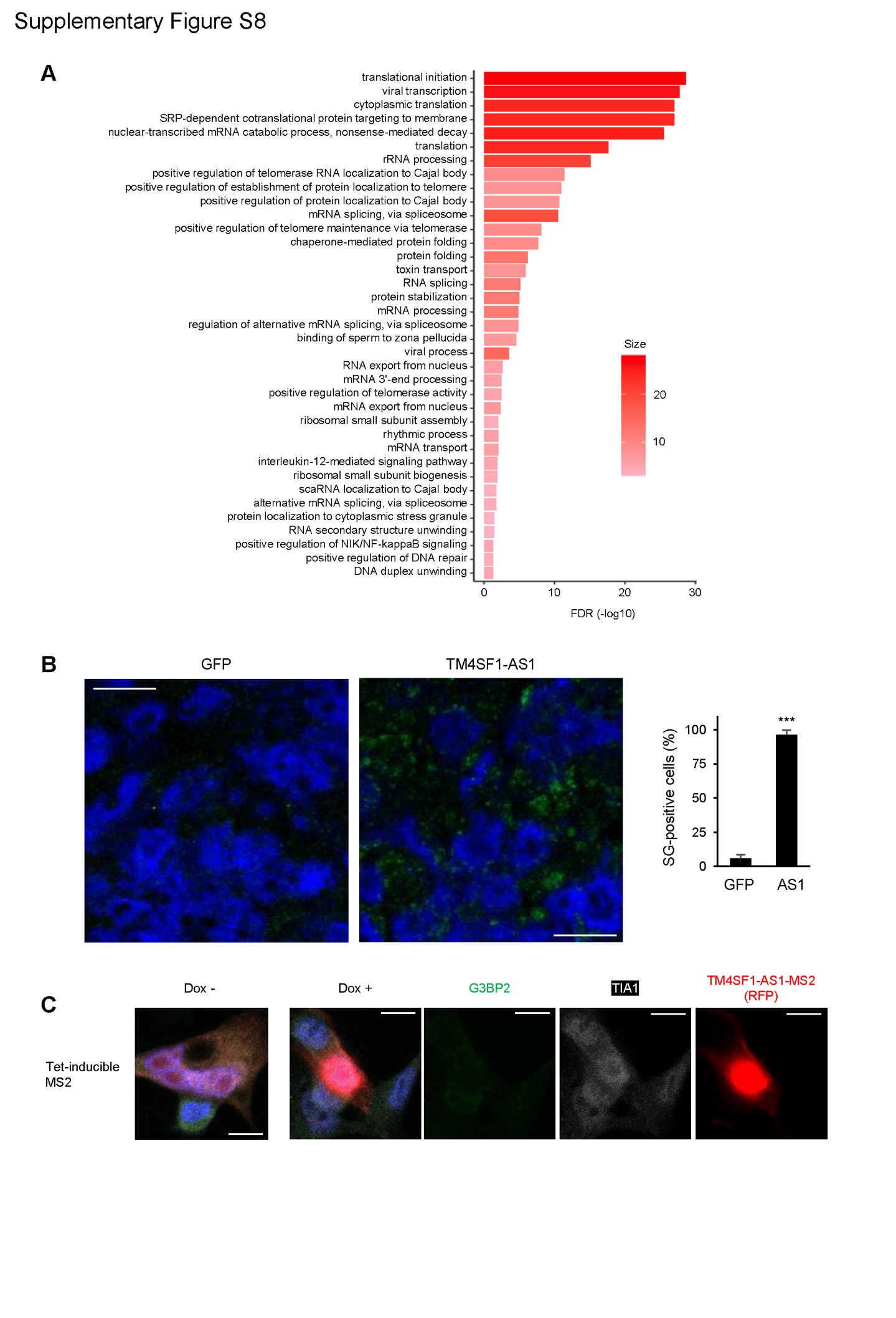


**Supplementary Figure S8**

TM4SF1-AS1 upregulates SGs in GC cells. (A) GO analysis of proteins identified by ChIRP-MS analysis. (B) Immunofluorescent staining of G3BP2 in xenograft tumors derived from SNU638 cells stably expressing GFP or TM4SF1-AS1. Summarized results are shown on the right (*n* =7). Scale bars = 10 μm. (C) Immunofluorescent staining of G3BP2 (green), TIA1 (white) and inducible MS2-tag (red) in SNU638 cells. Cells were transfected with a MS2 coat protein (MCP)-RFP plasmid and incubated for 8 days with or without Dox. Scale bars = 10 μm.


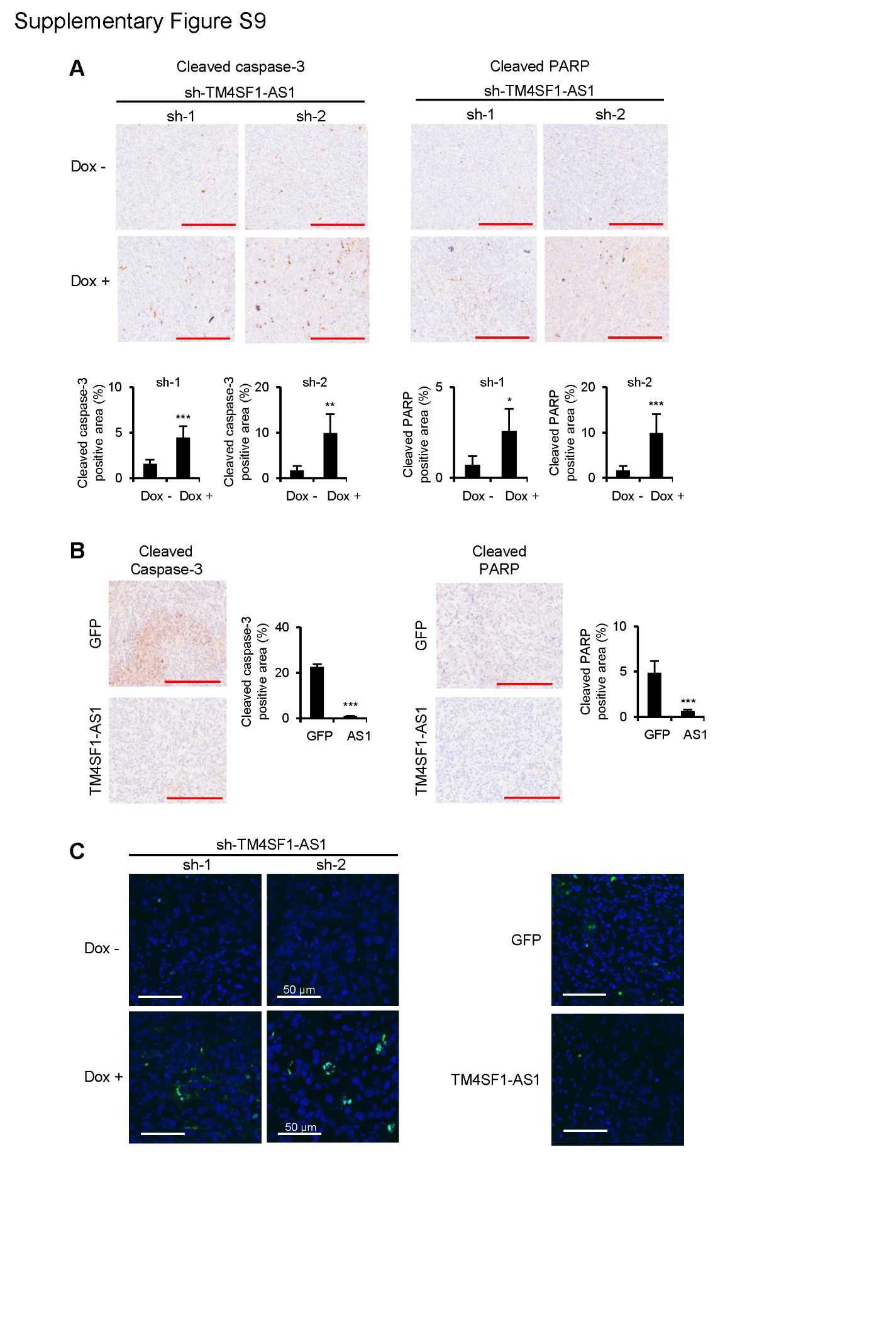


**Supplementary Figure S9**

TM4SF1-AS1 suppresses apoptosis in vivo. (A) Immunohistochemical analysis of cleaved caspase‑3 or cleaved PARP in the xenograft tumors in Figure 2E. Mice were injected with HSC-45 cells expressing inducible shRNAs targeting TM4SF-1AS1 (sh-1 and sh-2) and were treated with or without Dox. Representative images are shown at the top. Summarized results from 7 randomly selected fields are shown below; error bars represent SDs. Scale bars = 500 μm. (B) Immunohistochemical analysis of cleaved caspase-3 or cleaved PARP in the xenograft tumors in Figure 2G. Mice were injected with SNU638 cells stably expressing GFP or TM4SF1-AS1. Summarized results from seven randomly selected fields are shown below; error bars represent SDs. Scale bars = 250 μm. (C) Fluorescence images of TUNEL in the xenograft tumors in Figure 2E (left) and 2G (right). Scale bars = 50 μm. **P*<0.05, ***P*<0.01, ****P*<0.001.


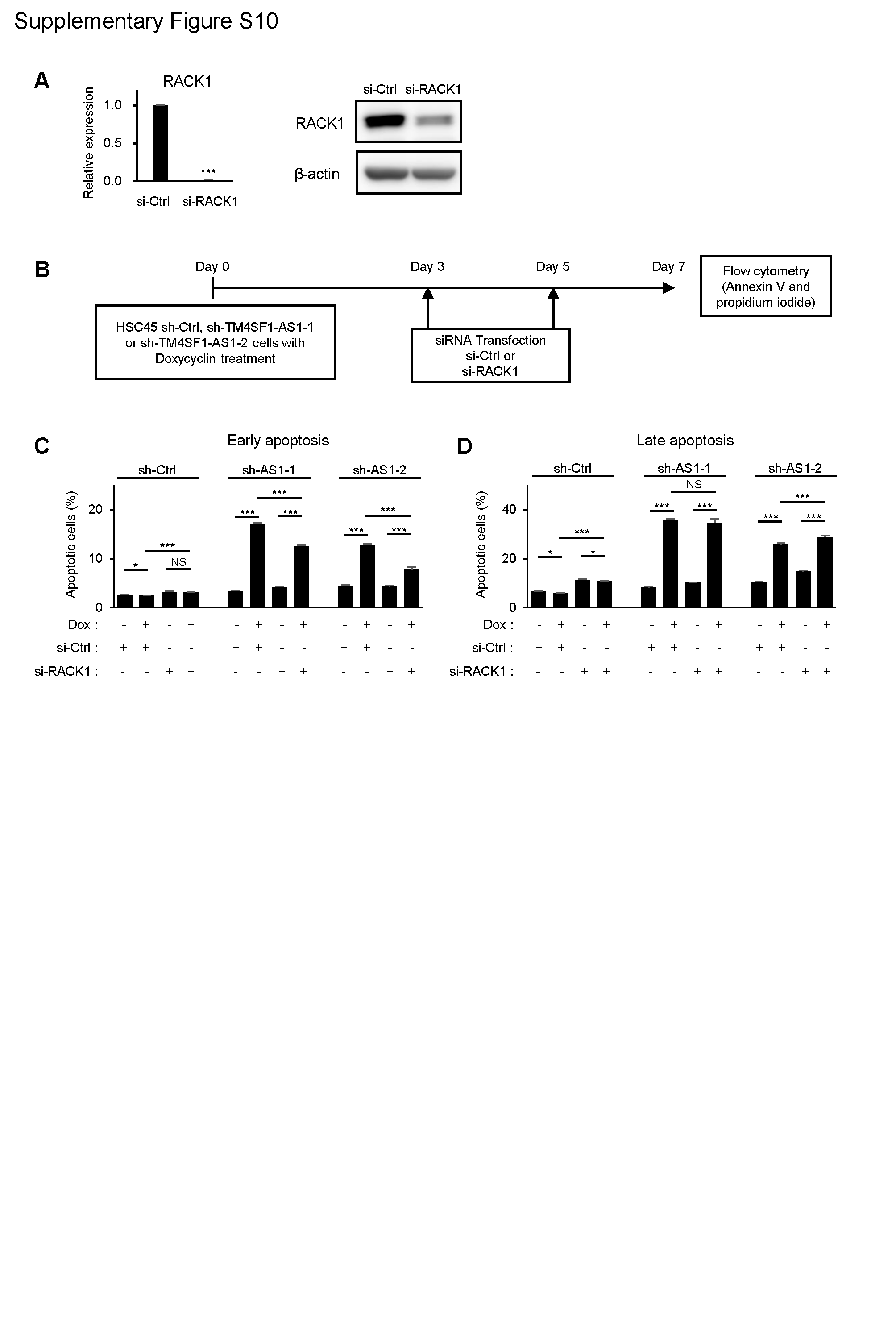


**Supplementary Figure S10**

Suppression of RACK1 attenuates early apoptosis induced by TM4SF1-AS1 depletion in GC cells. (A) qRT-PCR (left) and western blot analysis (right) of RACK1 in HSC-45 cells transfected with a siRNA targeting RACK1 or a control siRNA (si-Ctrl). (B) Workflow of apoptosis assays in HSC-45 cells with double knockdown of TM4SF1-AS1 and RACK1. HSC-45 cells expressing inducible shRNAs targeting TM4SF1-AS1 (sh-AS1 or sh-AS2) or a control shRNA (sh-Ctrl) were treated with or without Dox, after which cells were transfected with a siRNA targeting RACK1 or a control siRNA (si-Ctrl) at indicated time points. (C, D) Summarized results of early (left) and late apoptosis (right). (n=4). **P*<0.05, ***P*<0.01, ****P*<0.001.


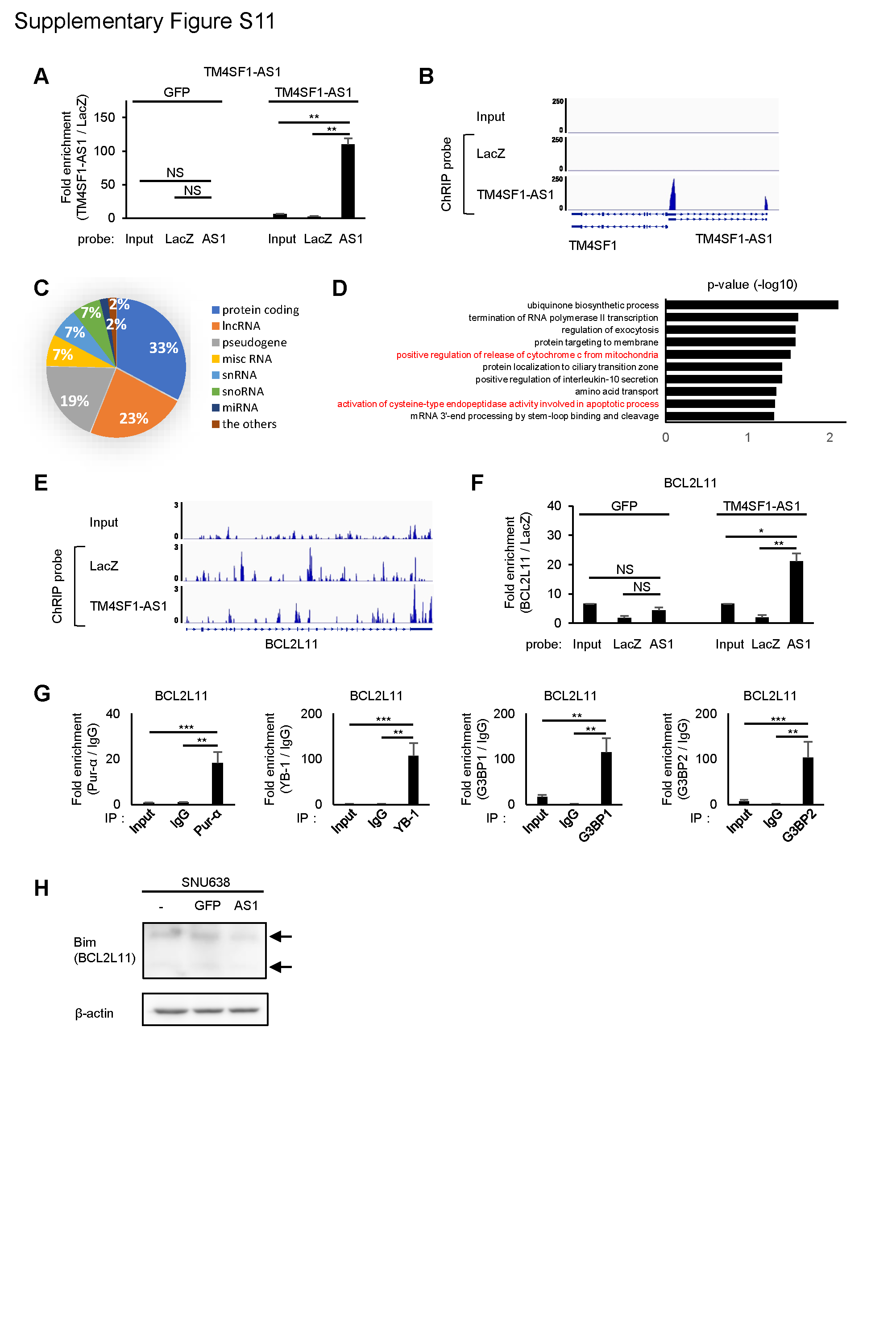


**Supplementary Figure S11**

Identification of RNAs associating with TM4SF1-AS1 in GC cells. (A) qRT-PCR confirming enrichment of TM4SF1-AS1 in ChIRP products derived from SNU638‑GFP (left) and SNU638‑TM4SF1-AS1 cells (right). (B) Results of ChIRP-RNA-seq for TM4SF1-AS1 derived from SNU638‑TM4SF1-AS1 cells. Gene structures of TM4SF1 and TM4SF1-AS1 are shown below. (C) Types of RNAs identified by ChIRP-RNA-seq analysis. (D) Gene ontology analysis of the protein-coding genes identified by ChIRP-RNA-seq. (E) ChIRP-RNA-seq results for the BCL2L11 gene. (F) qRT-PCR confirming enrichment of BCL2L11 in ChIRP products derived from SNU638‑GFP (left) and SNU638‑TM4SF1-AS1 cells (right). (G) RIP-qPCR assays showing association between BCL2L11 mRNA and Pur-α, YB-1, G3BP1 or G3BP2 proteins in GC cells. The indicated proteins in HSC-45 cells were immunoprecipitated, and co-precipitated BCL2L11 was detected by qRT-PCR. IgG served as a negative control. (H) Western blot analysis of Bim (BCL2L11) in SNU638, SNU638-GFP, and SNU638-TM4SF1-AS1 cells.
