# Supplementary material for: TM4SF1-AS1 inhibits apoptosis by promoting stress granule formation in cancer cells
Source: Cell Death Dis. 2023 Jul 13;14(7):424. doi: 10.1038/s41419-023-05953-3 (PMC10345132; doi:10.1038/s41419-023-05953-3)

Kitajima *et al*. Original images of western blots

Figure 3

B

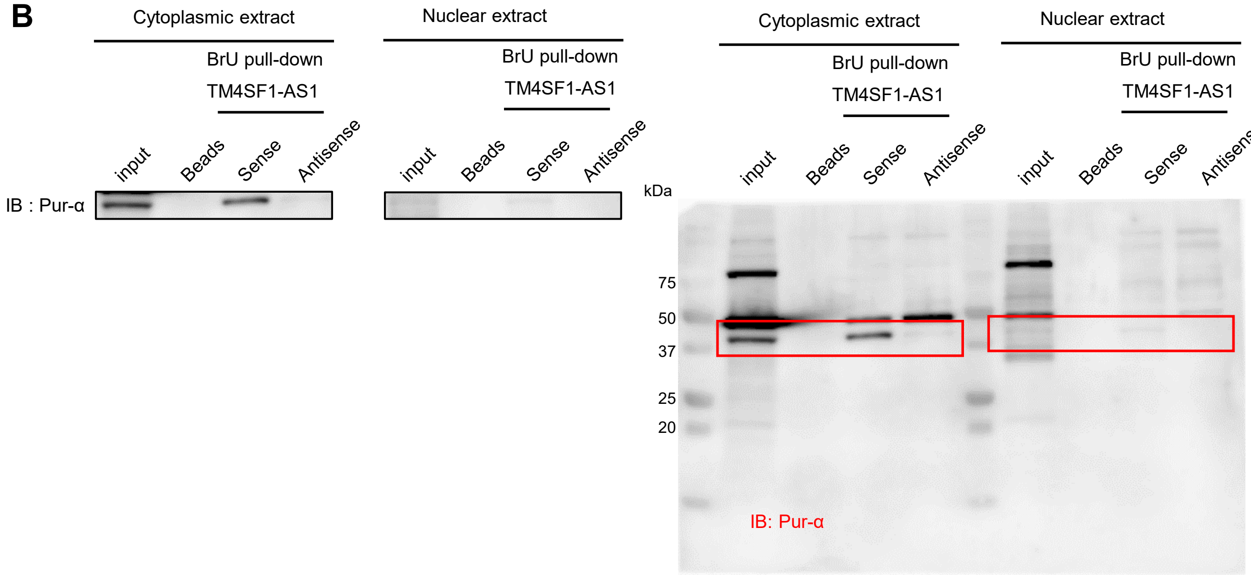

C

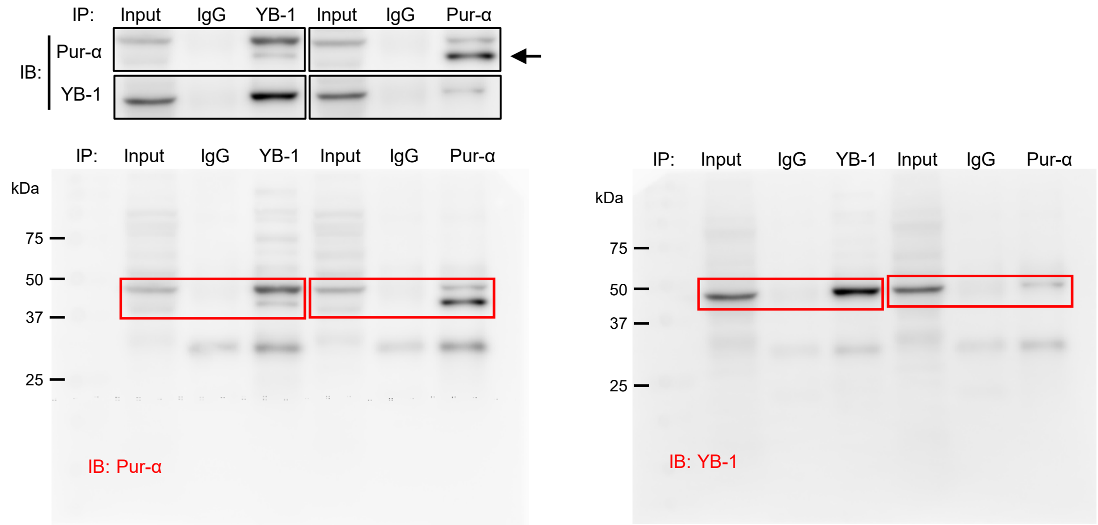

D

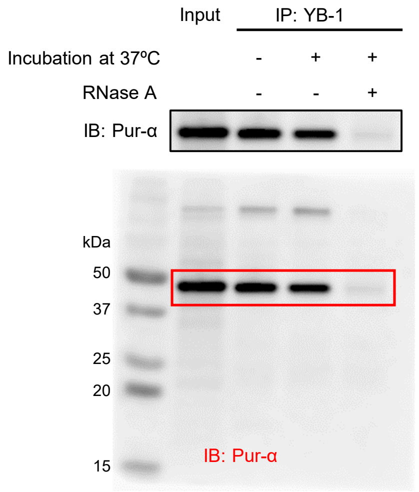

Kitajima *et al*. Original images of western blots

Figure 3

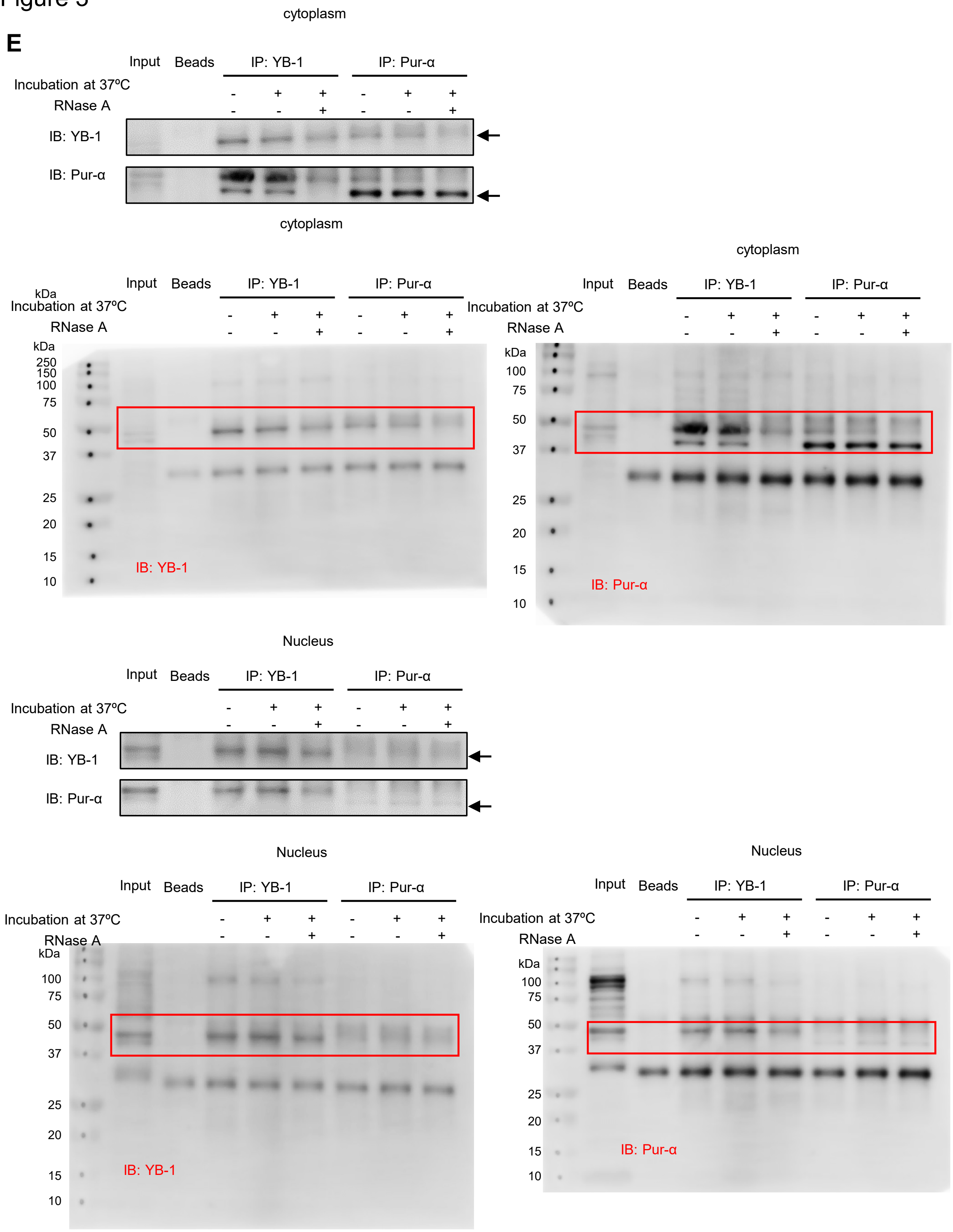

Kitajima *et al*. Original images of western blots

Figure 4

E

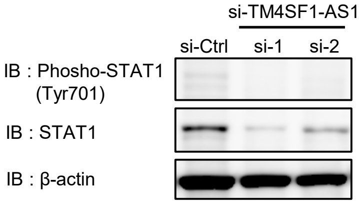

H

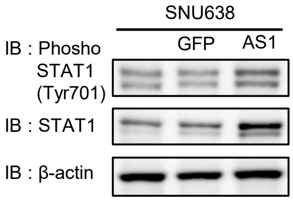

H **E**

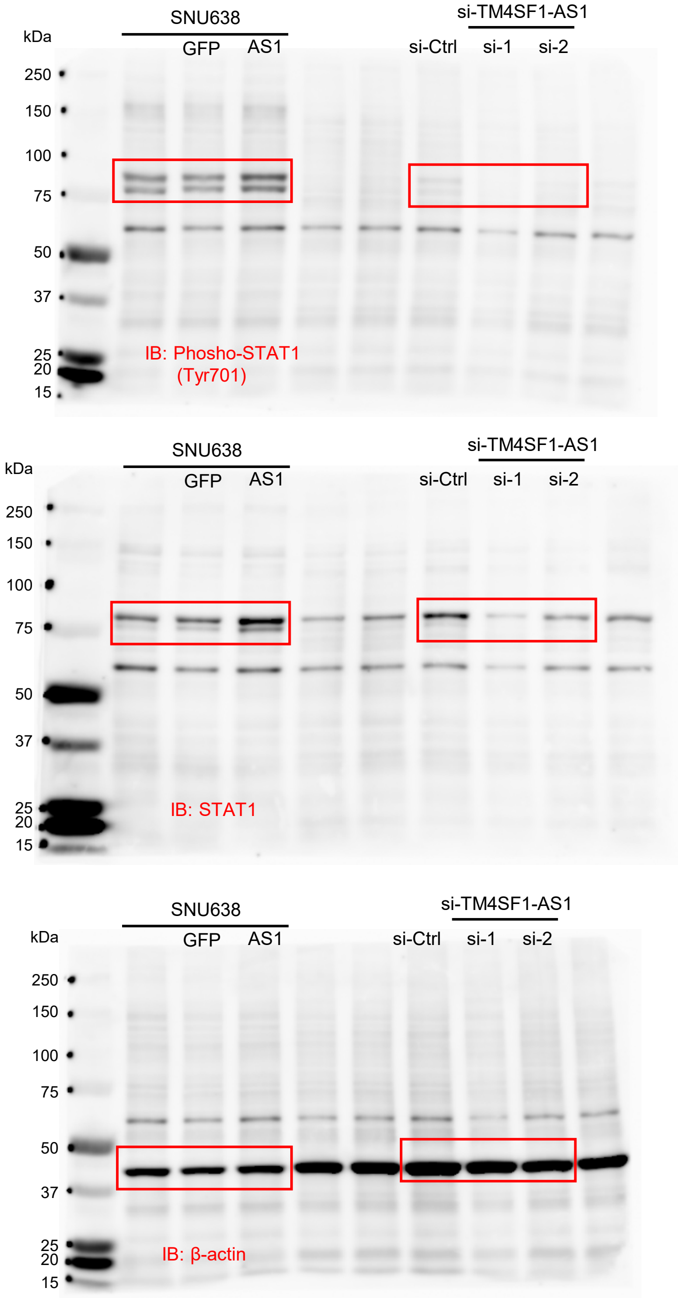

Kitajima *et al*. Original images of western blots

Figure 4

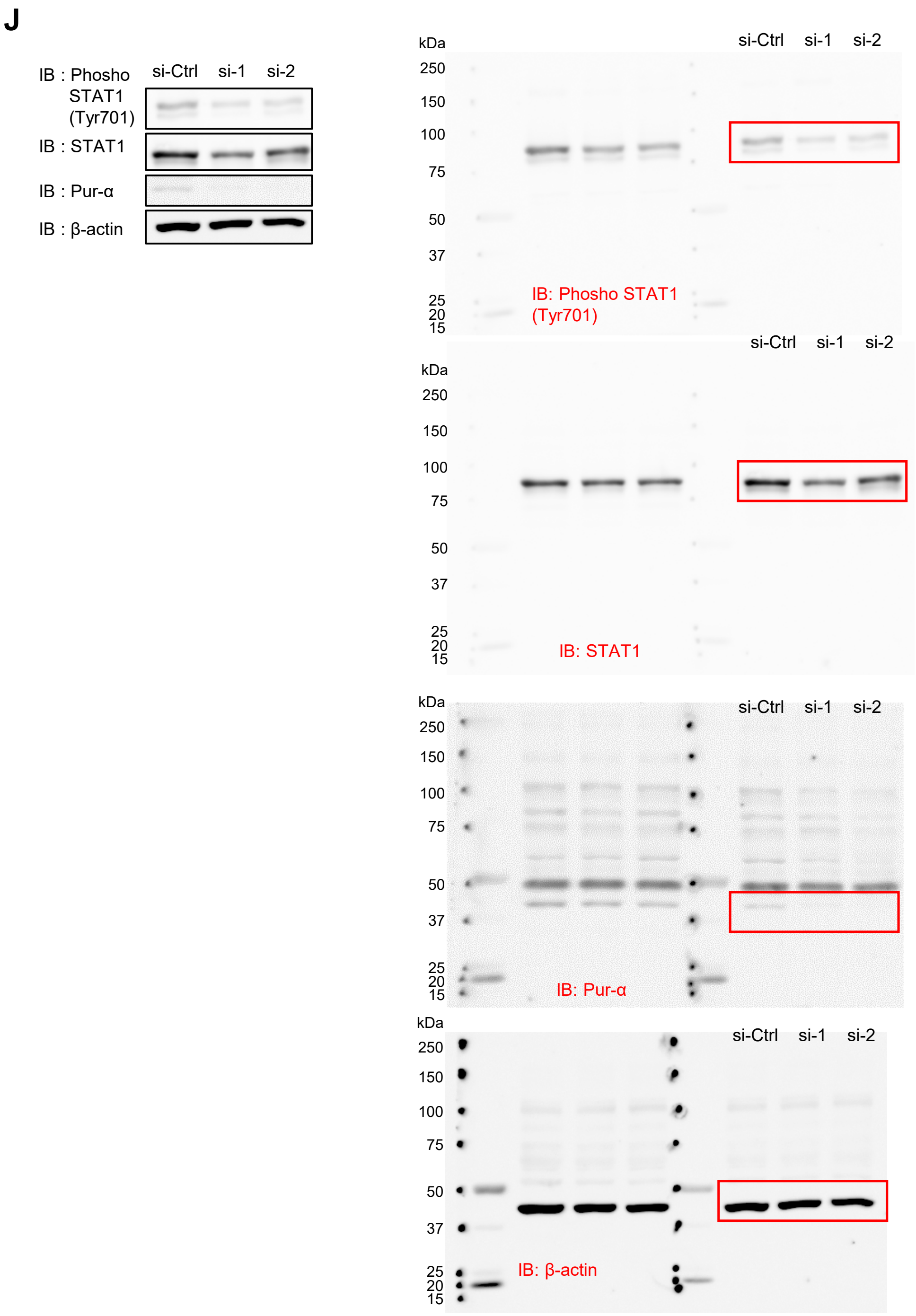

Kitajima *et al*. Original images of western blots

Figure 6

E

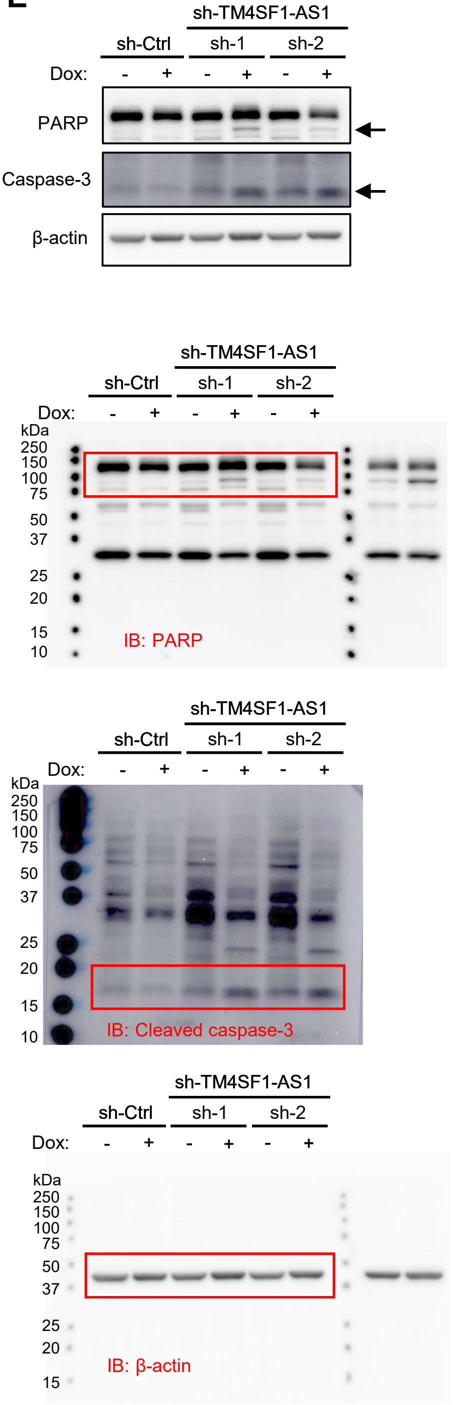

F

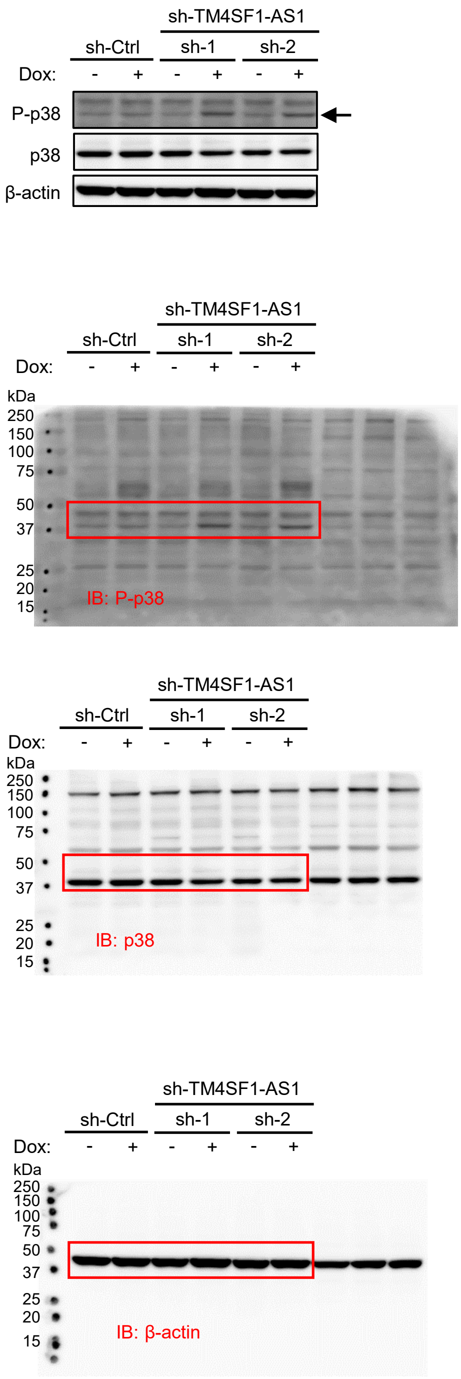

Kitajima *et al*. Original images of western blots

Figure 7

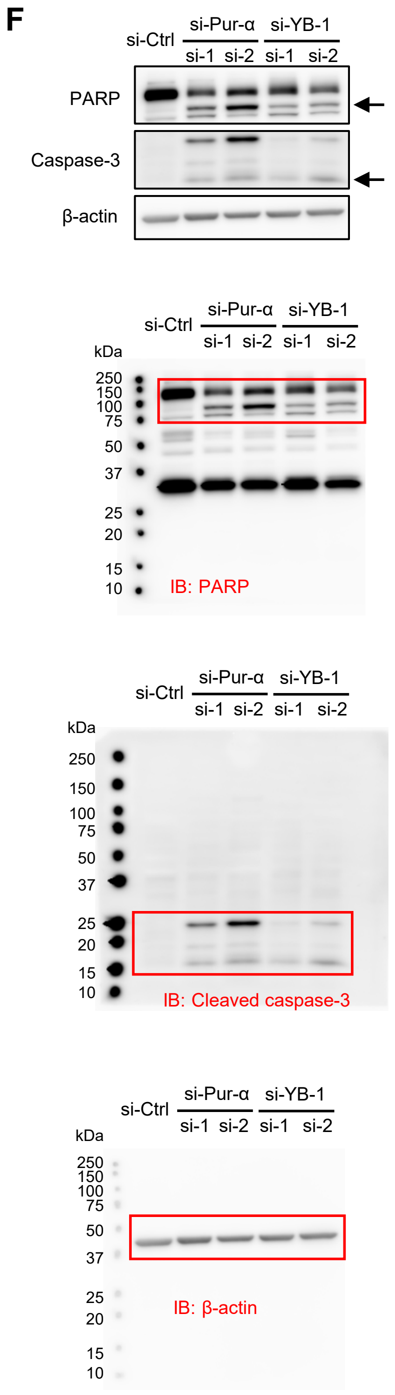

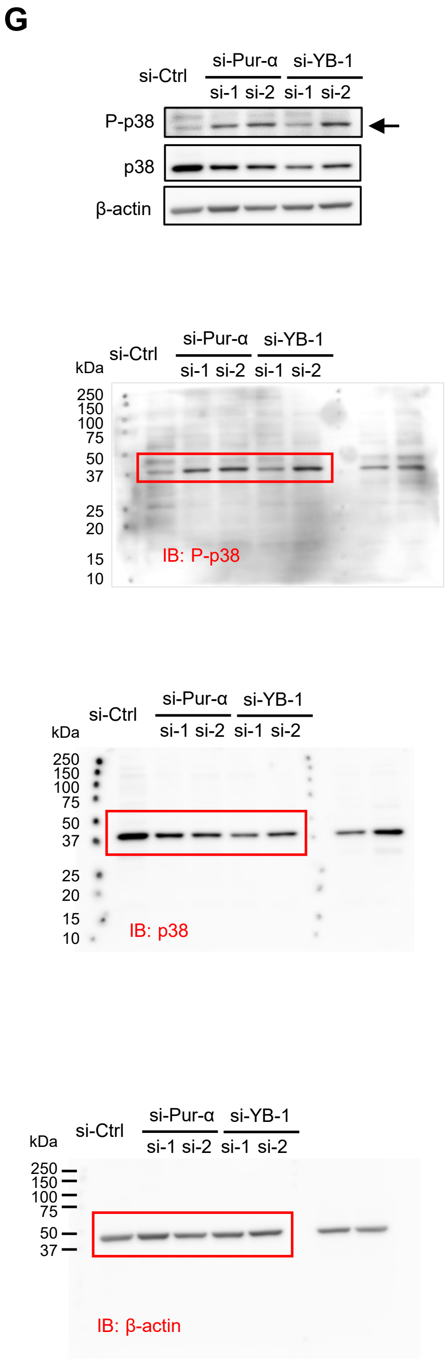

Kitajima *et al*. Original images of western blots

Supplementary Figure S4

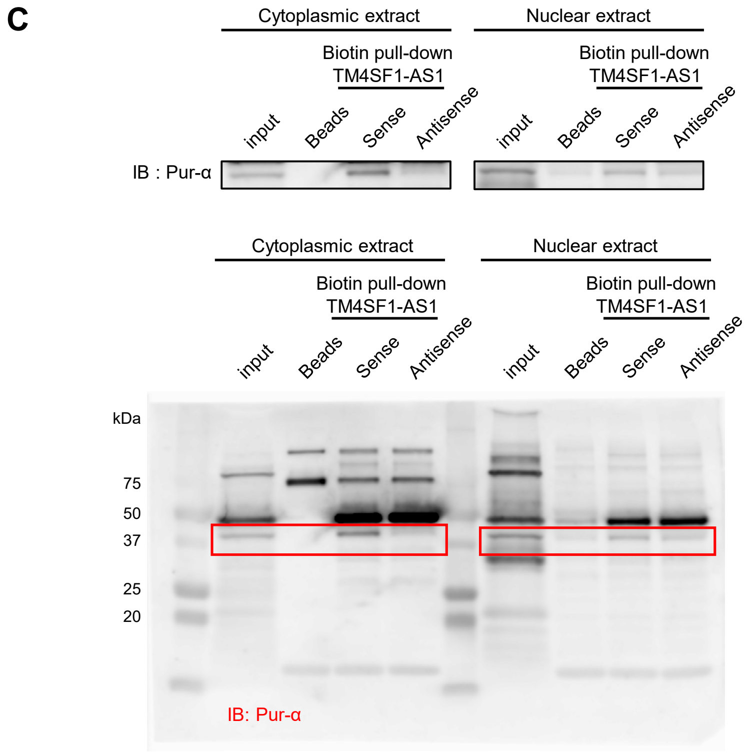

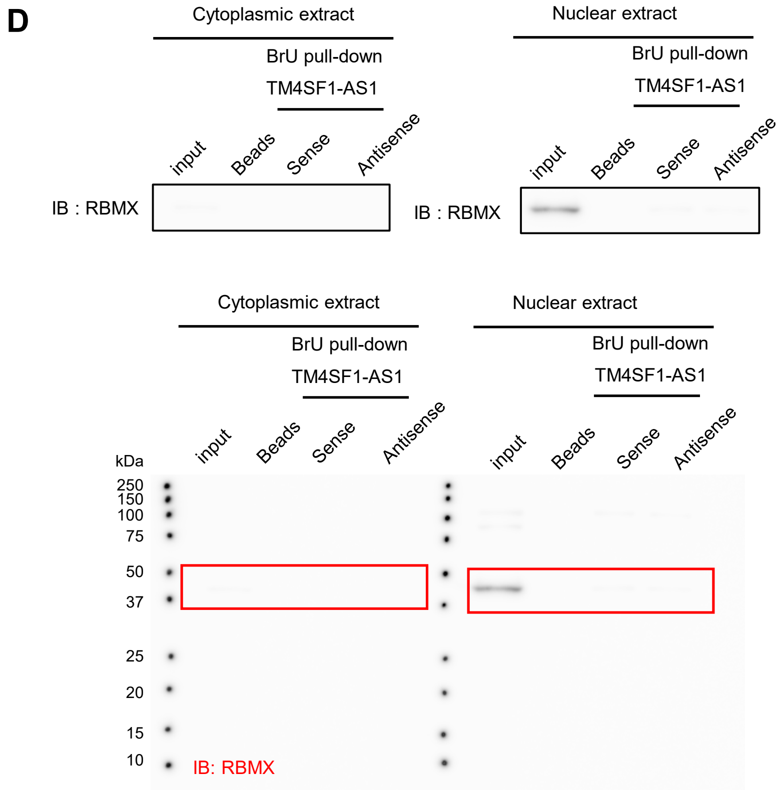

Kitajima *et al*. Original images of western blots

Supplementary Figure S4

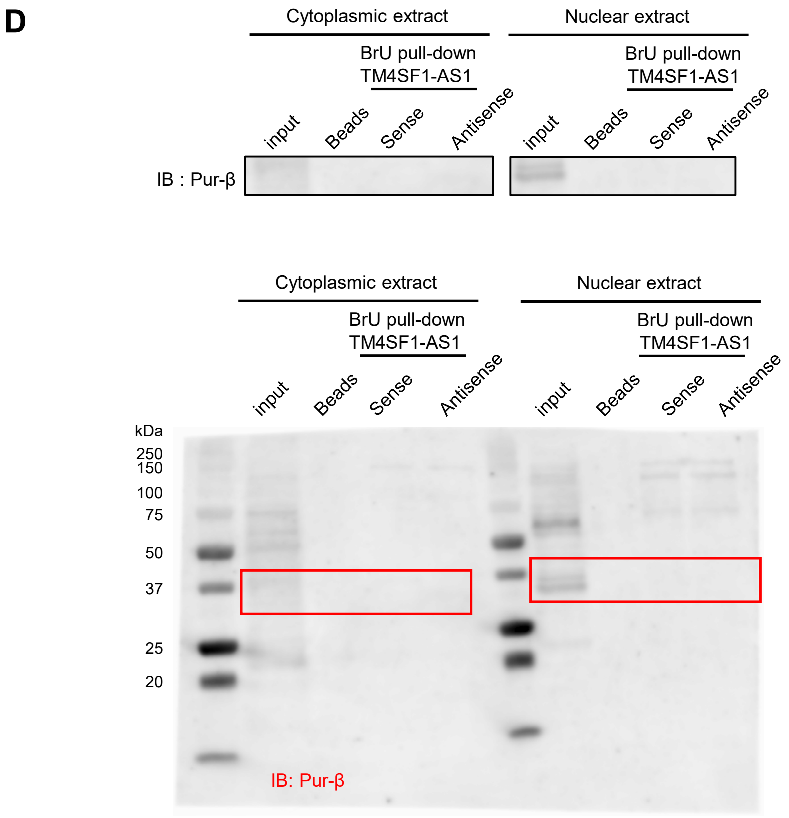

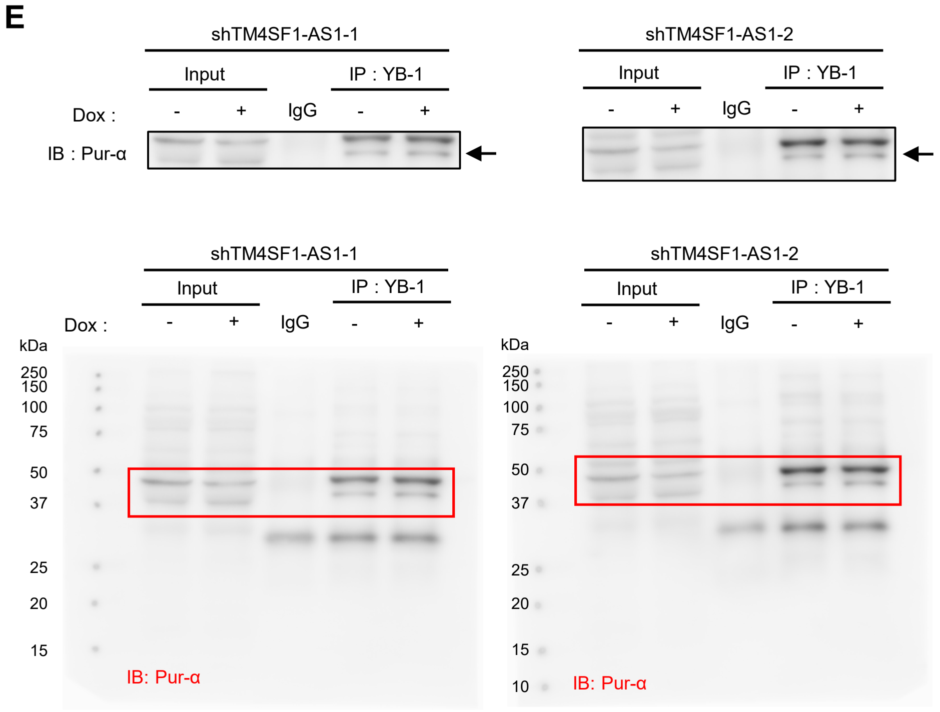

Supplementary Figure S4

F

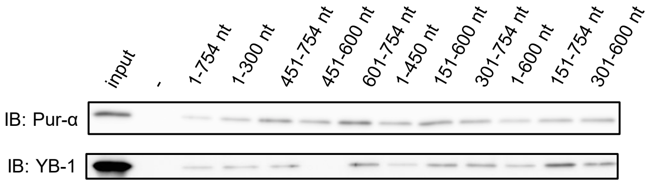

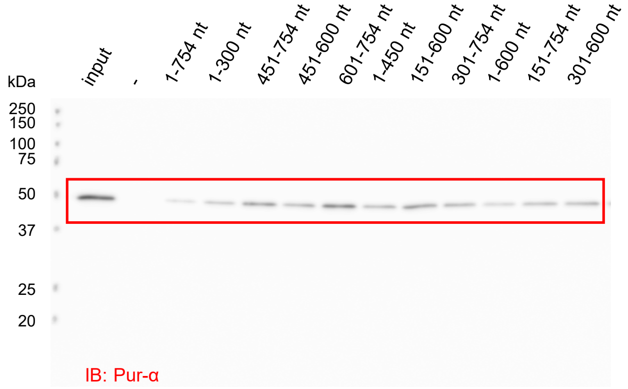

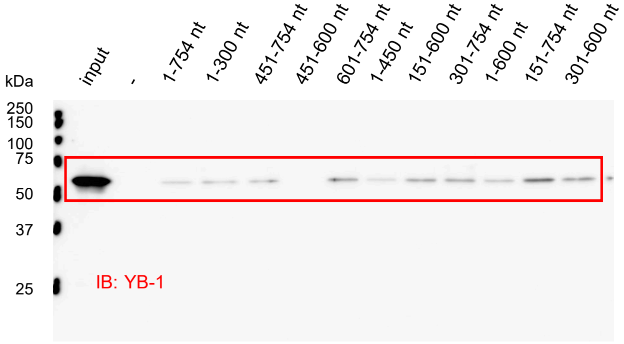

Kitajima *et al*. Original images of western blots

Supplementary Figure S10

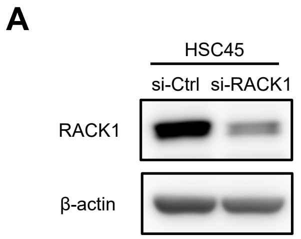

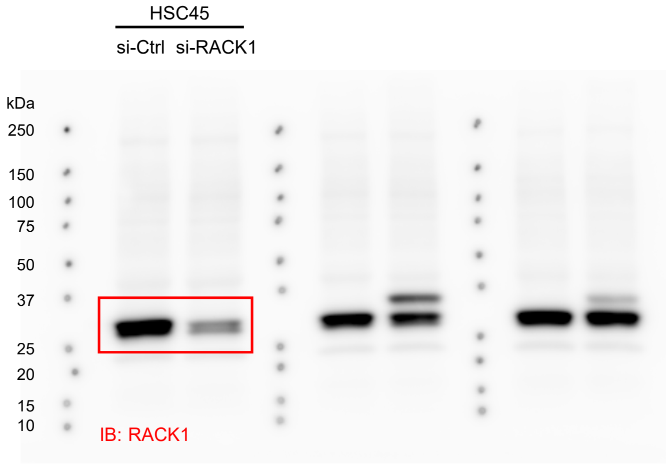

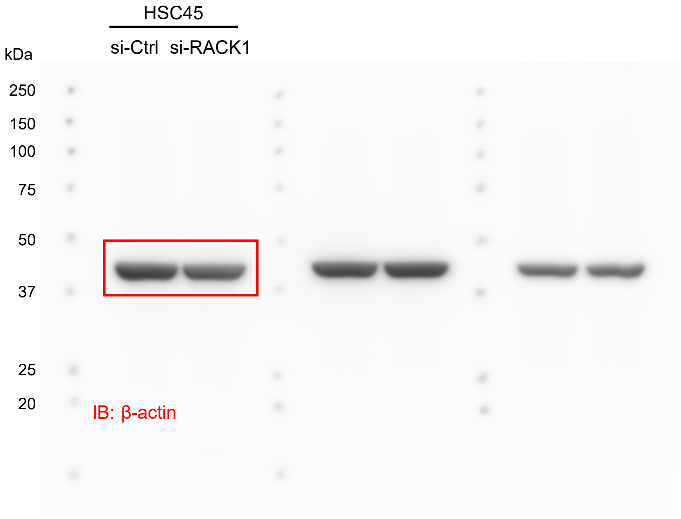

Supplementary Figure S11

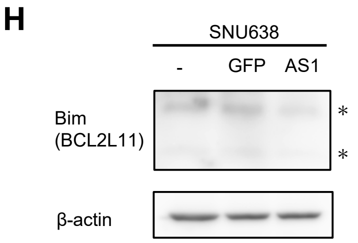

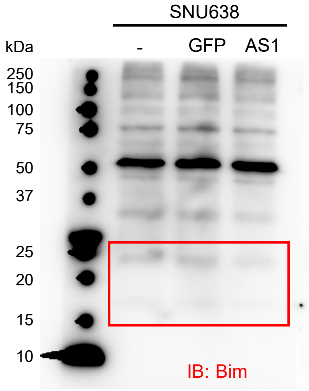

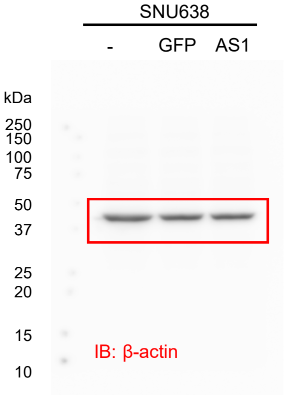

Supplement: Supplementary file 4 — Original images of western blots [file 41419_2023_5953_MOESM4_ESM.pdf]
